# Association of pre-pregnancy body mass index with offspring metabolic profile: Analyses of 3 European prospective birth cohorts

**DOI:** 10.1371/journal.pmed.1002376

**Published:** 2017-08-22

**Authors:** Diana L. Santos Ferreira, Dylan M. Williams, Antti J. Kangas, Pasi Soininen, Mika Ala-Korpela, George Davey Smith, Marjo-Riitta Jarvelin, Debbie A. Lawlor

**Affiliations:** 1 MRC Integrative Epidemiology Unit at the University of Bristol, Bristol, United Kingdom; 2 School of Social and Community Medicine, University of Bristol, United Kingdom; 3 Department of Epidemiology and Biostatistics, MRC–PHE Centre for Environment and Health, School of Public Health, Imperial College London, London, United Kingdom; 4 Department of Medical Epidemiology & Biostatistics, Karolinska Institutet, Stockholm; 5 Computational Medicine, Faculty of Medicine, University of Oulu, Oulu, Finland; 6 Biocenter Oulu, University of Oulu, Oulu, Finland; 7 NMR Metabolomics Laboratory, School of Pharmacy, University of Eastern Finland, Kuopio, Finland; 8 Center for Life-Course Health Research and Northern Finland Cohort Center, Faculty of Medicine, University of Oulu, Oulu, Finland; 9 Unit of Primary Care, Oulu University Hospital, Oulu, Finland; Chinese University of Hong Kong, CHINA

## Abstract

**Background:**

A high proportion of women start pregnancy overweight or obese. According to the developmental overnutrition hypothesis, this could lead offspring to have metabolic disruption throughout their lives and thus perpetuate the obesity epidemic across generations. Concerns about this hypothesis are influencing antenatal care. However, it is unknown whether maternal pregnancy adiposity is associated with long-term risk of adverse metabolic profiles in offspring, and if so, whether this association is causal, via intrauterine mechanisms, or explained by shared familial (genetic, lifestyle, socioeconomic) characteristics. We aimed to determine if associations between maternal body mass index (BMI) and offspring systemic cardio-metabolic profile are causal, via intrauterine mechanisms, or due to shared familial factors.

**Methods and findings:**

We used 1- and 2-stage individual participant data (IPD) meta-analysis, and a negative-control (paternal BMI) to examine the association between maternal pre-pregnancy BMI and offspring serum metabolome from 3 European birth cohorts (offspring age at blood collection: 16, 17, and 31 years). Circulating metabolic traits were quantified by high-throughput nuclear magnetic resonance metabolomics. Results from 1-stage IPD meta-analysis (*N* = 5327 to 5377 mother-father-offspring trios) showed that increasing maternal and paternal BMI was associated with an adverse cardio-metabolic profile in offspring. We observed strong positive associations with very-low-density lipoprotein (VLDL)-lipoproteins, VLDL-cholesterol (C), VLDL-triglycerides, VLDL-diameter, branched/aromatic amino acids, glycoprotein acetyls, and triglycerides, and strong negative associations with high-density lipoprotein (HDL), HDL-diameter, HDL-C, HDL_2_-C, and HDL_3_-C (all *P* < 0.003). Slightly stronger magnitudes of associations were present for maternal compared with paternal BMI across these associations; however, there was no strong statistical evidence for heterogeneity between them (all bootstrap *P* > 0.003, equivalent to *P* > 0.05 after accounting for multiple testing). Results were similar in each individual cohort, and in the 2-stage analysis. Offspring BMI showed similar patterns of cross-sectional association with metabolic profile as for parental pre-pregnancy BMI associations but with greater magnitudes. Adjustment of parental BMI–offspring metabolic traits associations for offspring BMI suggested the parental associations were largely due to the association of parental BMI with offspring BMI. Limitations of this study are that inferences cannot be drawn about the role of circulating maternal fetal fuels (i.e., glucose, lipids, fatty acids, and amino acids) on later offspring metabolic profile. In addition, BMI may not reflect potential effects of maternal pregnancy fat distribution.

**Conclusion:**

Our findings suggest that maternal BMI–offspring metabolome associations are likely to be largely due to shared genetic or familial lifestyle confounding rather than to intrauterine mechanisms.

## Abstract 2

Debbie Lawlor and colleagues assess the link between maternal pregnancy adiposity and disrupted childhood cardio-metabolic profiles and show that shared familial factors are responsible rather than intrauterine ones.

## Author summary

**Why was this study done?:**

- It is unknown whether maternal pregnancy adiposity is associated with long-term risk of adverse metabolic profiles in offspring, and if so, whether this association is causal via intrauterine mechanisms or explained by shared familial (genetic, lifestyle, socioeconomic) characteristics.
- Our study was designed to unpick whether maternal pregnancy BMI–offspring metabolic traits associations were due to intrauterine mechanisms (involving overfeeding of the developing fetus) or shared familial characteristics (or both of these) by using paternal BMI–offspring metabolic traits associations as a negative control.
- If maternal associations represent causal intrauterine effects, as opposed to being due to shared familial factors, they should be stronger than paternal associations with the same metabolic traits.

**What did the researchers do and find?:**

- We looked at associations between maternal pre-pregnancy BMI and 153 offspring serum metabolic traits, in adolescence and adulthood, in 3 independent European birth cohorts (>10,000) and compared these to paternal BMI–offspring metabolic traits as a negative control.
- Paternal BMI was used as a negative control as it will share most of the confounders of the main exposure (maternal BMI) but could not directly influence offspring’s metabolic traits via an intrauterine effect.
- We found very little evidence of a markedly stronger association of maternal BMI with offspring outcomes.

**What do these findings mean?:**

- Our findings are more supportive of shared familial factors than intrauterine developmental overnutrition mechanisms for associations of maternal BMI with offspring metabolic traits.
- Interventions to reduce BMI in all family members may be more beneficial for cardio-metabolic health than focusing on reducing maternal pre-conception or pregnancy BMI.

## Introduction

In Western populations, the proportion of women who start pregnancy overweight or obese (body mass index [BMI] ≥25 kg/m^2^) has increased over the last 20–30 years and is now estimated to be between 20%–50% [1,2].

The developmental origin of adult diseases hypothesis proposes that greater maternal adiposity in pregnancy can prime changes in fetal metabolism that result in a life-long risk of greater adiposity and metabolic dysregulation [3]. As more adipose women have higher circulating gestational glucose, lipids, and fatty acids (FAs), the fetus is purportedly overfed, which may lead to changes in energy metabolism and the fetal endocrine system, potentially resulting in differences in appetite control, risk of obesity, and adverse metabolism throughout the lives of offspring. This may perpetuate obesity and adverse cardio-metabolic outcomes across generations, as the daughters of overweight women would be predisposed to enter pregnancy overweight and with adverse metabolic profiles themselves. Concerns about this hypothesis are influencing antenatal care; for example, recommendations related to gestational weight gain and the new criteria for diagnosing gestational diabetes are aimed at reducing future offspring obesity and adverse metabolism [4,5]. However, whether the associations of maternal adiposity and associated traits with offspring outcomes are causal is unknown, and if they are causal, then the mechanisms are unclear [4,6,7].

A recent study using genetic instrumental variables (Mendelian randomisation) found that intrauterine exposure to greater maternal adiposity and fasting glucose results in greater birthweight and ponderal index [6]; however, Mendelian randomisation studies, within siblings analyses and negative control studies do not support a causal intrauterine effect of greater maternal gestational adiposity on later offspring adiposity levels [8,9]. Exposure to greater maternal adiposity and associated metabolic disruption could, nonetheless, result in more adverse metabolic profiles in offspring via intrauterine mechanisms even in the absence of an effect on offspring adiposity, for example, through a direct effect on appetite control of the development of the liver and pancreas.

**Fig 1:**
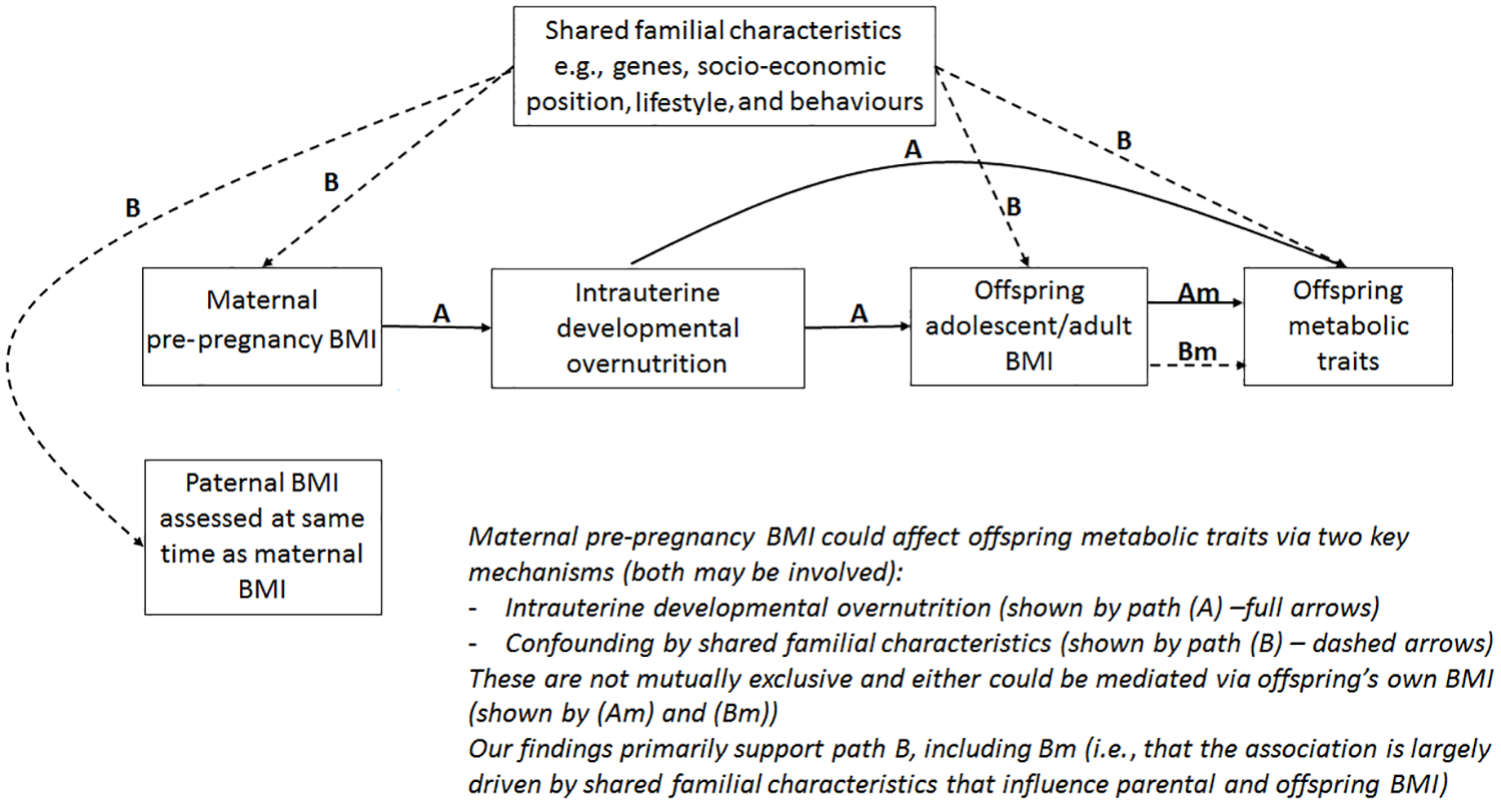
Hypothesised paths between maternal pre-pregnancy BMI and offspring future metabolic traits tested here. Abbreviation: BMI, body mass index.

The aim of this study was to examine associations between maternal pre-pregnancy BMI and multiple offspring serum lipids, lipoproteins, and metabolites in adolescence and adulthood (i.e., when offspring are in their reproductive years), using paternal BMI as a negative control. Our hypotheses are that 2 key paths could explain the association of maternal pre-pregnancy BMI with offspring future metabolic traits: (i) intrauterine developmental overnutrition and (ii) confounding of this potential effect via shared familial genetics, socioeconomic, lifestyle, and behavioural characteristics (Fig 1). It is established that to adjust for all such confounding is impossible in most datasets, and consequently, conventional multivariable approaches are likely to be very biased by residual confounding [10]. Paternal BMI assessed at the same time as maternal BMI is an ideal negative control as it will be influenced by the shared familial characteristics that we are concerned would confound the maternal BMI association, but it could not plausibly result in intrauterine developmental overnutrition [11]. Thus, if maternal associations represent causal intrauterine effects, as opposed to being due to shared familial factors, they should be stronger than paternal associations with the same outcomes [4].

## Methods

### Study populations

All study participants provided written informed consent, and study protocols were approved by the relevant local ethics committees. Specifically, ethical approval for the Avon Longitudinal Study of Parents and Children was obtained from the ALSPAC Ethics and Law Committee and the Local Research Ethics Committees (full details at http://www.bristol.ac.uk/alspac/researchers/data-access/ethics/lrec-approvals/#d.en.164120), and ethical approval for the Northern Finish Birth Cohort 1966 and 1986 studies was obtained from the Ethics Committee of Northern Ostrobotnia Hospital District, Finland.

The parent-specific BMI associations with offspring metabolic profiles were examined in 1 British and 2 Finnish birth cohorts with nuclear magnetic resonance (NMR)–based serum metabolomics: Avon Longitudinal Study of Parents and Children [12,13] (ALSPAC; offspring follow-up at 17 years), Northern Finland Birth Cohort 1966 and 1986 studies [14,15] (NFBC66 and NFBC86; offspring follow-up at 31 and 16 years, respectively). Full details of the cohorts are provided in S1 Text and S1 Fig.

### Parental exposures and covariables

In ALSPAC, parental pre-pregnancy weight, height, education, occupation, and smoking behaviour, and maternal parity were obtained during pregnancy via questionnaires. Paternal weight and height were reported by the mothers (i.e., the recruited pregnant women). Offspring sex was obtained from obstetric records, and parental and offspring ages were calculated from their dates of birth and dates of questionnaires or clinic assessments. Parental occupation was classified into social class groups from I (managerial) to IV (unskilled manual workers). Highest educational qualification for both parents was collapsed into 1 of 5 categories from none/Certificate of Secondary Education (CSE; national school exams at age 16) to university degree.

In NFBC86, parental height, weight, occupation, and smoking status; offspring sex; and maternal parity were collected using questionnaires given to all mothers at their first antenatal clinic visit. As in ALSPAC, paternal weight and height were reported by their partner (the mother). Level of education was obtained from questionnaires in 2001–2002. Parental and offspring ages were derived from their dates of birth and dates of assessments. Parental education was categorised into 8 categories from no occupational education to university degree, and occupation was categorised into 6 categories from entrepreneur to no occupation.

In NFBC66, maternal height, weight, occupation, smoking status, parity, and offspring sex were reported by mothers at the first antenatal clinic visit (16th week of gestation), or in questionnaires administered between the 24th and 28th week of gestation. Offspring age at serum collection was derived from their date of birth and date of attendance at the 1997–1998 follow-up clinic. Maternal age in pregnancy was derived from year of birth and the date of pregnancy questionnaire completion. Education was categorised into 9 categories from none or circulating school to beyond matriculation exam, and occupation was categorised into 5 categories ranging from I (highest social class) to V (no occupation). Information on paternal BMI was not collected in NFBC66; therefore, this cohort’s data were used to test for replication of the maternal-offspring association results only.

In all cohorts, head of household social class was defined as the highest occupation of either parent. For the 1-stage individual participant data (IPD) [16–18] meta-analysis, education and head of household occupational social class categories were harmonised between cohorts (see S1 Text and S1 Table). Individual cohort variables were used in the 2-stage IPD meta-analysis. In ALSPAC, parents-offspring trios where the mother had reported that her partner was not the biological father of the offspring, and those for whom this information was missing, were excluded; this information was not available for the Northern Finland Birth Cohort studies (NFBCs). Differences in fetal growth in multiple pregnancies suggest that intrauterine effects are different for singletons and multiple births [19]: for the purpose of this study we considered only singleton pregnancies. For our analyses, we used data from 5,327 to 5,377 mother-father-offspring trios from ALSPAC and NFBC86, and 4,841 to 4,874 mother-offspring pairs from NFBC66 who had data on parental BMI, offspring metabolite, and covariables.

### Outcomes: Metabolic profiling

A comprehensive profiling of offspring circulating lipids, lipoproteins, and metabolites was done by a high-throughput NMR metabolomics platform, providing a snapshot of offspring serum metabolome at follow-up [20,21]. In ALSPAC, offspring metabolic traits were assessed on fasting (minimum 6 hours) plasma at 2 ages (mean ages of 15.5 and 17.8), and we used data from either of these. As we were interested in lasting effects into reproductive years, we prioritised measures from the older age follow-up and used the earlier measures only for participants who did not have measures at mean age 17.8. Mean age at assessment in the whole cohort after using both time points was 17. Participants of both NFBCs fasted overnight before serum collection on the morning of clinic attendance (8 to 11 AM) at mean age 16 (NFBC86) and 31 (NFBC66) years.

Collectively, the 153 metabolic traits measured by the platform represent a broad molecular signature of systemic metabolism [20,21]. The platform provided simultaneous quantification of lipoprotein lipids and subclasses, FAs and FA compositions, ketone bodies, amino acids, as well as glycolysis and gluconeogenesis-related metabolites in absolute concentration units. This platform has been applied in various large-scale epidemiological and genetic studies [22–25]; the detailed protocol, including information on quality control, has been published elsewhere [21,26] and more information is given in S1 Text.

### Statistical analysis

A draft analysis plan was written by DLSF and DAL in March 2015. This was shared with other cohort analysts and investigators, and a final analysis plan was agreed upon in September 2015 (see S2 Text), with analyses commencing in early 2016. Two changes were made to the analysis plan after analyses had begun. In October 2016, in response to one of the coauthors, it was agreed that we would explore whether parental BMI–offspring metabolic trait associations were attenuated with additional adjustment for offspring BMI. In our original plan, we had agreed to explore offspring BMI–offspring metabolic trait associations but had not planned to adjust parental BMI–offspring metabolic traits for offspring BMI. In May 2017, in response to one reviewer, we constructed a random intercept and slope multilevel model to further assess the agreement between maternal and paternal associations. In our original plan, we proposed to assess this using linear regression only. No further changes to the original plan were made.

Data cleaning and checking were performed separately for each cohort. One-stage and 2-stage IPD [16–18] meta-analyses were performed to assess the associations of maternal pre-pregnancy BMI with offspring metabolic profiles, using paternal BMI as a negative control. The term IPD relates to the data recorded for each participant in a study [16]. Linear regression models were adjusted for parental age, smoking, education, head of household social class, maternal parity, offspring age at blood collection, and sex. Robust standard errors were estimated for all associations and probability values, as some metabolic traits concentrations had skewed distributions.

We conducted 3 sets of IPD meta-analysis that included maternal versus paternal comparisons:

1. A 1-stage IPD meta-analysis restricted to the 2 cohorts with data on both maternal and paternal BMI (ALSPAC and NFBC86) which were our main analyses. In these analyses, offspring metabolic traits were standardised (z-scored) across both cohorts and then regressed on maternal and paternal z-scored BMI (again standardised across both cohorts, separately for mothers and fathers) with adjustment for the harmonised covariables and a binary variable reflecting whether the participant was from ALSPAC or NFBC86. These analyses were conducted on between 5,327 and 5,377 trios (numbers with complete data varied slightly for different metabolic traits). This approach has greater statistical efficiency than a 2-stage IPD meta-analysis, and restricting to the 2 cohorts with both maternal and paternal BMI ensures that exposure-outcome comparisons are possible within all parental-offspring trio data available. It assumes that the 2 cohorts are from the same population to which inferences are being made.
2. A 1-stage IPD meta-analysis, undertaken as described above, but including the maternal BMI–offspring metabolite associations from NFBC66. This sensitivity analysis assessed associations of maternal BMI with offspring metabolic traits in up to 4,874 additional mother-offspring pairs (10,181 to 10,251 in total) from ALSPAC, NFBC86, and NFBC66 and compared these with the same associations of paternal BMI with offspring metabolic traits in ALSPAC and NFBC86 only (*N* = 5,327 to 5,277). This has greater statistical power for the maternal associations and for determining differences between mothers and fathers. It assumes that all 3 cohorts are from the same population to which inference is being made. This includes the assumption that paternal BMI would be similarly associated with offspring metabolic traits in NFBC66 as in the other 2 cohorts if we would have been able to model data on this unmeasured trait.
3. 2-stage IPD meta-analysis in which the regression outputs of standardised offspring metabolic traits with standardised parental BMI were derived separately for each cohort and for maternal and paternal BMI (stage 1) was performed, and then pooled results from each cohort were meta-analysed together using the random effects inverse-variance-weighted method (stage 2). The standardisation of offspring metabolic traits and parental BMI was undertaken within each cohort in this method. We used this method to (a) explore whether our standardisation across cohorts of offspring metabolic traits and parental BMI, and harmonisation of potential confounders in the 1-stage IPD meta-analyses, had notably influenced results; (b) test for heterogeneity in association results between the 3 cohorts (2 for paternal BMI–offspring metabolic traits associations) using the I^2^ statistics [27], which provides a test of replication of our findings and tests our assumption that the cohorts are likely to be from the same population to which we want to make inference; and (c) test, by using a random effects method for pooling results (even if there was little evidence of heterogeneity), whether results were similar if we relaxed the assumption of the cohorts all being from the same underlying population (this approach provides results that are interpreted as the average across studies, assuming that these might reflect different populations).

Magnitudes of maternal and paternal associations were compared by presenting the results one on top of the other so that the extent to which point estimates and their respective confidence intervals differ (or not) can be clearly seen. In addition, the magnitudes of maternal and paternal associations were compared to each other using linear fit and a random intercept and slope multilevel model. The latter was used to overcome the fact that as metabolic traits are correlated, the independence assumption of the linear fit analyses are likely to be violated, which could bias results. We defined clusters according to metabolic trait-classes, which resulted in 27 classes with between 1 and 9 metabolic traits in each class (see S2 Table). This model is composed of a fixed effects part, which is the average association of maternal BMI–offspring metabolic trait point estimates with paternal BMI–offspring metabolic trait point estimates for each metabolic trait (level 1) and the same associations of maternal and paternal point estimates, within their trait classes, allowing the intercepts and slopes of these to all vary from the level 1 averages (associations in these classes are level 2). Conditional ($R_{cond}^{2}$) and marginal ($R_{marg}^{2}$) goodness of linear fit [28] were computed. These represent the agreement between maternal and paternal point estimates when clustering within classes is taken into account ($R_{marg}^{2}$), plus when variance explained by the individual classes is included ($R_{cond}^{2}$). One-stage IPD associations were compared to the corresponding 2-stage IPD for maternal and paternal BMI separately, also by examining their linear fit. The differences between maternal and paternal associations (in all 3 approaches) were calculated from the bootstrap replicate distribution (1,000 replications). To this end, we sampled with replacement parental-offspring trios so that the correlation structure of the original dataset was maintained. Beta-estimates and standard errors were empirically calculated from the mean and SD of the bootstrap distribution, respectively. All *P* values were calculated using bootstrap means and standard errors and compared to a z-distribution.

To establish a threshold that takes into account multiple testing and the correlation structure of the metabolic traits data, principal components analysis (PCA) was carried out on the z-scored metabolic traits data [29]. The rationale for defining the number of independent tests via PCA has been discussed previously [25,29,30], and more information is available in S1 Text. The first 17 principal components (PCs) explained 95% of the metabolic traits data variance across the 3 cohorts; this number is a proxy of the number of independent tests being performed. Therefore, the threshold of *P* < 0.05 becomes *P* < 0.003 (i.e., α ÷ 17 where α = 0.05) when multiple testing is considered for assessing associations with the 153 metabolic traits.

We performed 2 additional analyses, using the 1-stage IPD meta-analysis only, that aimed to explore whether any maternal (or paternal) pre-pregnancy BMI associations with offspring metabolic traits were mediated by the relationship of the parental BMI with offspring BMI. First, we examined the associations of offspring BMI (assessed at the same time as blood collection) with their metabolic traits. Second, we repeated the meta-analysis described above, in point 1, with additional adjustment for offspring BMI. Both analyses were undertaken in *N* = 5,266 to 5,316 trios from ALSPAC and NFBC86 cohorts.

Statistical analyses were conducted using R version 3.0.1 (R Foundation for Statistical Computing, Vienna, Austria) and Stata version 14.1 (Stata Inc., TX, USA).

## Results

Characteristics of the 3 study populations are shown in S1 Table, and the flowchart is shown in S1 Fig. The percentage of overweight or obese mothers was 20%, 15%, and 23% in ALSPAC, NFBC86, and NFBC66, respectively, and for fathers was 47% and 32% in ALSPAC and NFBC86, respectively. The highest percentage of overweight or obesity in offspring, at follow-up, in adolescent or adulthood was seen in NFBC66 with 40% (mean age 31 years), followed by ALSPAC with 21% (mean age 17 years), and NFBC86 with 11% (mean age 16 years).

**Fig 2:**
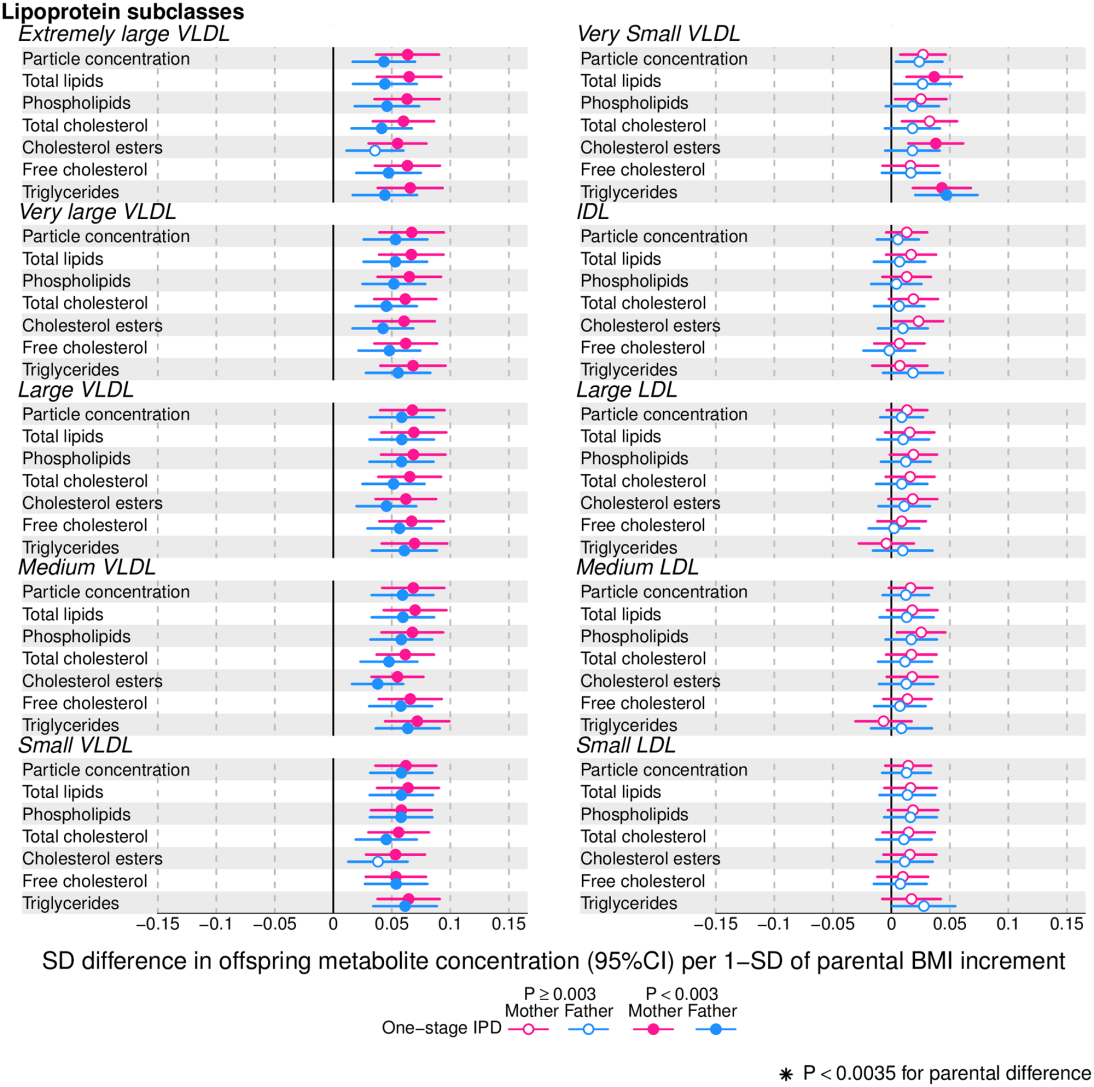
One-stage individual participant data meta-analysis. Offspring lipoprotein and lipid differences in means in SD units per 1-SD higher maternal (pink) or paternal (blue) body mass index (BMI), meta-analysed across Avon Longitudinal Study of Parents and Children (ALSPAC) and Northern Finland Birth Cohort of 1986 (NFBC86) cohorts. Associations were adjusted for parental age, smoking, education, head of household social class, maternal parity, offspring age at blood collection, sex, and cohort membership. Results are shown in SD-scaled concentration units of outcome; differences in absolute concentration units are listed in S3 Table. Error bars = 95% confidence intervals (CI). Abbreviations: IDL, intermediate-density lipoprotein; LDL, low-density lipoprotein; VLDL, very-low-density lipoprotein.

**Fig 3:**
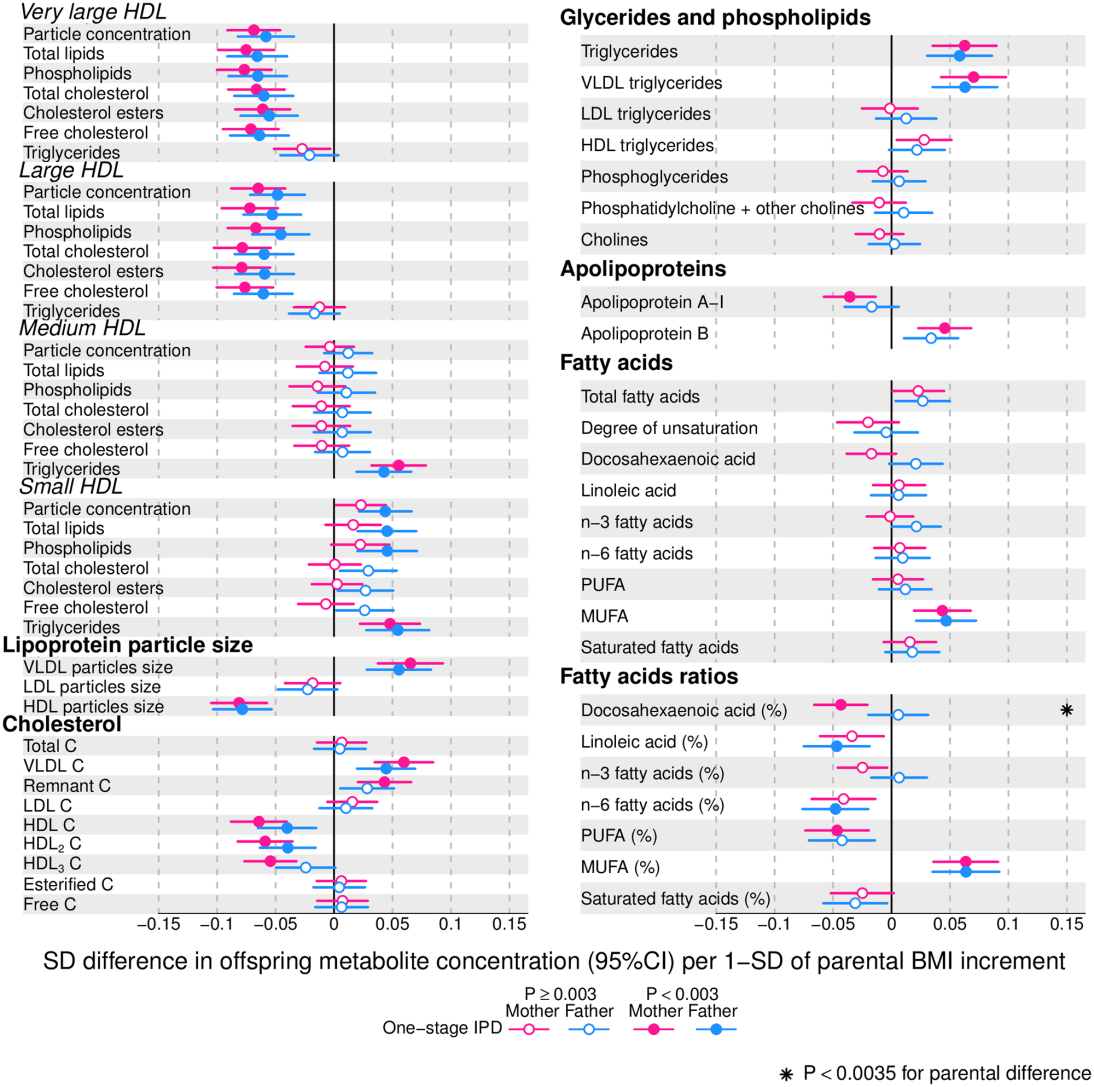
One-stage individual participant data meta-analysis. Offspring lipoprotein and lipid differences in means in SD units per 1-SD higher maternal (pink) or paternal (blue) body mass index (BMI), meta-analysed across Avon Longitudinal Study of Parents and Children (ALSPAC) and Northern Finland Birth Cohort of 1986 (NFBC86) cohorts. Associations were adjusted for parental age, smoking, education, head of household social class, maternal parity, offspring age at blood collection, sex, and cohort membership. Results are shown in SD-scaled concentration units of outcome; differences in absolute concentration units are listed in S3 Table. Error bars = 95% confidence intervals (CI). Abbreviations: C, cholesterol; HDL, high-density lipoprotein; MUFA, monounsaturated fatty acids; PUFA, polyunsaturated fatty acids.

**Fig 4:**
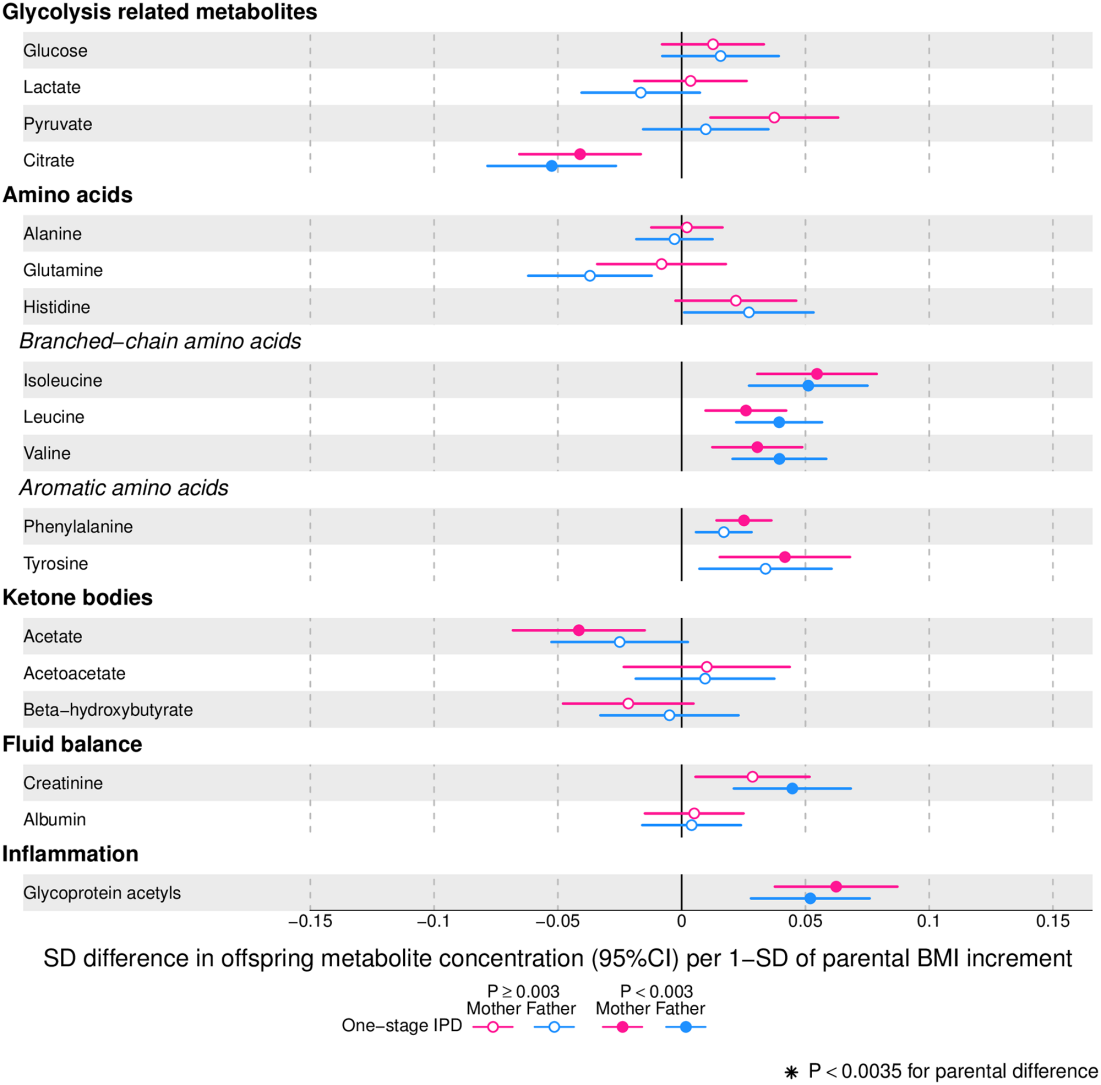
One-stage individual participant data meta-analysis. Offspring metabolite differences in means in SD units per 1-SD higher maternal (pink) or paternal (blue) body mass index (BMI), meta-analysed across Avon Longitudinal Study of Parents and Children (ALSPAC) and Northern Finland Birth Cohort of 1986 (NFBC86) cohorts. Associations were adjusted for parental age, smoking, education, head of household social class, maternal parity, offspring age at blood collection, sex, and cohort membership. Results are shown in SD-scaled concentration units of outcome; differences in absolute concentration units are listed in S3 Table. Error bars = 95% confidence intervals (CI).

Figs 2–4 show associations of parental BMI with the 153 offspring metabolic measures, each expressed as a difference in means in SD units for a 1-SD greater parental BMI, from a 1-stage IPD meta-analysis restricted to the 2 cohorts with complete trio data (*N* = 5,327 to 5,377 trios). S3 Table shows the same associations expressed as magnitudes in absolute concentration units (e.g., mmol/l per 1-SD difference in parental BMI). Both maternal and paternal pre-pregnancy BMI were associated with a large proportion of the offspring metabolic traits (maternal BMI associated with 49% and paternal BMI with 44% of offspring metabolic traits at *P* < 0.003). The associations were mostly in the same direction for both parents and were in the direction of more adverse cardio-metabolic risk in offspring with higher parental BMI in pre-pregnancy.

Amongst lipoprotein lipids, the most marked positive associations were observed in 5 very-low-density lipoprotein (VLDL) subclasses (all VLDL except the very small subclass). There were no robust associations of parental BMI with offspring intermediate-density lipoprotein (IDL) and low-density lipoprotein (LDL) subclasses. Strong inverse associations were observed for the very large and large high-density lipoprotein (HDL) subclasses with the exception of structural triglycerides that exhibited no clear associations. Lipoprotein particle sizes followed the same pattern of associations as those observed with lipoprotein subclasses. Parental BMI was strongly positively associated with offspring VLDL cholesterol, VLDL triglycerides, triglycerides, and remnant cholesterol, with the same direction and comparable magnitudes to the first 5 VLDL lipoprotein subclasses. Conversely, HDL, HDL_2_, and HDL_3_ cholesterol sub-fractions showed inverse associations, with magnitudes similar to the very large and large HDL lipoprotein subclasses. Associations of parental BMI with apolipoprotein A-I and B were in opposite directions, in agreement with the association pattern for lipoprotein subclasses.

Parental BMI was strongly positively associated with offspring branched-chain amino acids and maternal BMI with aromatic amino acids.

Mono-unsaturated fatty acids (MUFA) was the only FA clearly associated with parental BMI when assessed alone or as a proportion of total FAs (MUFA-to-total FA ratio). Several of the other FAs showed associations with parental BMI, but only as proportions of total FAs. Poly-unsaturated (PUFA) to total FAs (PUFA-to-total FA ratio), ratio of linoleic acid (LA) to total FAs (LA-to-total FA ratio), and omega 6 to total FA ratio were all inversely associated with parental BMI.

Parental BMI was not clearly associated with any of the glycolysis-related metabolites, except citrate where there was an inverse association. Parental BMI was strongly positively associated with offspring glycoprotein acetyls, an inflammatory marker.

**Fig 5:**
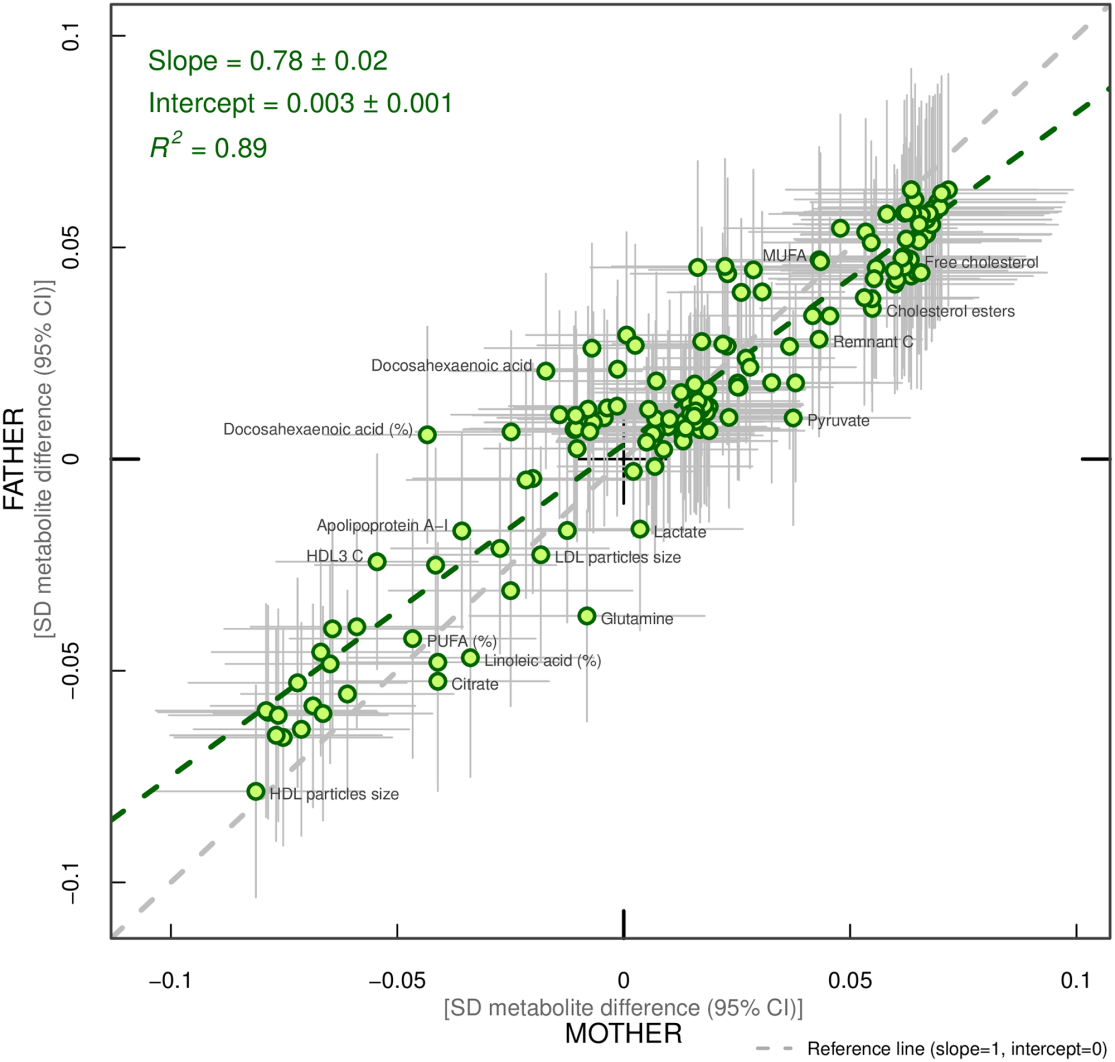
Linear fit between paternal and maternal models (green dashed line). Each green dot represents a metabolic trait and the positions of the dots are determined by difference in mean offspring metabolite (in SD units) for each increase of 1-SD maternal body mass index (BMI) (x-axis) and difference in mean offspring metabolite (in SD units) for each increase in 1-SD paternal BMI (y-axis). The horizontal grey lines on each dot denote the confidence intervals (CI) for maternal associations and the vertical grey lines indicate the CI for paternal estimates. A linear fit of the overall correspondence summarises the similarity in magnitude between maternal and paternal associations (green dashed line). A slope of 1 with an intercept of 0 (dashed grey line), with all green dots sitting on that line (R^2^ = 1), would indicate that maternal and paternal estimates had the same magnitude and direction. R^2^ indicates goodness of linear fit and as such is a measure of the consistency between maternal and paternal associations. Results are shown in SD-scaled concentration units of outcome, difference in absolute concentration units are listed in S3 Table. Abbreviations: C, cholesterol; HDL, high-density lipoprotein; LDL, low-density lipoprotein; MUFA, monounsaturated fatty acids; PUFA, polyunsaturated fatty acids.

Overall, associations across offspring metabolic traits were similar for maternal and paternal BMI, as can be seen in Figs 2–4, and also by the similarity of results when directly modelling the 2 sets of point estimates by both linear fit and multi-level models (Fig 5—linear fit: R^2^ = 0.89 and slope = 0.78 ± 0.02; S2 Fig—multi-level model: $R_{cond}^{2}$ = 0.94 and $R_{marg}^{2}$ = 0.87). Furthermore, for all except 1 of the metabolic traits, there was no strong statistical evidence that associations differed between maternal and paternal BMI (*P*_boot_ > 0.003). Docosahexaenoic acid (DHA)-to-total FA ratio was the only metabolic trait that showed some suggestion of difference in association between maternal and paternal BMI (mean difference in percentage per 1-SD BMI: β_mother_ = −0.01, 95% CI_mother_ = −0.02 to −0.01, *P*_mother_ = 0.0002 versus β_father_ = 0.002, 95% CI_father_ = −0.01 to 0.01, *P*_father_ = 0.66; *P*_boot_ for difference between the 2 associations = 0.0034; Figs 2–4). However, the difference between maternal and paternal association appeared to be driven primarily by results in ALSPAC as the difference was not seen in NFBC86 or the 2-stage IPD meta-analysis (S3 Fig).

When the 1-stage analyses were repeated to compare maternal BMI–offspring metabolic trait associations in all 3 cohorts (*N* = 10,181 to 10,251) to paternal BMI–offspring metabolic trait associations in the 2 cohorts with paternal BMI (*N* = 5,327 to 5,377), the maternal associations were essentially the same as those described above; no statistical evidence for stronger maternal associations emerged (S4 Fig).

In the 2-stage meta-analysis, the associations were highly consistent across the 3 cohorts, with just 5% and 3% of associations having I^2^ statistic ≥75% for maternal and paternal BMI, respectively, suggesting that the majority of our findings replicate across the 3 independent cohorts (S3 Fig and S4 Table). A comparison of the results from 1- and 2-stage IPD meta-analyses showed very similar patterns of association for both maternal and paternal BMI (R^2^ = 0.96 and slope = 0.81 ± 0.01; R^2^ = 0.99 and slope = 1 ± 0.01, respectively, for maternal and paternal BMI; S5 Fig). The magnitudes of associations in absolute concentration units for individual cohorts, as well as for the 2-stage IPD meta-analysis, are shown in S4–S6 Tables.

Cross-sectional associations of offspring BMI with their metabolic traits had very similar patterns (in terms of direction and which metabolic traits had strongest and weakest associations) to those seen for parental BMI but were considerably stronger in magnitude (S6 Fig). When adjusting parental BMI–offspring metabolite associations for offspring BMI, most attenuated markedly, such that many point estimates were in the opposite directions and the vast majority were consistent with the null following this adjustment (S7 Fig).

## Discussion

We report, to the best of our knowledge, the first study that investigates the potential influence of maternal pre-pregnancy BMI on adult offspring serum metabolome to determine whether intrauterine mechanisms related to developmental overnutrition result in metabolic disruption in adults when they are in their reproductive years. We found similar associations of both maternal and paternal BMI with offspring systemic metabolism 16–17 years later and 31 years later (the latter assessed with maternal BMI only), suggesting that shared familial genetic, socioeconomic, and lifestyle characteristics rather than an intrauterine programming effect explain the associations of maternal pre-pregnancy BMI with offspring metabolic measures (Figs 2–4).

Associations of greater parental BMI with offspring lipids were in the directions of an adverse cardio-metabolic profile, with positive associations with VLDL-lipoproteins, VLDL-cholesterol (C), VLDL-triglycerides, VLDL-diameter, branched/aromatic amino acids, glycoprotein acetyls, and triglycerides, and inverse associations with HDL-lipoprotein, HDL-diameter, HDL-C, HDL_2_-C and HDL_3_-C. Associations were also seen with FA ratios and some amino acids. In further analyses, we demonstrated that in addition to similar associations between paternal BMI with offspring metabolic traits (to those seen for maternal BMI), the offspring BMI was associated in a similar pattern (but with stronger magnitudes) to their metabolic traits, further supporting the notion that shared familial characteristics explain these associations. Lastly, we found that parental associations with metabolic traits attenuated markedly with adjustment for offspring BMI, suggesting that shared familial characteristics drive associations of maternal pre-pregnancy BMI and paternal BMI with their offspring BMI, which in turn results in metabolic disruption (Fig 1).

Previous studies have compared associations of maternal and/or paternal BMI, measured pre-pregnancy, with offspring adiposity (BMI [3,31–33], overall adiposity [31], central adiposity [31–33]), with fewer also examining association with offspring lipids (HDL-C [31–33], LDL-C [31,32], total cholesterol [31,32], triglycerides [31]), blood pressure [31–33], glucose [33], and insulin [31]. Our study is generally larger than these previous studies, examines a detailed metabolic profile with 153 metabolic traits (lipids, lipoproteins, and metabolites), and assesses outcomes in offspring at older ages. Where it is possible to make comparisons, our findings are consistent with the reported lack of association between maternal BMI and offspring total cholesterol [31,32], LDL-C [31], and glucose [33], and inverse association with HDL-C [31,33] (previous studies *N* = 70 to 4,871; offspring age range: 4–8 years). Our results also contrast reports from some of the same studies that found no association of maternal BMI with offspring triglycerides [31,33] or HDL-C [32]. These differences might reflect differences in the age of offspring outcome and/or that we are able to assess associations with more refined outcomes (e.g., HDL-C subfractions). The only previous study that we were able to identify that compared maternal to paternal BMI associations with any outcomes similar to ours and with a similar size (*N* = 4,871) to our main analysis approach reported similar associations of maternal BMI with total cholesterol, LDL-C, and HDL-C to those seen for paternal BMI [31], as in our study, though that study found no association of either parental BMI with offspring triglycerides. Associations between offspring BMI and their serum metabolomic profile are similar to the ones reported by Wurtz et al. [34].

Our study has several strengths. It has a large sample size, included replication testing across 3 different birth cohorts from 2 different countries, and included very detailed offspring blood metabolome measurements. The consistency of associations across 3 independent studies using different analytical approaches suggests that our results are unlikely to be due to chance. Furthermore, we used a negative-control approach (with paternal BMI) to explore causal inference. Using paternal BMI as a negative control makes it possible to disentangle the extent to which maternal BMI–offspring metabolite associations are due to casual intrauterine effects or confounding by familial factors (i.e., genetic and/or shared lifestyle traits within families) [4,35]. As we would expect from previous studies [36–39], maternal and paternal BMI are weakly correlated in our cohorts (0.17 in ALSPAC and 0.2 in NFBC86). This correlation is likely to be driven by shared genetic, socioeconomic, and lifestyle characteristics [38,39], which are the very characteristics that we were concerned would confound conventional (without negative control) multivariable regression analyses of maternal BMI with offspring metabolic traits. Thus, paternal BMI is an ideal negative control, as it fulfils the criteria of having the same, or very similar, confounding structure as the main risk factor of interest (maternal BMI), but it is implausible that paternal BMI would affect offspring future metabolism through the intrauterine overfeeding mechanisms that are hypothesised and that we have investigated [4]. The fact that maternal and paternal associations are very similar highlights the importance of the negative control approach and the likelihood that had we only presented maternal multivariable adjusted results, incorrect conclusions about a possible intrauterine effect may have been made. We performed a 1-stage IPD meta-analysis, which is the gold standard of meta-analysis [40], for our main analytical approach, but also tested the assumptions of this approach, and the effect of harmonisation across studies, by comparing the results to a 2-stage IPD, and found high levels of consistency between the 2 approaches.

Limitations of our study include the use of parental BMI as a measure of adiposity; different body composition characteristics, such as fat distribution or fat-to-lean body mass ratio, may show differential associations with offspring serum metabolome. Moreover, maternal BMI was self-reported, and paternal BMI was reported by their partners (i.e., the pregnant mothers), which might have led to misclassification of parental BMI. Another limitation is that inferences cannot be drawn about the role of circulating maternal fetal fuels (i.e., glucose, lipids, FAs, and amino acids) on later offspring metabolic profile, as these were not measured during pregnancy in our cohorts. It is possible that non-paternity for some of the fathers has affected our results (for the NFBCs we had no information on this possibility). However, any impact of non-paternity would be likely to selectively reduce paternal BMI associations, since there would be no shared genetic effects on the association of paternal BMI with offspring metabolic traits, and family lifestyle and socioeconomic associations may be weaker between non-biological parents and offspring. This would thus tend to enhance maternal-paternal differences, whereas we see similar associations between parents. Despite adjusting for several potential confounders, residual confounding may still explain the associations that we have observed between parental BMI and offspring. For example, we were unable to adjust for parental physical activity or dietary intake. These confounders are likely to be similar for maternal and paternal BMI and so would be unlikely to bias our inference about specific maternal effects on offspring metabolism (potentially due to intrauterine programming). Whilst we have a large sample size, we may lack power to detect small differences between maternal and paternal BMI associations with offspring metabolic traits, especially as the human circulating metabolome is a tightly controlled homeostatic system, and even small differences between some metabolic traits might have important clinical impact. However, we think this is unlikely for many of the traits we have examined. First, Figs 2–4 show that, for the majority, associations are precisely estimated (they have narrow CIs), and it can be clearly visualised that there is little difference in magnitude between maternal and paternal associations. Second, we have shown high levels of consistency of maternal and paternal associations as demonstrated by their linear fit (Fig 5) and multilevel model (S2 Fig), and by tests of heterogeneity that show for all but 1 of the metabolic traits there is no strong statistical support for differences. Lastly, at the request of the academic editor and a reviewer, we have undertaken a post-hoc power calculation. For our main analyses, we compared associations in 5,327 to 5,377 mother-father-offspring trios; our power calculation is based on a sample size of 5,300. At our multiple-testing adjusted alpha level of 0.003 and with 80% power, we would be able to detect a minimum difference between the maternal BMI–offspring metabolic trait and paternal BMI–offspring metabolic trait of 0.07-SD per 1-SD BMI, and with 90% power of 0.08-SD across all metabolic traits examined. We have assessed BMI as a continuous variable to explore the hypothesis that each incrementally greater maternal BMI overfeeds the developing infant in utero in a dose-response way, and our results suggest that if that is the case it has no long-term effect on offspring metabolism. However, we cannot exclude a threshold effect—i.e., maternal obesity (or extreme obesity)—having a long-term effect via intrauterine mechanisms. Furthermore, our results may not necessarily generalise to other non-European populations.

In conclusion, the similarity of association between pre-pregnancy maternal BMI and paternal BMI with offspring metabolic profiles suggests that maternal BMI associations are likely to be largely due to shared genetic or familial lifestyle confounding rather than intrauterine mechanisms. Further replication of these findings in other larger studies, including with measured fat distribution in mothers and fathers at the time of the mother’s pregnancy, would be valuable, though we are not aware of any studies with relevant data currently to be able to do that.

## Supporting information

S1 Fig Overview of the study design, cohorts, and statistical analyses. IPD = individual participant data; analysis 1 is our main analysis.
(PDF)

S2 Fig Multilevel linear fit between paternal and maternal models (random intercept and slope). (PDF)

S3 Fig Two-stage IPD meta-analysis and individual cohort associations: offspring lipoprotein, lipids, and metabolite differences per 1-SD higher maternal (pink) or paternal (blue) BMI. (PDF)

S4 Fig One-stage IPD meta-analysis: offspring lipoprotein, lipids, and metabolite differences in means in SD units per 1-SD higher maternal (pink) or paternal (blue) BMI, meta-analysed across ALSPAC, NFBC86, and NFBC66 cohorts. (PDF)

S5 Fig Linear fit between 2- and 1-stage IPD meta-analysis for mother (left panel; pink dashed line) and father (right panel; blue dashed line) models. (PDF)

S6 Fig One-stage IPD meta-analysis: offspring lipoprotein, lipids, and metabolite differences in means in SD units per 1-SD higher maternal (pink), paternal (blue), or offspring (green) BMI, meta-analysed across ALSPAC and NFBC86 cohorts. (PDF)

S7 Fig One-stage IPD meta-analysis: offspring lipoprotein, lipids, and metabolite differences in means in SD units per 1-SD higher maternal (pink) or paternal (blue) BMI, meta-analysed across ALSPAC and NFBC86 cohorts, with and without further adjustment for offspring BMI. (PDF)

S1 Table Characteristics of the 3 study populations. (PDF)

S2 Table Characteristics of the metabolic traits classes. (PDF)

S3 Table One-stage IPD meta-analysis: offspring lipoprotein, lipid, and metabolite absolute concentration differences per 1-SD higher parental BMI. (PDF)

S4 Table Two-stage IPD meta-analysis: offspring lipoprotein, lipid, and metabolite absolute concentration differences per 1-SD higher parental BMI. (PDF)

S5 Table ALSPAC: offspring lipoprotein, lipid, and metabolite absolute concentration differences per 1-SD higher parental BMI. (PDF)

S6 Table NFBC66 and NFBC86: offspring lipoprotein, lipid, and metabolite absolute concentration differences per 1-SD higher parental BMI. (PDF)

S1 Text Supplemental methods. (PDF)

S2 Text Statistical analysis plan. (PDF)

S3 Text STROBE Checklist. (PDF)

## Abbreviations

ALSPAC: Avon Longitudinal Study of Parents and Children
BMI: body mass index
C: cholesterol
DHA: docosahexaenoic acid
FA: fatty acid
HDL: high-density lipoprotein
IDL: intermediate-density lipoprotein
IPD: individual participant data
LA: linoleic acid
LDL: low-density lipoprotein
MUFA: mono-unsaturated fatty acids
NFBCs: Northern Finland Birth Cohort studies
NFBC66: Northern Finland Birth Cohort of 1966
NFBC86: Northern Finland Birth Cohort of 1986
NMR: nuclear magnetic resonance
PC: principal component
PCA: principal component analysis
PUFA: polyunsaturated fatty acids
VLDL: very-low-density lipoprotein
