# Supplementary material for: Association of pre-pregnancy body mass index with offspring metabolic profile: Analyses of 3 European prospective birth cohorts
Source: PLoS Med. 2017 Aug 22;14(8):e1002376. doi: 10.1371/journal.pmed.1002376 (PMC5568725; doi:10.1371/journal.pmed.1002376)

S1 Fig. Overview of the study design, cohorts and statistical analyses. IPD= individual participant data; analysis 1 is our main analysis.

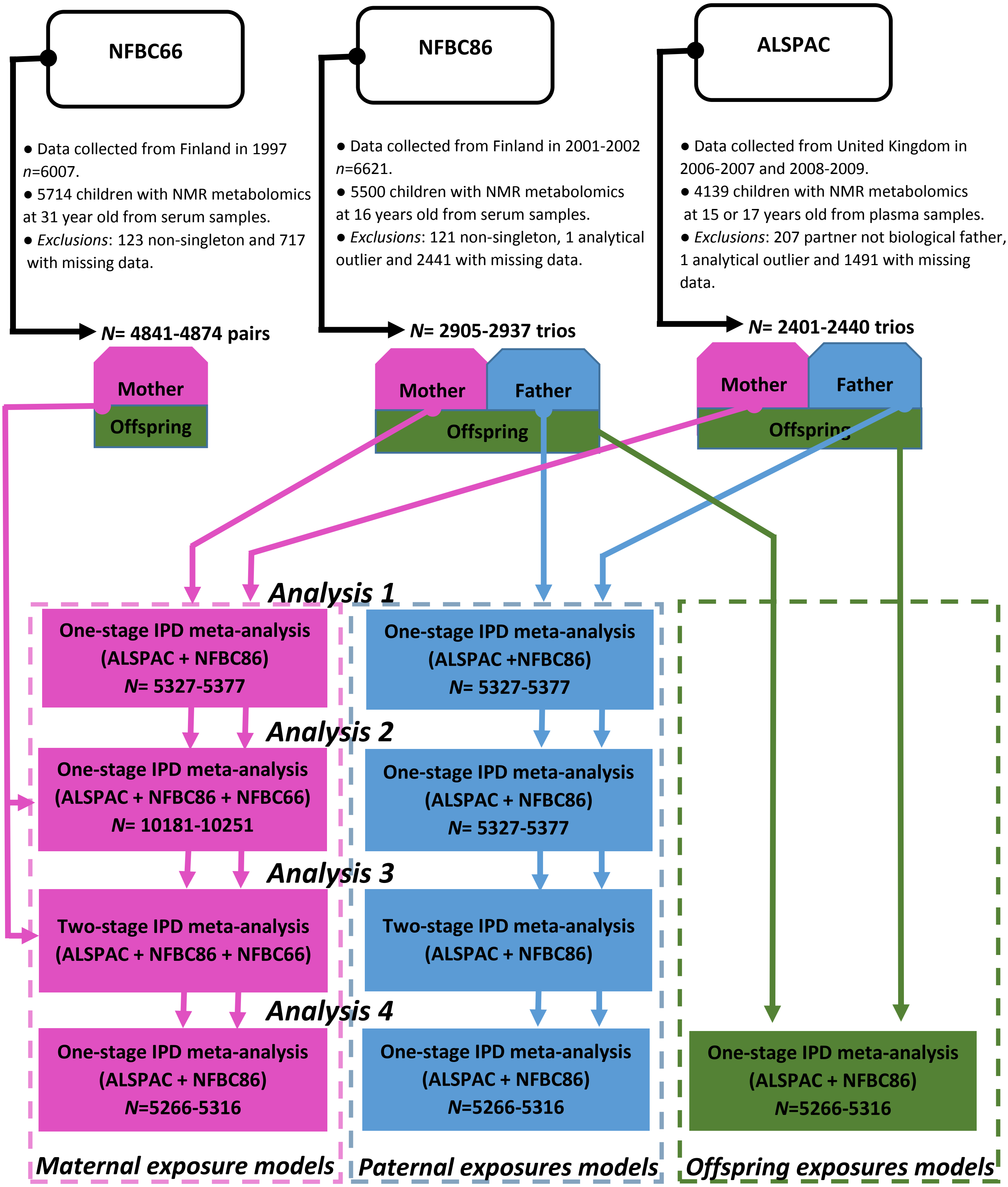

Supplement: S1 Fig — IPD = individual participant data; analysis 1 is our main analysis. (PDF) [file pmed.1002376.s001.pdf]
