# Supplementary material for: Association of pre-pregnancy body mass index with offspring metabolic profile: Analyses of 3 European prospective birth cohorts
Source: PLoS Med. 2017 Aug 22;14(8):e1002376. doi: 10.1371/journal.pmed.1002376 (PMC5568725; doi:10.1371/journal.pmed.1002376)
Supplement: S2 Fig — (PDF) [file pmed.1002376.s002.pdf]

S2 Fig. *Multilevel linear fit between paternal and maternal models (random intercept and slope).*

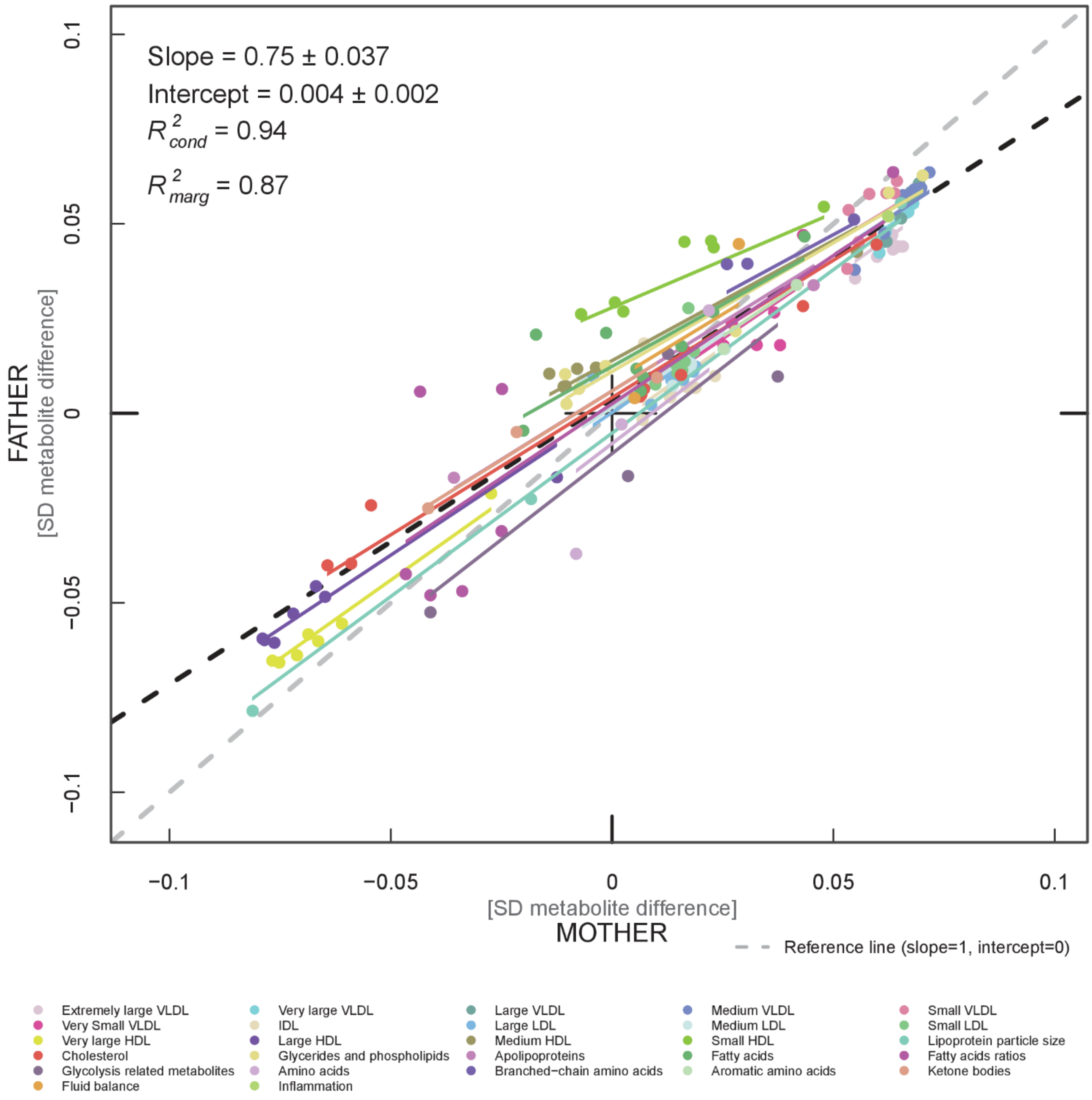

Multilevel regression model: Each dot represents a metabolic trait and the positions of the dots are determined by difference in mean offspring metabolite (in SD units) for each increase of 1SD maternal BMI (x-axis) and difference in mean offspring metabolite (in SD units) for each increase in 1SD paternal BMI (y-axis). Regression lines for random intercept and slope model for overall metabolic traits (dashed black line) and individual metabolic classes (coloured lines). Metabolic traits and metabolic classes lines are colour coded according to their class membership, there are 27 classes. A slope of 1 with an intercept of 0 (dashed grey line), with all dots sitting on that line would indicate that maternal and paternal estimates had the same magnitude and direction. R^2_{marg} (marginal) and R^2_{cond} (conditional) indicates goodness of linear fit. These represent the agreement between maternal and paternal point estimates when clustering within classes are taken into account (R^2_{marg}) and, plus when variance explained by the individual classes is included (R^2_{cond}).
