# Supplementary material for: Association of pre-pregnancy body mass index with offspring metabolic profile: Analyses of 3 European prospective birth cohorts
Source: PLoS Med. 2017 Aug 22;14(8):e1002376. doi: 10.1371/journal.pmed.1002376 (PMC5568725; doi:10.1371/journal.pmed.1002376)
Supplement: S3 Fig — (PDF) [file pmed.1002376.s003.pdf]

S3 Fig. *Two-stage IPD meta-analysis and individual cohort associations: offspring lipoprotein, lipids and metabolite differences per 1-SD higher maternal (pink) or paternal (blue) BMI.*

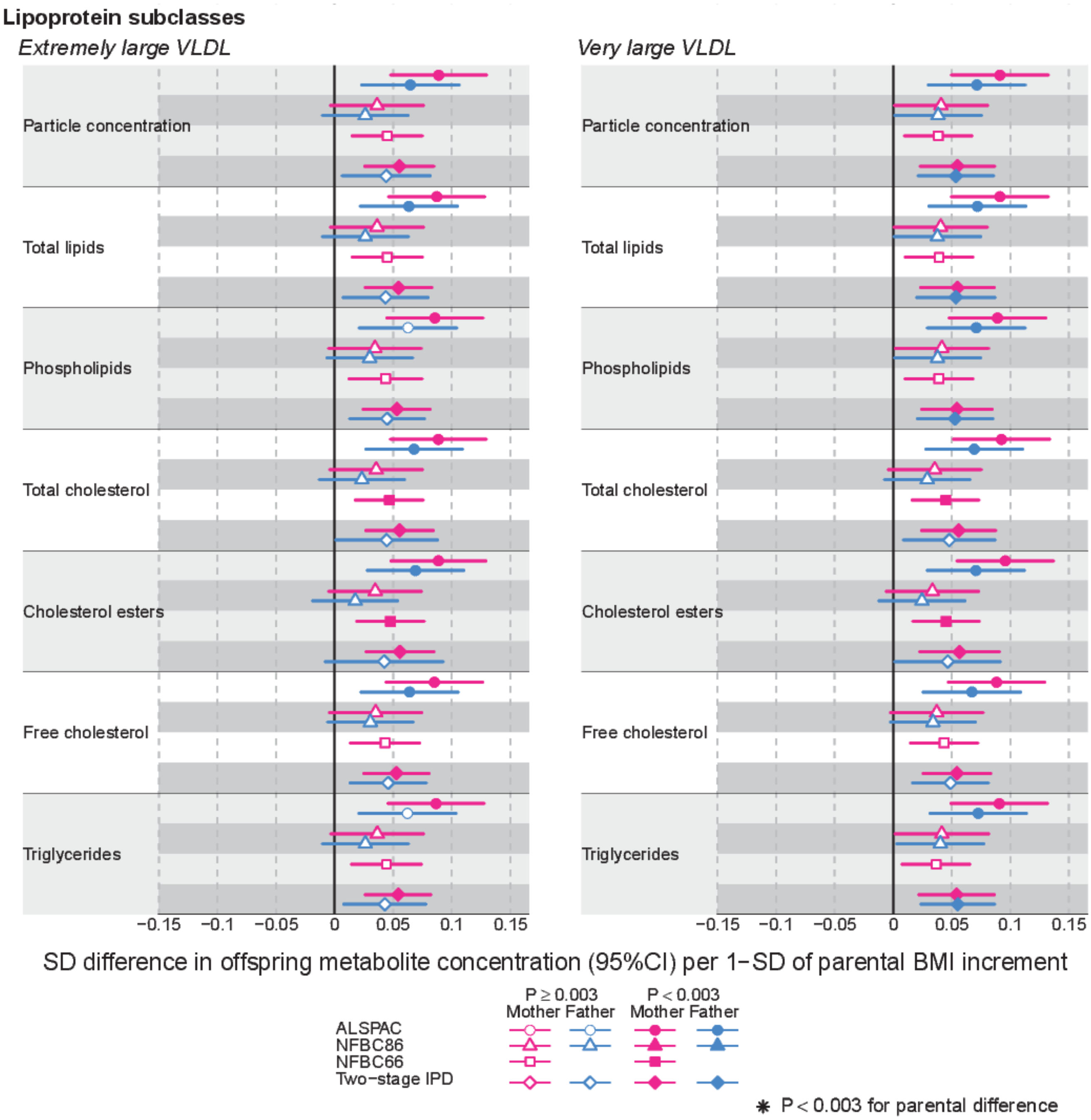

S3 Fig *continued*.

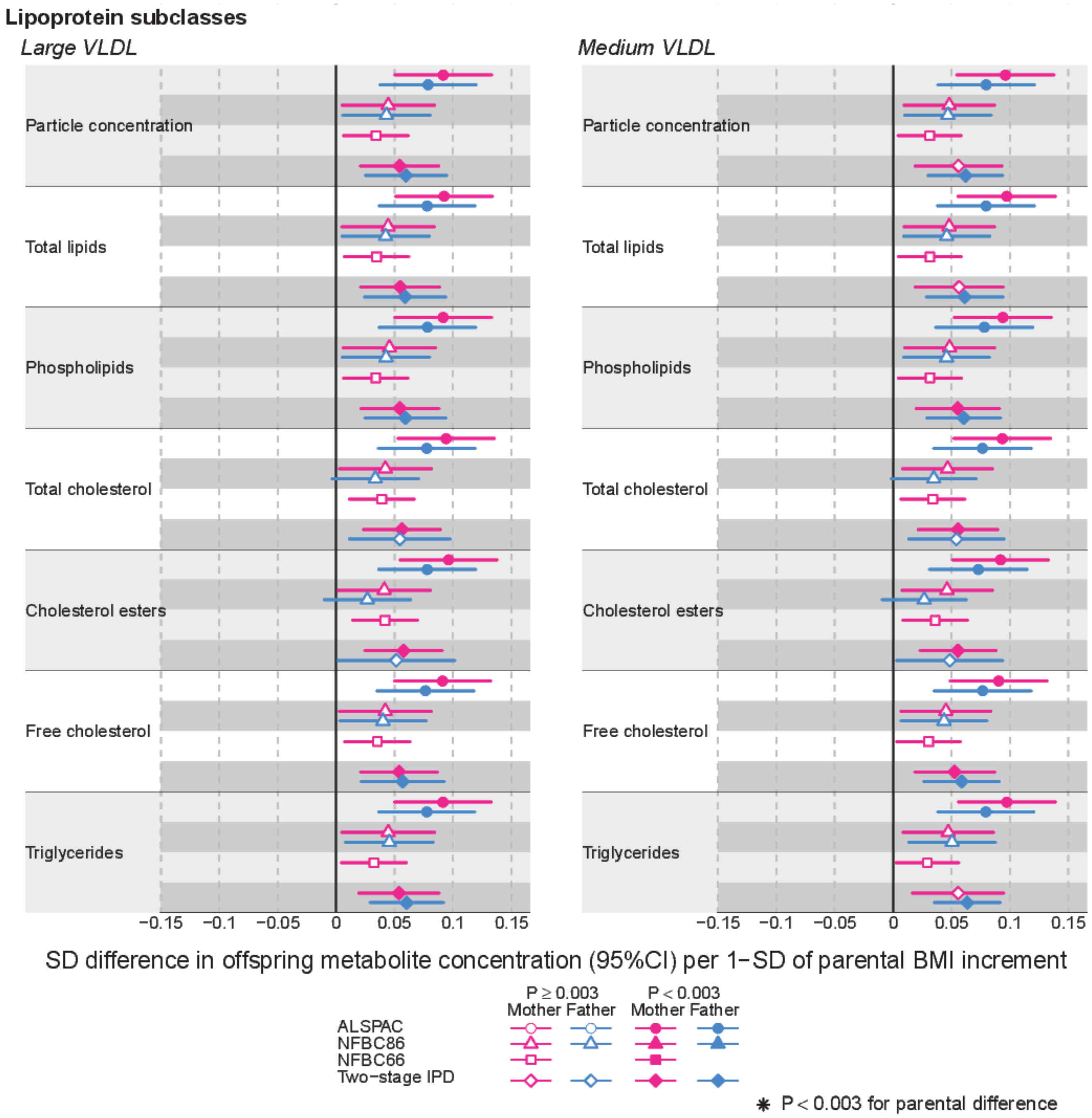

S3 Fig *continued*.

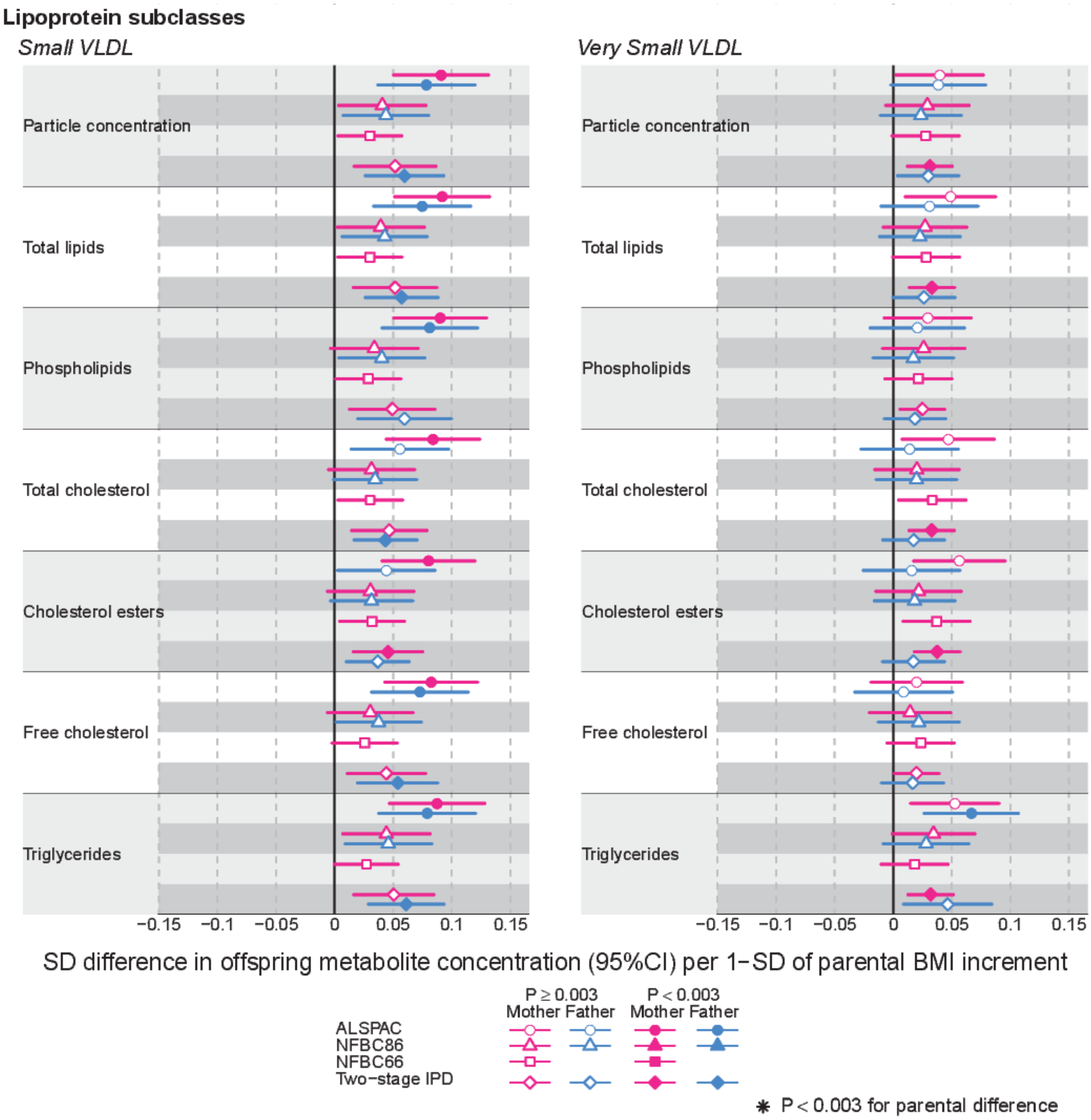

S3 Fig *continued*.

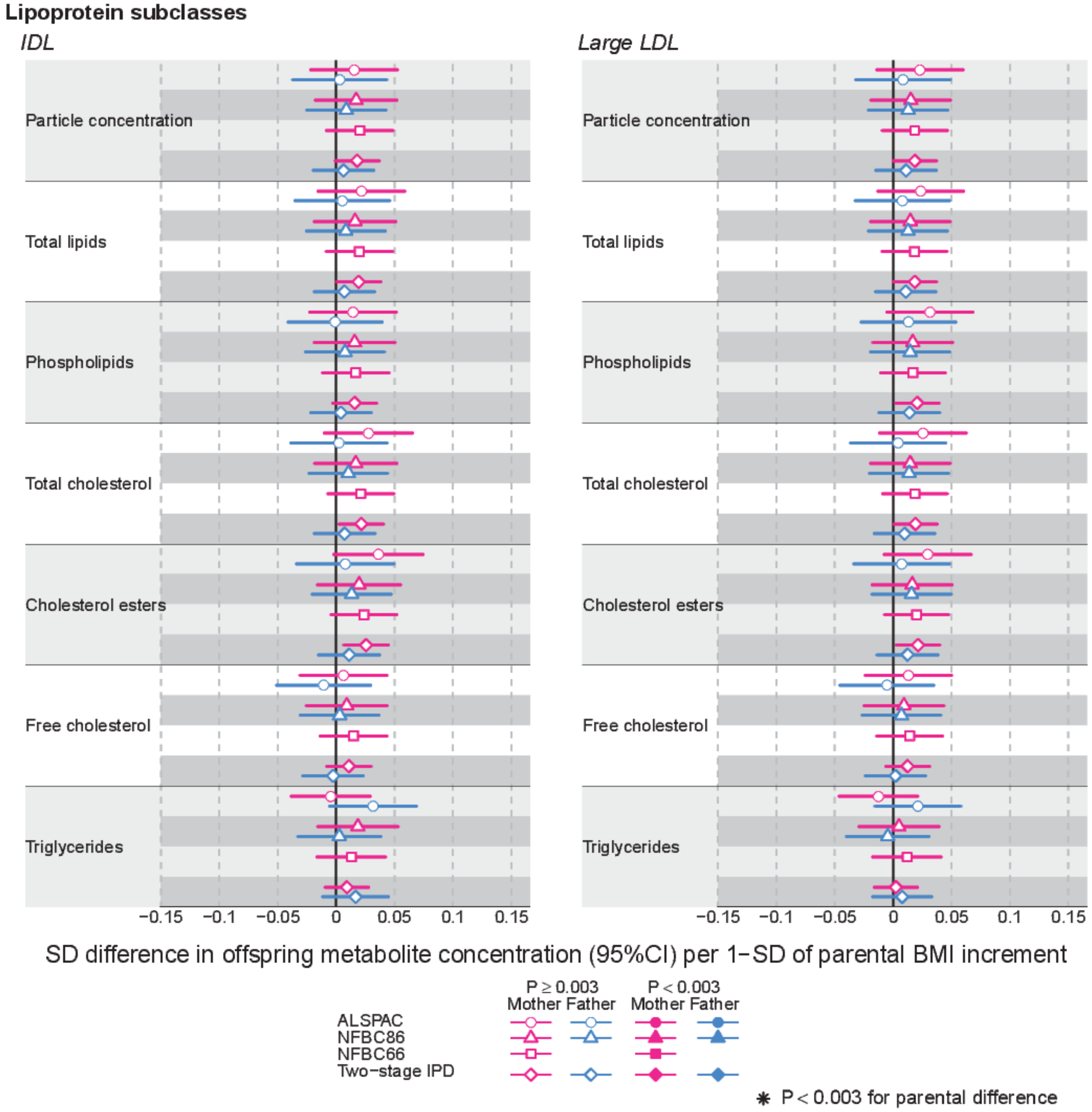

S3 Fig *continued*.

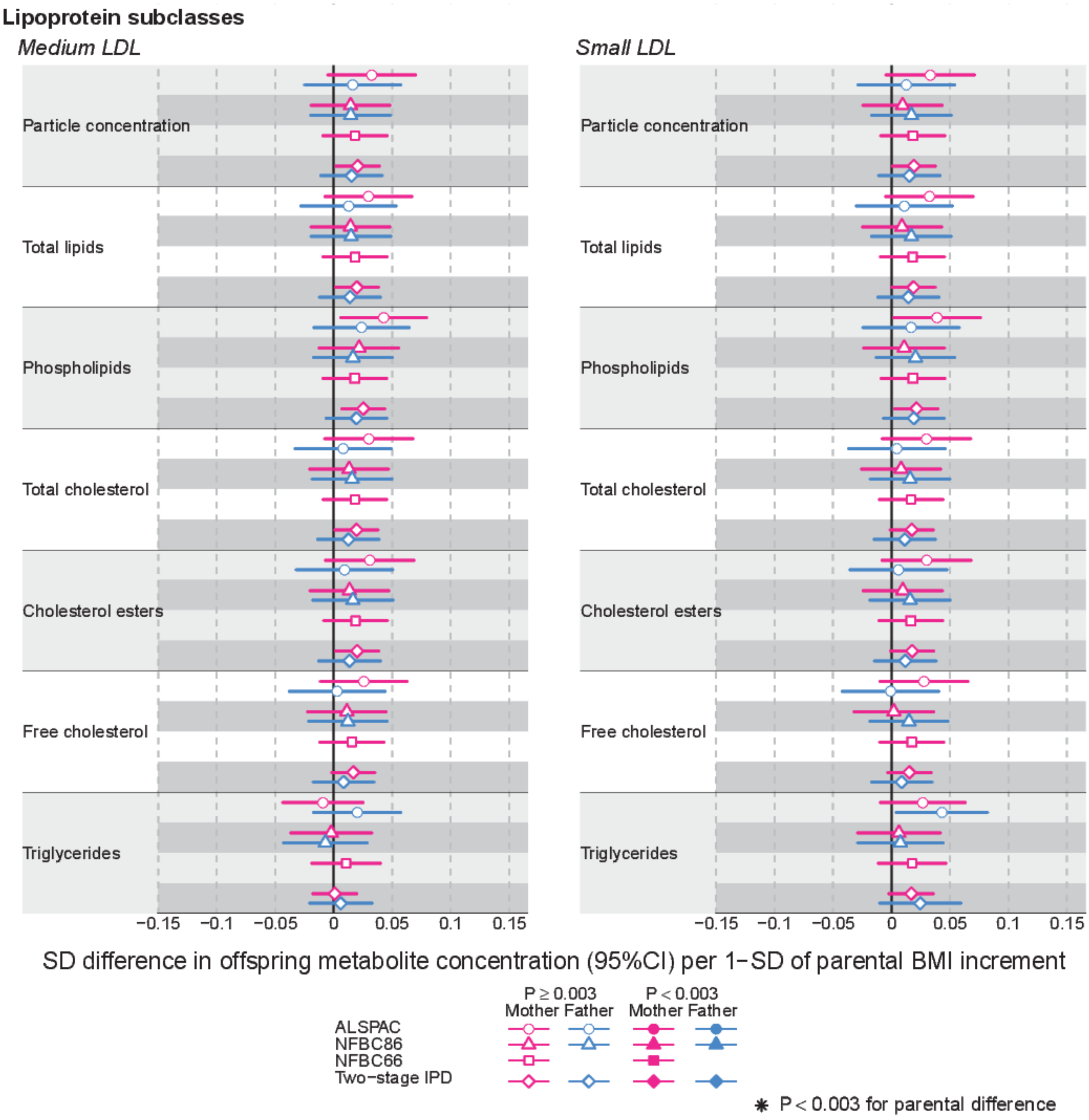

S3 Fig *continued*.

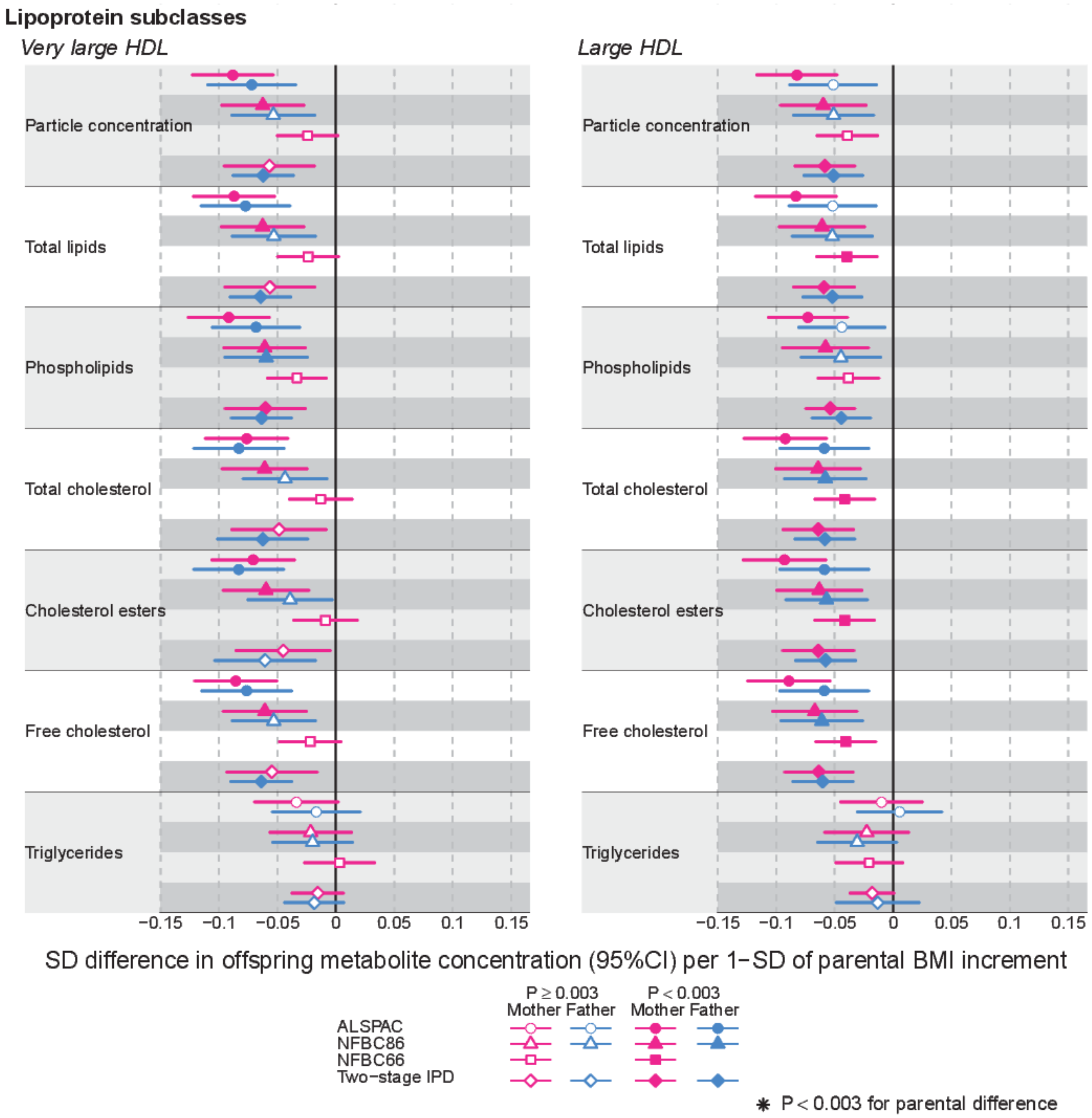

S3 Fig *continued*.

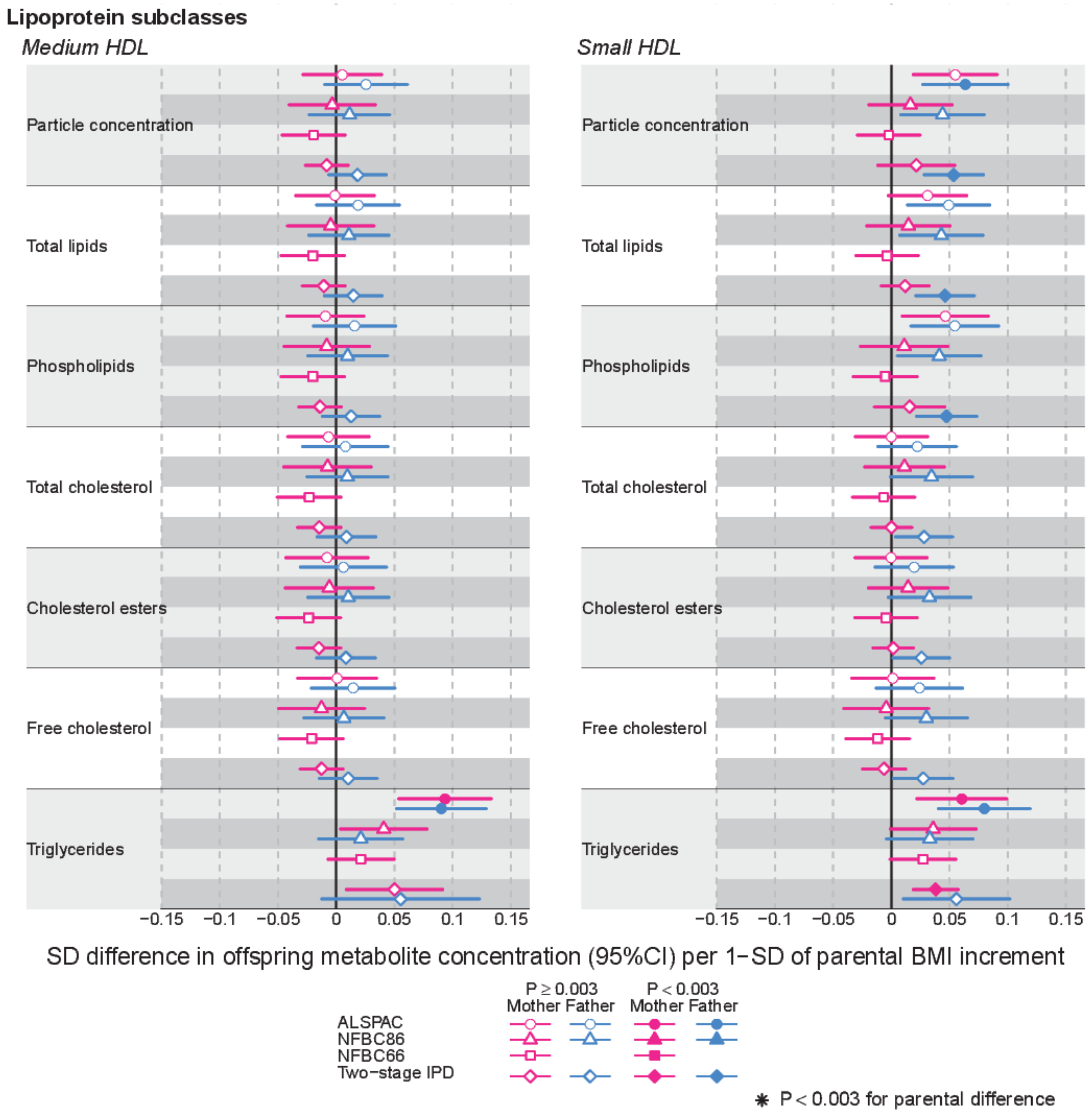

S3 Fig *continued*.

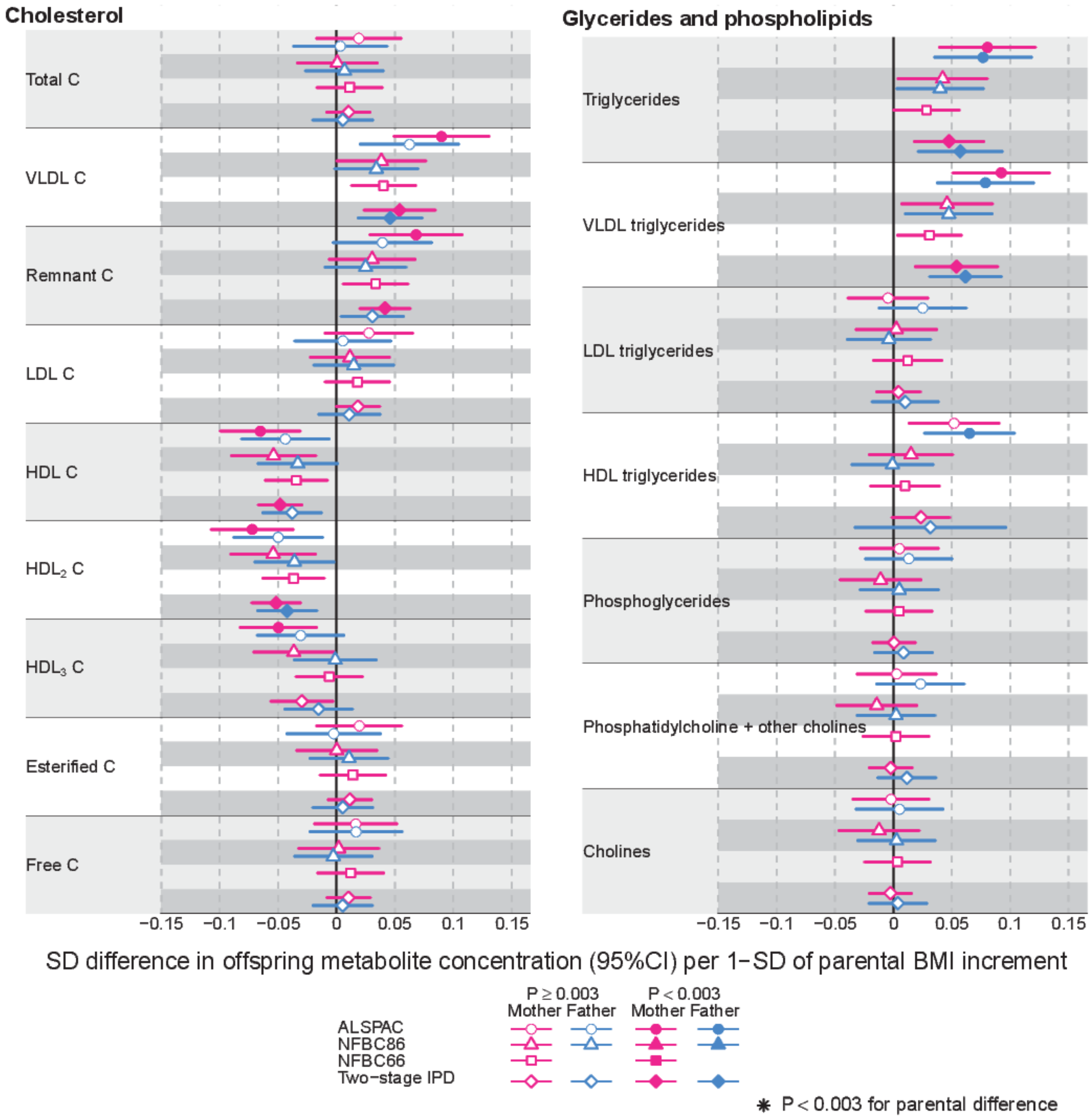

S3 Fig *continued*.

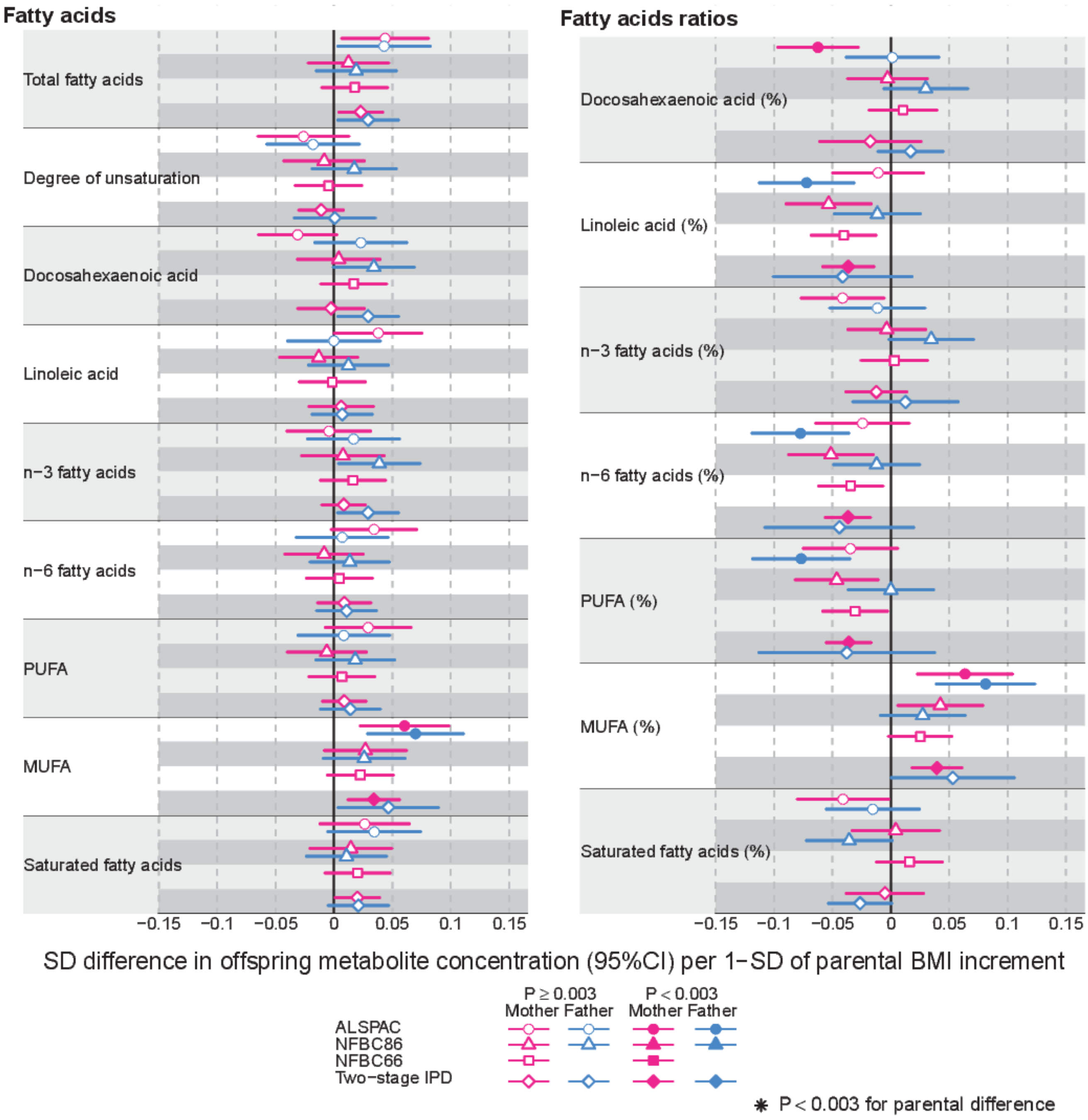

S3 Fig ***continued*****.**

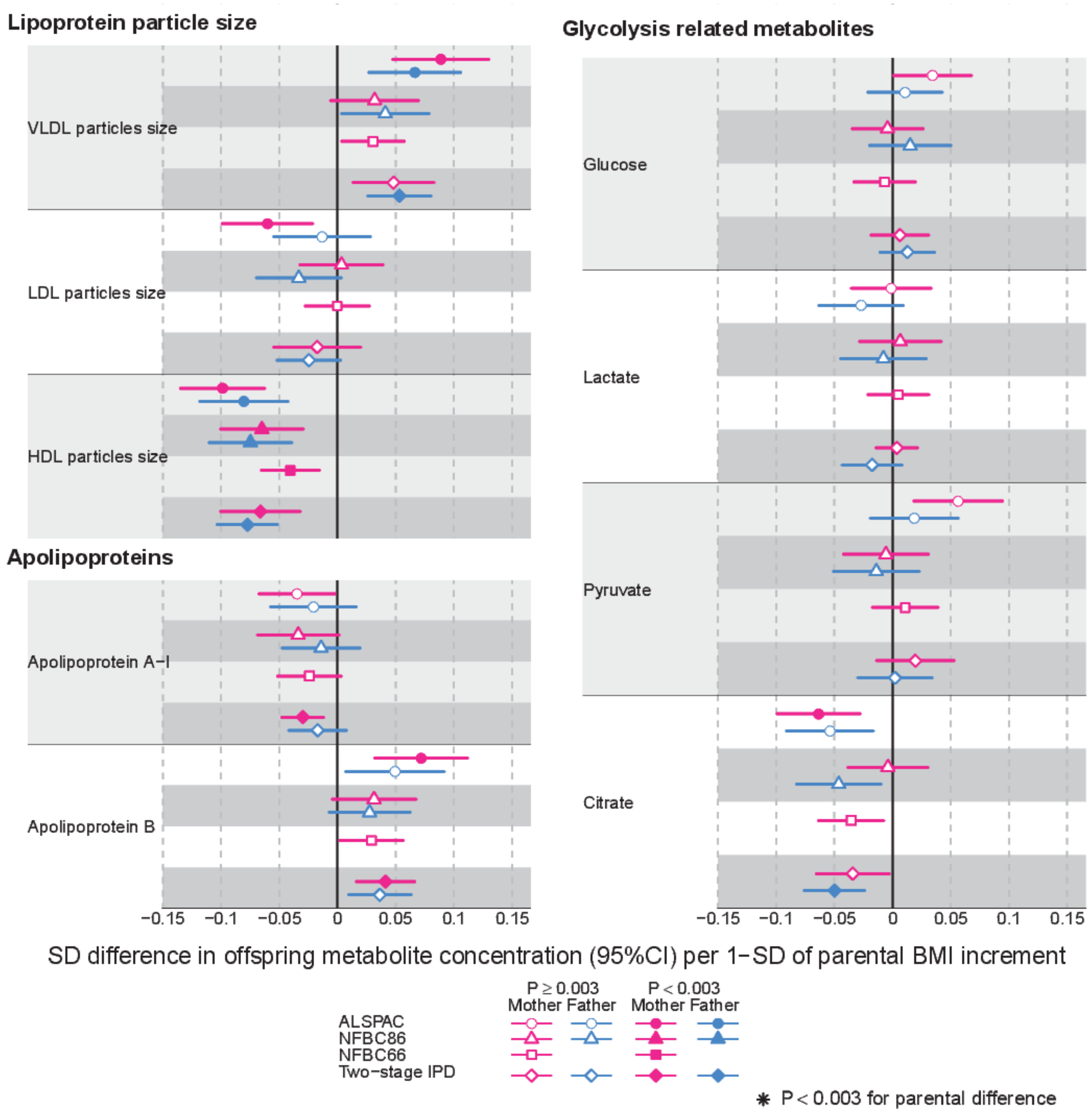

S3 Fig *continued*.

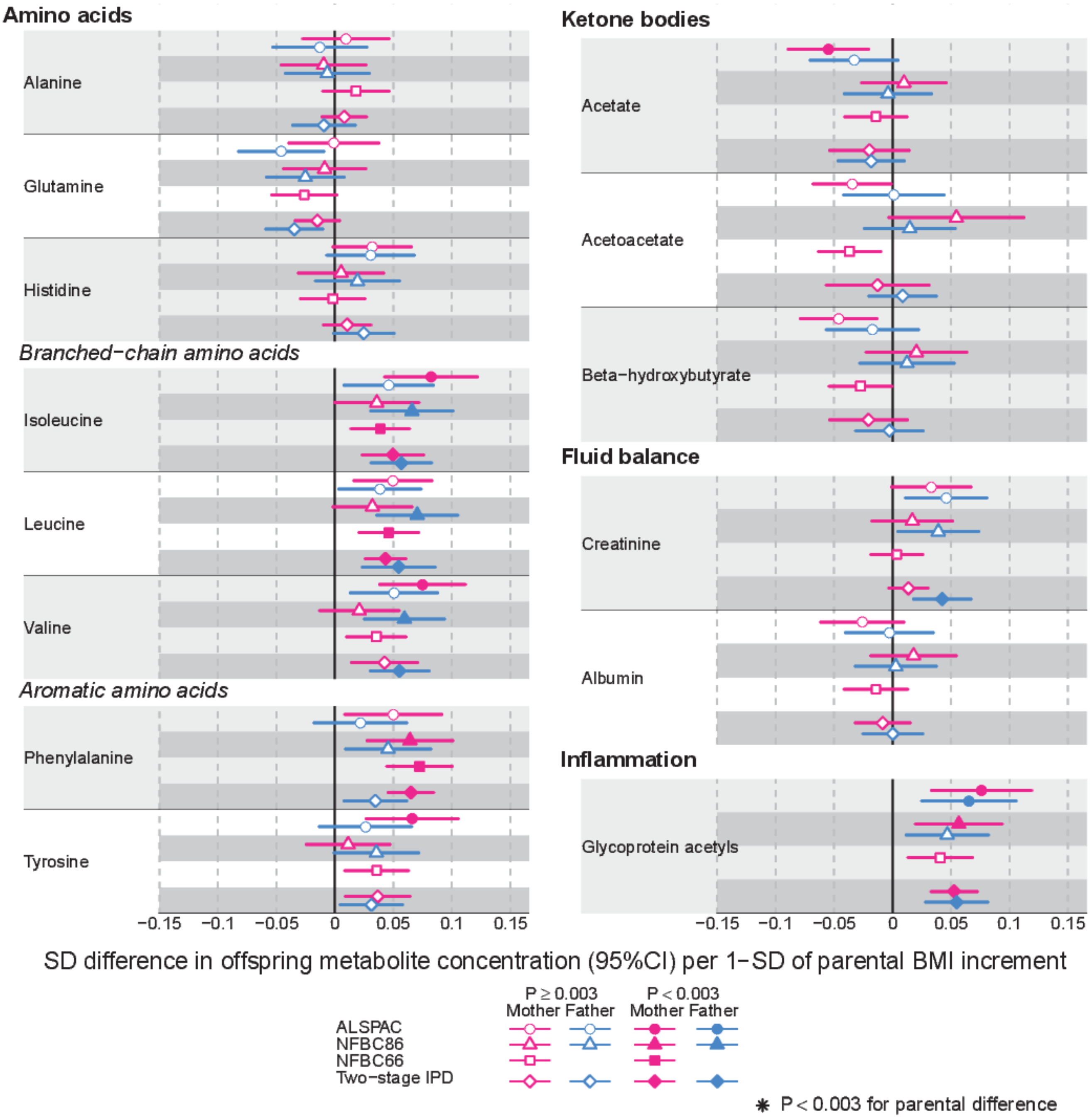

Associations were adjusted for parental age, smoking status, education, head of household social class, maternal parity, offspring's age at blood collection and sex. Results are shown in SD-scaled concentration units of outcome, changes in absolute concentration units are listed in S4-S6 Tables. Error bars= 95% confidence intervals (CI). VLDL=very-low-density lipoprotein; IDL=intermediate-density lipoprotein; LDL=low-density lipoprotein; HDL= high-density lipoprotein; C= cholesterol; MUFA=monounsaturated fatty acids; PUFA=polyunsaturated fatty acids.
