# Supplementary material for: Association of pre-pregnancy body mass index with offspring metabolic profile: Analyses of 3 European prospective birth cohorts
Source: PLoS Med. 2017 Aug 22;14(8):e1002376. doi: 10.1371/journal.pmed.1002376 (PMC5568725; doi:10.1371/journal.pmed.1002376)
Supplement: S4 Fig — (PDF) [file pmed.1002376.s004.pdf]

S4 Fig. *One-stage IPD meta-analysis: offspring lipoprotein, lipids and metabolite differences in means in SD units per 1-SD higher maternal (pink) or paternal (blue) BMI, meta-analysed across ALSPAC, NFBC86 and NFBC66 cohorts.*

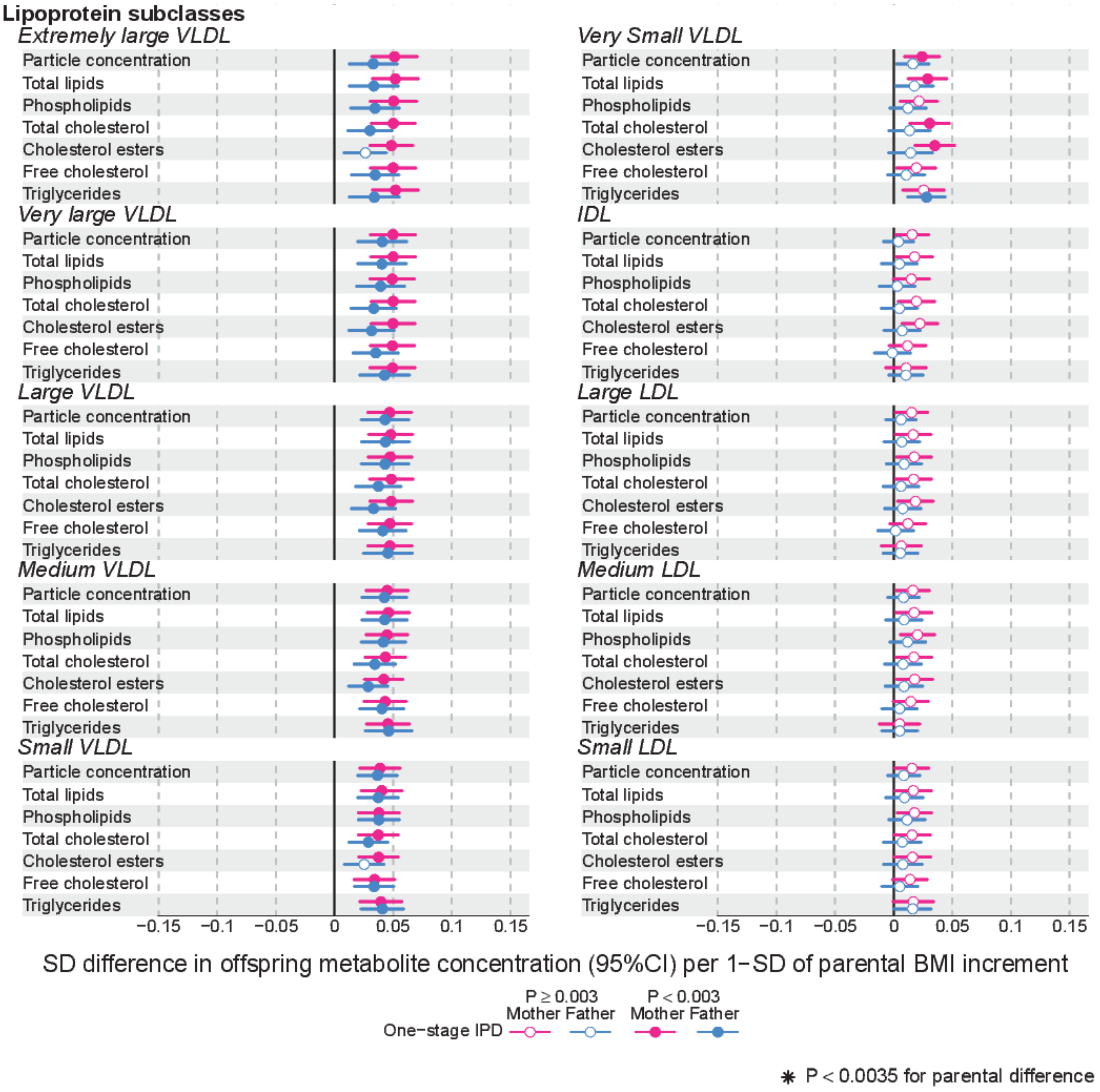

S4 Fig *continued*.

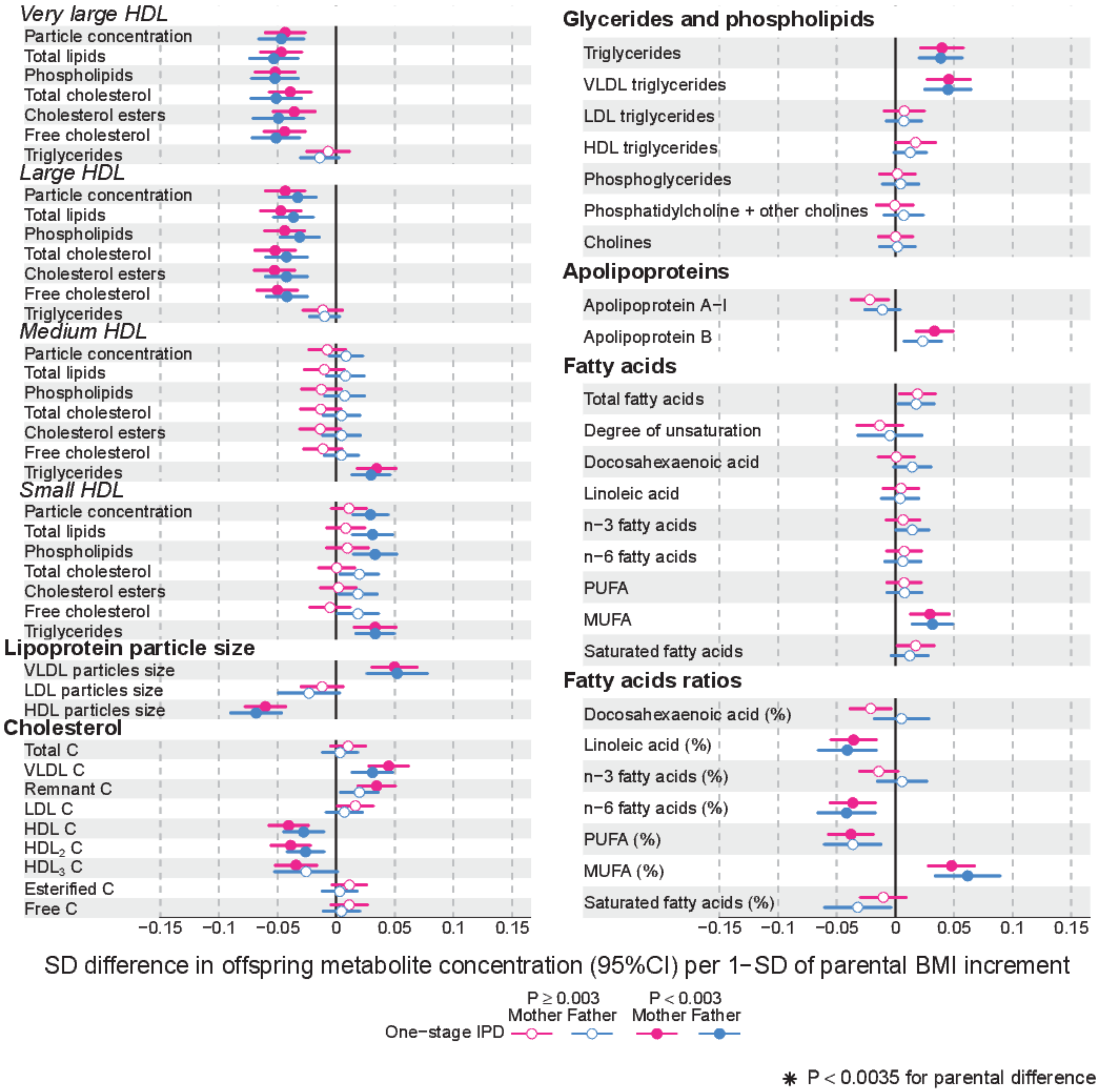

S4 Fig *continued*.

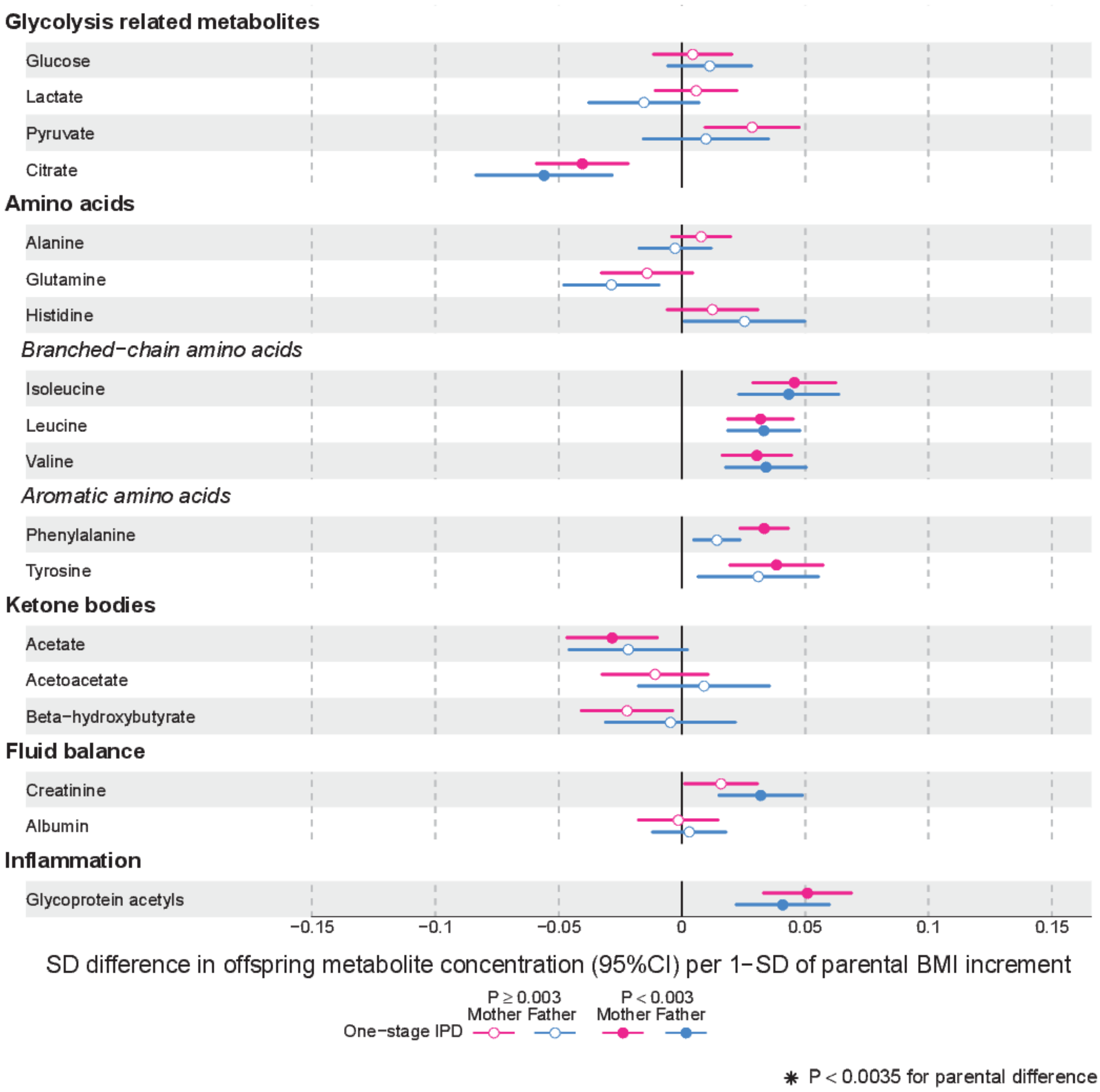

Associations were adjusted for parental age, smoking, education, head of household social class, maternal parity, offspring's age at blood collection, sex and cohort membership. Error bars= 95% confidence intervals (CI). VLDL=very-low-density lipoprotein; IDL=intermediate-density lipoprotein; LDL=low-density lipoprotein; HDL= high-density lipoprotein; C= cholesterol; MUFA=monounsaturated fatty acids; PUFA=polyunsaturated fatty acids.
