# Supplementary material for: Association of pre-pregnancy body mass index with offspring metabolic profile: Analyses of 3 European prospective birth cohorts
Source: PLoS Med. 2017 Aug 22;14(8):e1002376. doi: 10.1371/journal.pmed.1002376 (PMC5568725; doi:10.1371/journal.pmed.1002376)
Supplement: S5 Fig — (PDF) [file pmed.1002376.s005.pdf]

S5 Fig. *Linear fit between two and one-stage individual participant data (IPD) meta-analysis for mother (left panel; pink dashed line) and father (right panel; blue dashed line) models.*

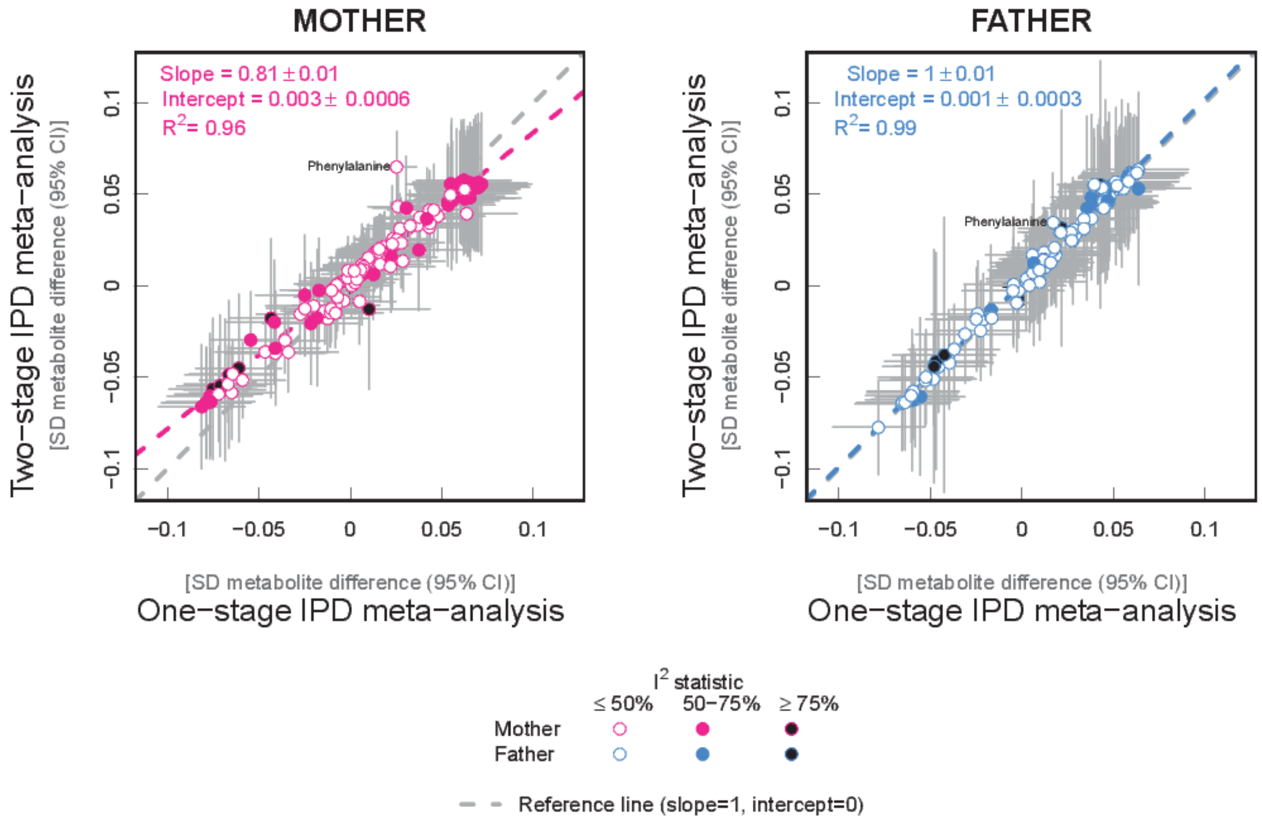

Each dot represents a metabolite and the positions of the dots are determined by difference in mean offspring metabolite (in SD units) for each increase of 1-SD parental BMI determined by one-stage IPD (x-axis) and two-stage IPD (y-axis) meta-analyses. The horizontal grey lines on each dot denote the confidence intervals (CI) for one-stage IPD associations and the vertical grey lines indicate the CI for two-stage IPD estimates. A linear fit of the overall correspondence summarizes the similarity in magnitude between one and two-stage associations (pink and blue dashed lines for maternal and paternal associations, respectively). A slope of 1 with an intercept of 0 (dashed grey line), with all dots sitting on that line (R^2=1), would indicate that one and two-stage IPD estimates had the same magnitude and direction. Results are shown in SD-scaled concentration units of outcome, differences in absolute concentration units are listed in S3-S4 Tables. I^2 statistics indicates between-cohort heterogeneity (very low ≤ 50%; substantial=50-75%; very high ≥ 75%).
