# Supplementary material for: Association of pre-pregnancy body mass index with offspring metabolic profile: Analyses of 3 European prospective birth cohorts
Source: PLoS Med. 2017 Aug 22;14(8):e1002376. doi: 10.1371/journal.pmed.1002376 (PMC5568725; doi:10.1371/journal.pmed.1002376)
Supplement: S6 Fig — (PDF) [file pmed.1002376.s006.pdf]

**S6 Fig.** *One-stage IPD meta-analysis: offspring lipoprotein, lipids and metabolite differences in means in SD units* per *1-SD higher maternal (pink), paternal (blue) or offspring (green) BMI, meta-analysed across ALSPAC and NFBC86 cohorts.*

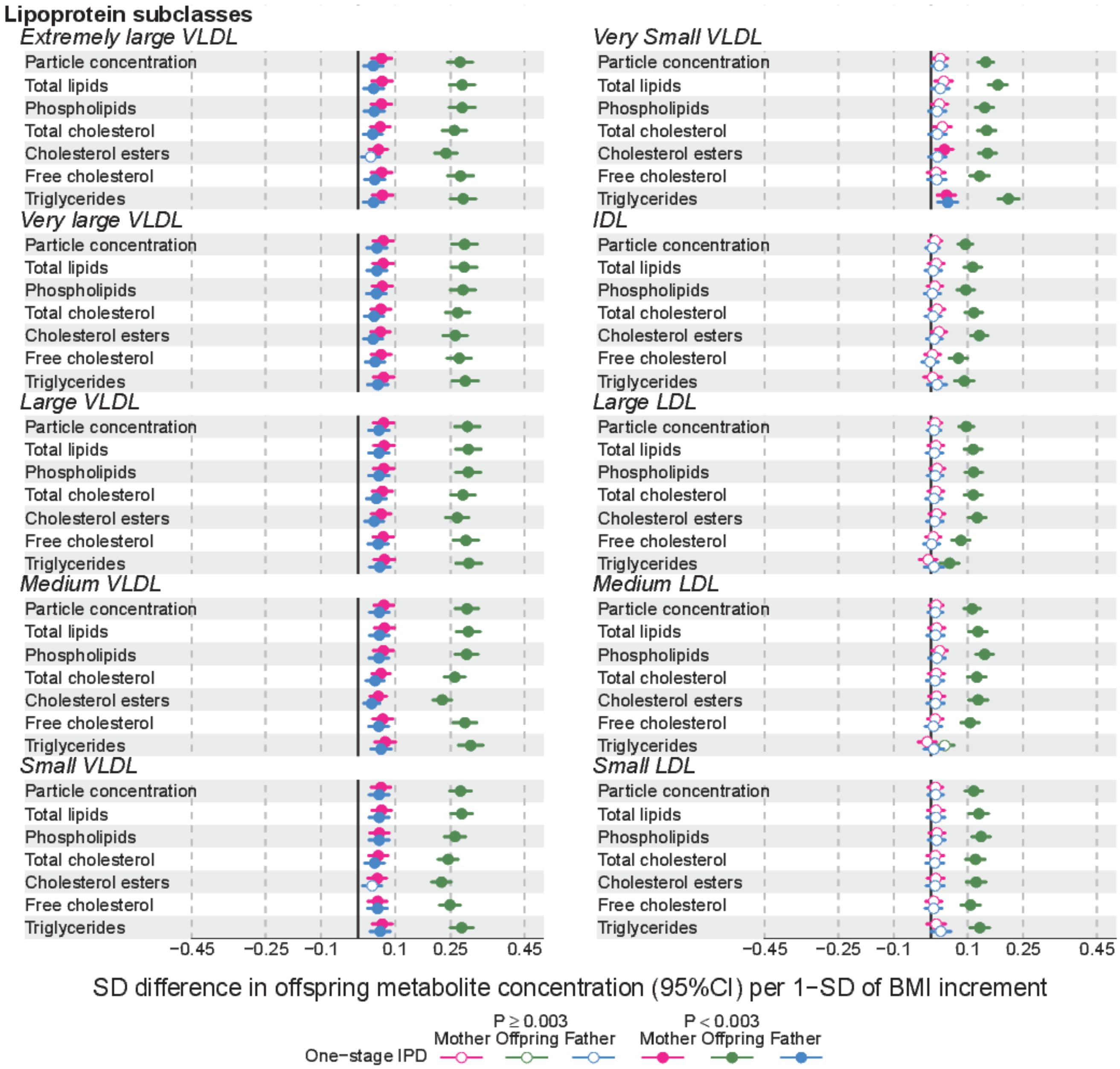

S6 Fig *continued*.

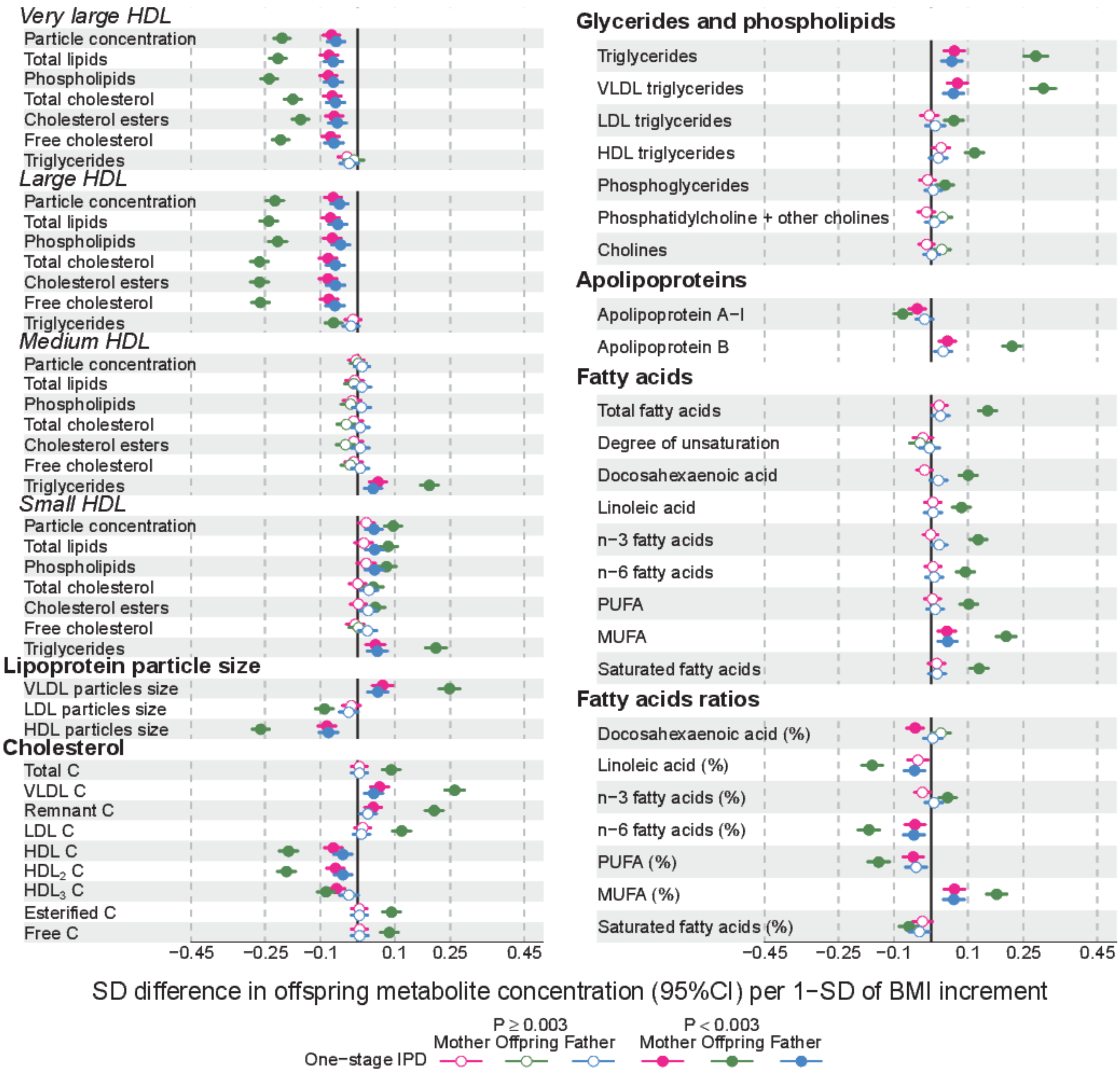

**S6 Fig *continued*.**

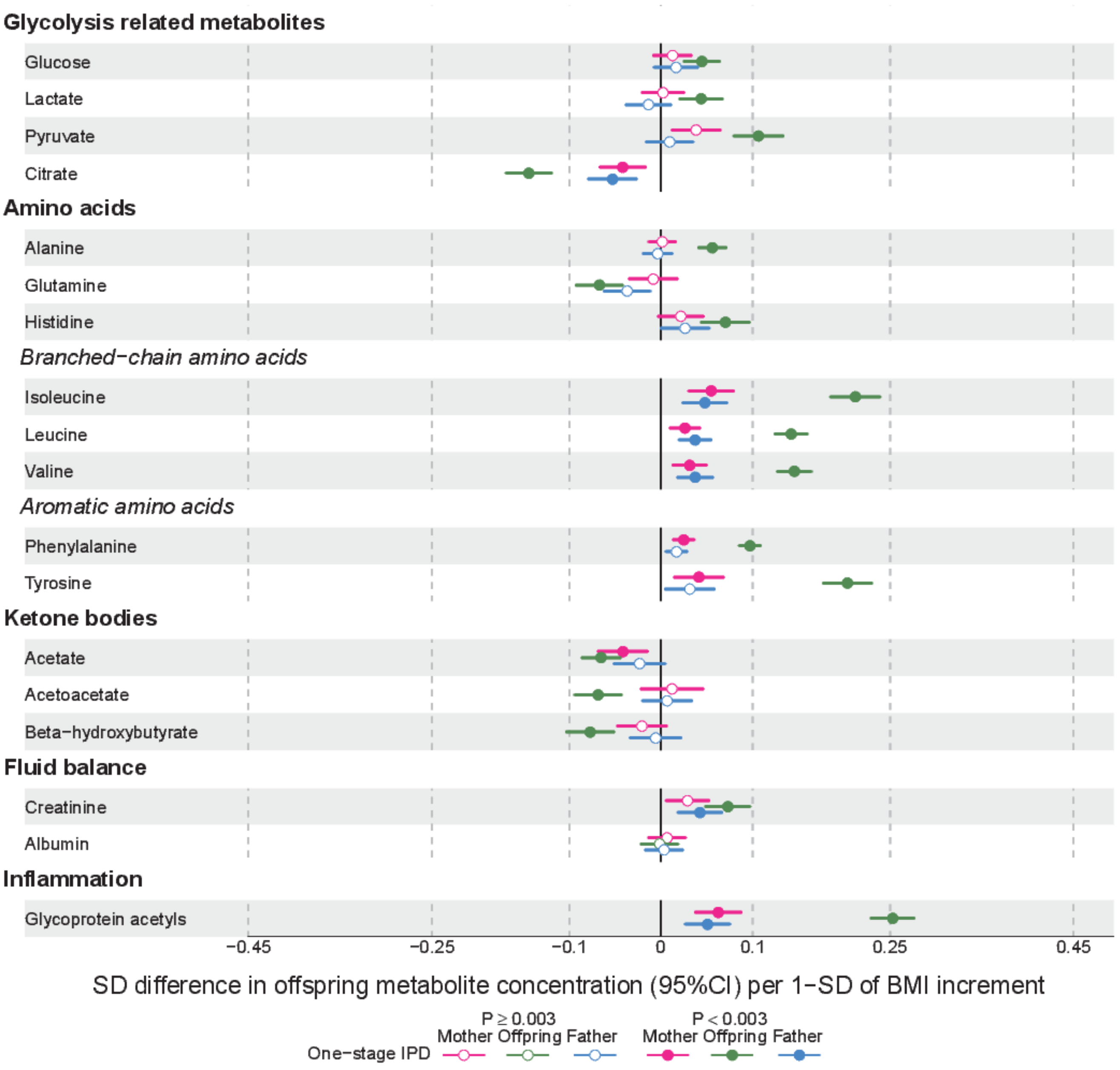


In parental models, associations were adjusted for parental age, smoking status, education, head of household social class, maternal parity, offspring's age at blood collection, sex and cohorts membership. In offspring models, associations were adjusted for offspring's age at blood collection, sex, head of household social class and cohorts membership. Results are shown in SD-scaled concentration units of outcome and error bars denote 95% CI. *VLDL=very-low-density lipoprotein; IDL=intermediate-density lipoprotein; LDL=low-density lipoprotein; HDL= high-density lipoprotein; C= cholesterol; MUFA=monounsaturated fatty acids; PUFA=polyunsaturated fatty acids.*
