# Supplementary material for: Association of pre-pregnancy body mass index with offspring metabolic profile: Analyses of 3 European prospective birth cohorts
Source: PLoS Med. 2017 Aug 22;14(8):e1002376. doi: 10.1371/journal.pmed.1002376 (PMC5568725; doi:10.1371/journal.pmed.1002376)
Supplement: S1 Table — (PDF) [file pmed.1002376.s008.pdf]

S1 Table. Characteristics of the three study populations.

	ALSPAC [N= 2440]			NFBC86 [N= 2937]			NFBC66 [N= 4874]	
	Mother	Father	Offspring	Mother	Father	Offspring	Mother	Offspring
**Age [years]**	29.39 (4.33)	31.38 (5.37)	17.14 (1.08)	28.08 (5.02)	30.31 (5.44)	16.01 (0.37)	28.00 (6.60)	31.10 (0.36)
**Males [%]**	-	-	48	-	-	50	-	48
**BMI [kg/m^2]**	22.93 (3.83)	25.11 (3.14)	22.37 (3.78)	22.20 (3.09)	23.98 (2.63)	21.05 (3.29)	23.22 (3.25)	24.61 (4.13)
underweight [%]	5	1	10	6	0	19	3	2
normal [%]	75	52	70	79	68	70	74	58
overweigh [%]	14	40	14	12	29	8	19	31
obese [%]	6	7	7	3	3	3	4	9
**Parity**	0.69 (0.84)			1.37 (1.68)			2.86 (2.20)	
**Smoking [%]**								
Non-smoker	79	74		77	67		81	
Pre/early pregnancy	11			14			7	
Through pregnancy	10			9			13	
Smoker		26			33			

Trios of mother-father-offspring (ALSPAC, NFBC86) or pairs of mother-offspring (NFBC66) who contributed to at least one pair of exposure/outcome analysis. Values are mean (SD) or percentage. Body Mass Index (BMI) categorized according to World Health Organization categories (underweight<18.5 kg/m2; normal 18.5-24.9 kg/m2; overweight 25.0-29.9 kg/m2; obese≥30.0 kg/m2) for descriptive purposes only, all statistical analyses were conducted using BMI as a continuous variable.

S1 Table *continued*.

category 1	ALSPAC [N=2440] category 2	Mother	Father	NFBC86 [N= 2937] category 2	Mother	Father	NFBC66 [N= 4874] category 2	Mother
**Education [%]**								
Basic or none	None	0	0	No occupational educ.	7	12	none or circulating school	1
							1-4 years of primary education	8
Secondary	CSE	8	13	Vocational course	9	15	5-8 years primary or early secondary	56
	Vocational	7	6	Vocational school	22	33	0.5-2 years of vocational school	16
	O level	35	22				> 2 years of vocational school	3
	A level	29	30				Secondary school	7
							Matriculation exam taken	4
Higher	Degree	21	29	Post-secondary college	36	22	Beyond secondary	1
				Polytechnic	3	2	Beyond matriculation exam	4
				University degree	10	10		
Other				Other education	8	5		
				Uncompleted educ.	4	1		
**Head of household social class (%)**								
**I**	I	20		entrepreneur	12		I	9
**II**	II	47		professional	9		II	20
**III**	III (non-manual)	22		skilled non-manual	29		III	58
	III (manual)	7		skilled manual	44			
**IV**	IV	3		unskilled or apprentice	3		IV	13
				no-occupation	3		V (no occupation)	0

Trios of mother-father-offspring (ALSPAC, NFBC86) or pairs of mother-offspring (NFBC66) who contributed to at least one pair of exposure/outcome analysis. Values are mean (SD) or percentage. Category 1= harmonized categories across the three cohorts used on one-stage individual participant data meta-analysis; Category 2= cohort specific categories used on two-stage individual participant (aggregate) data meta-analysis.
