# Supplementary material for: Association of pre-pregnancy body mass index with offspring metabolic profile: Analyses of 3 European prospective birth cohorts
Source: PLoS Med. 2017 Aug 22;14(8):e1002376. doi: 10.1371/journal.pmed.1002376 (PMC5568725; doi:10.1371/journal.pmed.1002376)
Supplement: S2 Table — (PDF) [file pmed.1002376.s009.pdf]

S2 Table. Characteristics of the metabolic traits classes.

**Metabolic traits class (*N*=number of traits)**	**Metabolic traits**
Extremely large VLDL (7)	particle concentration, total lipids, phospholipids, total cholesterol, cholesterol esters, free cholesterol, triglycerides
Very large VLDL (7)	particle concentration, total lipids, phospholipids, total cholesterol, cholesterol esters, free cholesterol, triglycerides
Large VLDL (7)	particle concentration, total lipids, phospholipids, total cholesterol, cholesterol esters, free cholesterol, triglycerides
Medium VLDL (7)	particle concentration, total lipids, phospholipids, total cholesterol, cholesterol esters, free cholesterol, triglycerides
Small VLDL (7)	particle concentration, total lipids, phospholipids, total cholesterol, cholesterol esters, free cholesterol, triglycerides
Very small VLDL (7)	particle concentration, total lipids, phospholipids, total cholesterol, cholesterol esters, free cholesterol, triglycerides
IDL (7)	particle concentration, total lipids, phospholipids, total cholesterol, cholesterol esters, free cholesterol, triglycerides
Large LDL (7)	particle concentration, total lipids, phospholipids, total cholesterol, cholesterol esters, free cholesterol, triglycerides
Medium LDL (7)	particle concentration, total lipids, phospholipids, total cholesterol, cholesterol esters, free cholesterol, triglycerides
Small LDL (7)	particle concentration, total lipids, phospholipids, total cholesterol, cholesterol esters, free cholesterol, triglycerides
Very large HDL (7)	particle concentration, total lipids, phospholipids, total cholesterol, cholesterol esters, free cholesterol, triglycerides
Large HDL (7)	particle concentration, total lipids, phospholipids, total cholesterol, cholesterol esters, free cholesterol, triglycerides
Medium HDL (7)	particle concentration, total lipids, phospholipids, total cholesterol, cholesterol esters, free cholesterol, triglycerides
Small HDL (7)	particle concentration, total lipids, phospholipids, total cholesterol, cholesterol esters, free cholesterol, triglycerides
Cholesterol (9)	total, VLDL, remnant, LDL, HDL, HDL_2, HDL_3, esterified, and free cholesterol
Glycerides and phospholipids (7)	triglycerides, VLDL, LDL, and HDL triglycerides, phosphoglycerides, phosphatidylcholine, and cholines.
Fatty acids (9)	total fatty acids, degree of unsaturation, docosahexaenoic acid, linoleic acid, n-3 fatty acids, n-6 fatty acids, saturated fatty acids, PUFA, and MUFA
Fatty acids ratios (7)	docosahexaenoic acid (%), linoleic acid (%), n-3 fatty acids (%), n-6 fatty acids (%), PUFA (%), MUFA (%), and saturated fatty acids (%)
Lipoprotein particle size (3)	VLDL, LDL and HDL particles sizes
Apolipoproteins (2)	apolipoprotein A-I and B
Glycolysis related metabolites (4)	glucose, lactate, pyruvate, and citrate
Amino acids (3)	alanine, glutamine and histidine

Branched-chain amino acids (3)	isoleucine, leucine and valine
Aromatic amino acids (2)	phenylalanine and tyrosine
Ketone bodies (3)	acetate, acetoacetate, and beta-hydroxybutyrate
Fluid balance (2)	creatinine and albumin
Inflammation (1)	glycoprotein acetyls

VLDL=very-low-density lipoprotein; IDL=intermediate-density lipoprotein; LDL=low-density lipoprotein; HDL= high-density lipoprotein; MUFA=monounsaturated fatty acids; PUFA=polyunsaturated fatty acids.
