# Supplementary material for: Association of pre-pregnancy body mass index with offspring metabolic profile: Analyses of 3 European prospective birth cohorts
Source: PLoS Med. 2017 Aug 22;14(8):e1002376. doi: 10.1371/journal.pmed.1002376 (PMC5568725; doi:10.1371/journal.pmed.1002376)
Supplement: S3 Table — (PDF) [file pmed.1002376.s010.pdf]

S3 Table. One-stage individual participant data (IPD) meta-analysis: offspring lipoproteins, lipids and metabolites absolute concentration differences per 1-SD higher parental BMI.

One stage-Individual Participant Data meta-analysis			
Metabolites	mother	father	P_{boot}
**Lipoprotein subclasses**			
Extremely large VLDL			
Particle concentration (mol/l)	6×10^{-12} (3×10^{-12}, 8×10^{-12}) p=4×10^{-6}	4×10^{-12} (1×10^{-12}, 6×10^{-12}) p=1×10^{-3}	0.25
Total lipids (mmol/l)	1×10^{-3} (7×10^{-4}, 2×10^{-3}) p=4×10^{-6}	8×10^{-4} (3×10^{-4}, 1×10^{-3}) p=2×10^{-3}	0.26
Phospholipids (mmol/l)	1×10^{-4} (8×10^{-5}, 2×10^{-4}) p=9×10^{-6}	1×10^{-4} (4×10^{-5}, 2×10^{-4}) p=1×10^{-3}	0.35
Total cholesterol (mmol/l)	2×10^{-4} (1×10^{-4}, 3×10^{-4}) p=8×10^{-6}	2×10^{-4} (6×10^{-5}, 2×10^{-4}) p=2×10^{-3}	0.28
Cholesterol esters (mmol/l)	1×10^{-4} (7×10^{-5}, 2×10^{-4}) p=1×10^{-5}	8×10^{-5} (3×10^{-5}, 1×10^{-4}) p=4×10^{-3}	0.24
Free cholesterol (mmol/l)	9.5×10^{-5} (5×10^{-5}, 1×10^{-4}) p=8×10^{-6}	7×10^{-5} (3×10^{-5}, 1×10^{-4}) p=8×10^{-4}	0.38
Triglycerides (mmol/l)	9×10^{-4} (5×10^{-4}, 1×10^{-3}) p=4×10^{-6}	6×10^{-4} (2×10^{-4}, 9×10^{-4}) p=2×10^{-3}	0.24
Very large VLDL			
Particle concentration (mol/l)	3×10^{-11} (2×10^{-11}, 5×10^{-11}) p=2×10^{-6}	3×10^{-11} (1×10^{-11}, 4×10^{-11}) p=1×10^{-4}	0.45
Total lipids (mmol/l)	3×10^{-3} (2×10^{-3}, 4×10^{-3}) p=2×10^{-6}	3×10^{-3} (1×10^{-3}, 4×10^{-3}) p=1×10^{-4}	0.46
Phospholipids (mmol/l)	5×10^{-4} (3×10^{-4}, 7×10^{-4}) p=3×10^{-6}	4×10^{-4} (2×10^{-4}, 6×10^{-4}) p=2×10^{-4}	0.46
Total cholesterol (mmol/l)	6×10^{-4} (4×10^{-4}, 9×10^{-4}) p=6×10^{-6}	5×10^{-4} (2×10^{-4}, 7×10^{-4}) p=7×10^{-4}	0.35
Cholesterol esters (mmol/l)	3×10^{-4} (2×10^{-4}, 5×10^{-4}) p=7×10^{-6}	2×10^{-4} (9×10^{-5}, 4×10^{-4}) p=1×10^{-3}	0.30
Free cholesterol (mmol/l)	3×10^{-4} (2×10^{-4}, 4×10^{-4}) p=6×10^{-6}	2×10^{-4} (1×10^{-4}, 4×10^{-4}) p=4×10^{-4}	0.43
Triglycerides (mmol/l)	2×10^{-3} (1×10^{-3}, 3×10^{-3}) p=2×10^{-6}	2×10^{-3} (8×10^{-4}, 2×10^{-3}) p=8×10^{-5}	0.49
Large VLDL			
Particle concentration (mol/l)	2×10^{-10} (10×10^{-11}, 2×10^{-10}) p=2×10^{-6}	1×10^{-10} (8×10^{-11}, 2×10^{-10}) p=3×10^{-5}	0.62
Total lipids (mmol/l)	0.01 (0.01, 0.01) p=1×10^{-6}	0.01 (5×10^{-3}, 0.01) p=3×10^{-5}	0.58
Phospholipids (mmol/l)	2×10^{-3} (1×10^{-3}, 3×10^{-3}) p=1×10^{-6}	2×10^{-3} (8×10^{-4}, 2×10^{-3}) p=3×10^{-5}	0.59
Total cholesterol (mmol/l)	2×10^{-3} (1×10^{-3}, 3×10^{-3}) p=2×10^{-6}	2×10^{-3} (9×10^{-4}, 3×10^{-3}) p=1×10^{-4}	0.44
Cholesterol esters (mmol/l)	1×10^{-3} (7×10^{-4}, 2×10^{-3}) p=3×10^{-6}	9×10^{-4} (4×10^{-4}, 1×10^{-3}) p=5×10^{-4}	0.34
Free cholesterol (mmol/l)	1×10^{-3} (7×10^{-4}, 2×10^{-3}) p=2×10^{-6}	9.9×10^{-4} (5×10^{-4}, 1×10^{-3}) p=5×10^{-5}	0.58
Triglycerides (mmol/l)	0.01 (4×10^{-3}, 0.01) p=1×10^{-6}	0.01 (3×10^{-3}, 0.01) p=2×10^{-5}	0.65
Medium VLDL			
Particle concentration (mol/l)	4×10^{-10} (3×10^{-10}, 6×10^{-10}) p=5×10^{-7}	4×10^{-10} (2×10^{-10}, 5×10^{-10}) p=1×10^{-5}	0.61
Total lipids (mmol/l)	0.02 (0.01, 0.02) p=4×10^{-7}	0.01 (0.01, 0.02) p=1×10^{-5}	0.57
Phospholipids (mmol/l)	3×10^{-3} (2×10^{-3}, 4×10^{-3}) p=6×10^{-7}	2×10^{-3} (1×10^{-3}, 4×10^{-3}) p=2×10^{-5}	0.60
Total cholesterol (mmol/l)	4×10^{-3} (2×10^{-3}, 0.01) p=9×10^{-7}	3×10^{-3} (2×10^{-3}, 5×10^{-3}) p=1×10^{-4}	0.40
Cholesterol esters (mmol/l)	2×10^{-3} (1×10^{-3}, 3×10^{-3}) p=1×10^{-6}	2×10^{-3} (7×10^{-4}, 2×10^{-3}) p=7×10^{-4}	0.26
Free cholesterol (mmol/l)	2×10^{-3} (1×10^{-3}, 2×10^{-3}) p=2×10^{-6}	2×10^{-3} (8×10^{-4}, 2×10^{-3}) p=3×10^{-5}	0.66
Triglycerides (mmol/l)	0.01 (0.01, 0.01) p=3×10^{-7}	0.01 (4×10^{-3}, 0.01) p=5×10^{-6}	0.67
Small VLDL			
Particle concentration (mol/l)	5×10^{-10} (3×10^{-10}, 7×10^{-10}) p=3×10^{-6}	4×10^{-10} (2×10^{-10}, 6×10^{-10}) p=2×10^{-5}	0.83
Total lipids (mmol/l)	0.01 (0.01, 0.01) p=2×10^{-6}	0.01 (5×10^{-3}, 0.01) p=3×10^{-5}	0.76
Phospholipids (mmol/l)	2×10^{-3} (1×10^{-3}, 3×10^{-3}) p=1×10^{-5}	2×10^{-3} (9.9×10^{-4}, 3×10^{-3}) p=2×10^{-5}	0.99
Total cholesterol (mmol/l)	3×10^{-3} (2×10^{-3}, 0.01) p=2×10^{-5}	3×10^{-3} (1×10^{-3}, 4×10^{-3}) p=7×10^{-4}	0.56
Cholesterol esters (mmol/l)	2×10^{-3} (1×10^{-3}, 3×10^{-3}) p=4×10^{-5}	2×10^{-3} (6×10^{-4}, 3×10^{-3}) p=3×10^{-3}	0.39
Free cholesterol (mmol/l)	1×10^{-3} (6×10^{-4}, 2×10^{-3}) p=5×10^{-5}	1×10^{-3} (6×10^{-4}, 2×10^{-3}) p=8×10^{-5}	0.99
Triglycerides (mmol/l)	4×10^{-3} (2×10^{-3}, 0.01) p=2×10^{-6}	4×10^{-3} (2×10^{-3}, 0.01) p=1×10^{-5}	0.87
Very Small VLDL			
Particle concentration (mol/l)	3×10^{-10} (7×10^{-11}, 5×10^{-10}) p=0.01	2×10^{-10} (4×10^{-11}, 4×10^{-10}) p=0.02	0.81
Total lipids (mmol/l)	4×10^{-3} (1×10^{-3}, 0.01) p=2×10^{-3}	3×10^{-3} (3×10^{-4}, 0.01) p=0.03	0.54
Phospholipids (mmol/l)	9×10^{-4} (1×10^{-4}, 2×10^{-3}) p=0.02	7×10^{-4} (-2×10^{-4}, 2×10^{-3}) p=0.12	0.64
Total cholesterol (mmol/l)	2×10^{-3} (6×10^{-4}, 4×10^{-3}) p=0.01	1×10^{-3} (-4×10^{-4}, 3×10^{-3}) p=0.13	0.36
Cholesterol esters (mmol/l)	2×10^{-3} (7×10^{-4}, 3×10^{-3}) p=2×10^{-3}	9×10^{-4} (-3×10^{-4}, 2×10^{-3}) p=0.13	0.21
Free cholesterol (mmol/l)	3×10^{-4} (-1×10^{-4}, 7×10^{-4}) p=0.18	3×10^{-4} (-2×10^{-4}, 8×10^{-4}) p=0.19	0.98
Triglycerides (mmol/l)	1×10^{-3} (4×10^{-4}, 2×10^{-3}) p=7×10^{-4}	1×10^{-3} (5×10^{-4}, 2×10^{-3}) p=5×10^{-4}	0.83
IDL			
Particle concentration (mol/l)	4×10^{-10} (-1×10^{-10}, 9×10^{-10}) p=0.14	2×10^{-10} (-4×10^{-10}, 7×10^{-10}) p=0.54	0.53
Total lipids (mmol/l)	5×10^{-3} (-1×10^{-3}, 0.01) p=0.12	2×10^{-3} (-4×10^{-3}, 0.01) p=0.53	0.49
Phospholipids (mmol/l)	9×10^{-4} (-5×10^{-4}, 2×10^{-3}) p=0.22	3×10^{-4} (-1×10^{-3}, 2×10^{-3}) p=0.70	0.54

S3 Table. One-stage individual participant data (IPD) meta-analysis: offspring lipoproteins, lipids and metabolites absolute concentration differences per 1-SD higher parental BMI.

**One stage-Individual Participant Data meta-analysis**			
Metabolites	mother	father	P_{boot}
Total cholesterol (mmol/l)	4×10^{-3} $(-4\times10^{-4}, 0.01)$ p=0.08	1×10^{-3} $(-3\times10^{-3}, 0.01)$ p=0.54	0.40
Cholesterol esters (mmol/l)	3×10^{-3} $(3\times10^{-4}, 0.01)$ p=0.03	1×10^{-3} $(-2\times10^{-3}, 4\times10^{-3})$ p=0.37	0.35
Free cholesterol (mmol/l)	4×10^{-4} $(-8\times10^{-4}, 2\times10^{-3})$ p=0.53	-9×10^{-5} $(-1\times10^{-3}, 1\times10^{-3})$ p=0.88	0.56
Triglycerides (mmol/l)	2×10^{-4} $(-4\times10^{-4}, 8\times10^{-4})$ p=0.55	5×10^{-4} $(-2\times10^{-4}, 1\times10^{-3})$ p=0.16	0.51
Large LDL			
Particle concentration (mol/l)	7×10^{-10} $(-2\times10^{-10}, 2\times10^{-9})$ p=0.13	5×10^{-10} $(-5\times10^{-10}, 1\times10^{-9})$ p=0.34	0.71
Total lipids (mmol/l)	0.01 $(-2\times10^{-3}, 0.01)$ p=0.14	3×10^{-3} $(-4\times10^{-3}, 0.01)$ p=0.38	0.70
Phospholipids (mmol/l)	1×10^{-3} $(-9.7\times10^{-5}, 3\times10^{-3})$ p=0.07	9×10^{-4} $(-7\times10^{-4}, 2\times10^{-3})$ p=0.25	0.64
Total cholesterol (mmol/l)	4×10^{-3} $(-1\times10^{-3}, 0.01)$ p=0.13	2×10^{-3} $(-3\times10^{-3}, 0.01)$ p=0.43	0.62
Cholesterol esters (mmol/l)	3×10^{-3} $(-5\times10^{-4}, 0.01)$ p=0.09	2×10^{-3} $(-2\times10^{-3}, 0.01)$ p=0.33	0.62
Free cholesterol (mmol/l)	5×10^{-4} $(-7\times10^{-4}, 2\times10^{-3})$ p=0.40	1×10^{-4} $(-1\times10^{-3}, 1\times10^{-3})$ p=0.84	0.65
Triglycerides (mmol/l)	-1×10^{-4} $(-7\times10^{-4}, 5\times10^{-4})$ p=0.72	3×10^{-4} $(-4\times10^{-4}, 9\times10^{-4})$ p=0.45	0.41
Medium LDL			
Particle concentration (mol/l)	7×10^{-10} $(-9\times10^{-11}, 1\times10^{-9})$ p=0.08	5×10^{-10} $(-3\times10^{-10}, 1\times10^{-9})$ p=0.22	0.76
Total lipids (mmol/l)	4×10^{-3} $(-8\times10^{-4}, 0.01)$ p=0.11	3×10^{-3} $(-2\times10^{-3}, 0.01)$ p=0.27	0.76
Phospholipids (mmol/l)	1×10^{-3} $(2\times10^{-4}, 2\times10^{-3})$ p=0.02	7×10^{-4} $(-2\times10^{-4}, 2\times10^{-3})$ p=0.13	0.56
Total cholesterol (mmol/l)	3×10^{-3} $(-7\times10^{-4}, 0.01)$ p=0.12	2×10^{-3} $(-2\times10^{-3}, 0.01)$ p=0.32	0.72
Cholesterol esters (mmol/l)	2×10^{-3} $(-5\times10^{-4}, 5\times10^{-3})$ p=0.11	2×10^{-3} $(-1\times10^{-3}, 4\times10^{-3})$ p=0.28	0.74
Free cholesterol (mmol/l)	4×10^{-4} $(-2\times10^{-4}, 1\times10^{-3})$ p=0.19	2×10^{-4} $(-4\times10^{-4}, 9\times10^{-4})$ p=0.52	0.66
Triglycerides (mmol/l)	-9.5×10^{-5} $(-4\times10^{-4}, 2\times10^{-4})$ p=0.58	1×10^{-4} $(-2\times10^{-4}, 5\times10^{-4})$ p=0.51	0.37
Small LDL			
Particle concentration (mol/l)	6×10^{-10} $(-2\times10^{-10}, 1\times10^{-9})$ p=0.15	6×10^{-10} $(-3\times10^{-10}, 1\times10^{-9})$ p=0.22	0.92
Total lipids (mmol/l)	2×10^{-3} $(-7\times10^{-4}, 5\times10^{-3})$ p=0.15	2×10^{-3} $(-1\times10^{-3}, 4\times10^{-3})$ p=0.26	0.86
Phospholipids (mmol/l)	5×10^{-4} $(-8\times10^{-5}, 1\times10^{-3})$ p=0.09	5×10^{-4} $(-2\times10^{-4}, 1\times10^{-3})$ p=0.15	0.89
Total cholesterol (mmol/l)	1×10^{-3} $(-7\times10^{-4}, 3\times10^{-3})$ p=0.20	9×10^{-4} $(-1\times10^{-3}, 3\times10^{-3})$ p=0.38	0.80
Cholesterol esters (mmol/l)	1×10^{-3} $(-5\times10^{-4}, 3\times10^{-3})$ p=0.17	8×10^{-4} $(-9\times10^{-4}, 2\times10^{-3})$ p=0.35	0.78
Free cholesterol (mmol/l)	2×10^{-4} $(-2\times10^{-4}, 6\times10^{-4})$ p=0.38	1×10^{-4} $(-3\times10^{-4}, 5\times10^{-4})$ p=0.51	0.89
Triglycerides (mmol/l)	1×10^{-4} $(-7\times10^{-5}, 4\times10^{-4})$ p=0.18	2×10^{-4} $(7\times10^{-6}, 5\times10^{-4})$ p=0.04	0.56
Very large HDL			
Particle concentration (mol/l)	-1×10^{-8} $(-2\times10^{-8}, -8\times10^{-9})$ $p=3\times10^{-9}$	-1×10^{-8} $(-1\times10^{-8}, -6\times10^{-9})$ $p=2\times10^{-6}$	0.52
Total lipids (mmol/l)	-0.01 (-0.02, -0.01) $p=9.6\times10^{-10}$	-0.01 (-0.02, -0.01) $p=5\times10^{-7}$	0.58
Phospholipids (mmol/l)	-0.01 (-0.01, -0.01) $p=1\times10^{-10}$	-0.01 (-0.01, -4×10^{-3}) $p=3\times10^{-7}$	0.48
Total cholesterol (mmol/l)	-0.01 (-0.01, -4×10^{-3}) $p=7\times10^{-8}$	-0.01 (-0.01, -3×10^{-3}) $p=3\times10^{-6}$	0.70
Cholesterol esters (mmol/l)	-4×10^{-3} (-0.01, -3×10^{-3}) $p=4\times10^{-7}$	-4×10^{-3} (-0.01, -2×10^{-3}) $p=9\times10^{-6}$	0.73
Free cholesterol (mmol/l)	-2×10^{-3} $(-2\times10^{-3}, -1\times10^{-3})$ $p=5\times10^{-9}$	-2×10^{-3} $(-2\times10^{-3}, -9.6\times10^{-4})$ $p=7\times10^{-7}$	0.66
Triglycerides (mmol/l)	-2×10^{-4} $(-3\times10^{-4}, -2\times10^{-5})$ p=0.03	-1×10^{-4} $(-3\times10^{-4}, 2\times10^{-5})$ p=0.10	0.70
Large HDL			
Particle concentration (mol/l)	-3×10^{-8} $(-4\times10^{-8}, -2\times10^{-8})$ $p=4\times10^{-8}$	-2×10^{-8} $(-3\times10^{-8}, -1\times10^{-8})$ $p=5\times10^{-5}$	0.31
Total lipids (mmol/l)	-0.02 (-0.02, -0.01) $p=5\times10^{-9}$	-0.01 (-0.02, -0.01) $p=3\times10^{-5}$	0.26
Phospholipids (mmol/l)	-0.01 (-0.01, -5×10^{-3}) $p=5\times10^{-8}$	-0.01 (-0.01, -2×10^{-3}) $p=3\times10^{-4}$	0.21
Total cholesterol (mmol/l)	-0.01 (-0.01, -0.01) $p=3\times10^{-10}$	-0.01 (-0.01, -5×10^{-3}) $p=3\times10^{-6}$	0.27
Cholesterol esters (mmol/l)	-0.01 (-0.01, -0.01) $p=2\times10^{-10}$	-0.01 (-0.01, -4×10^{-3}) $p=4\times10^{-6}$	0.25
Free cholesterol (mmol/l)	-2×10^{-3} $(-3\times10^{-3}, -2\times10^{-3})$ $p=6\times10^{-10}$	-2×10^{-3} $(-3\times10^{-3}, -1\times10^{-3})$ $p=2\times10^{-6}$	0.36
Triglycerides (mmol/l)	-2×10^{-4} $(-5\times10^{-4}, 1\times10^{-4})$ p=0.26	-2×10^{-4} $(-5\times10^{-4}, 7\times10^{-5})$ p=0.13	0.76
Medium HDL			
Particle concentration (mol/l)	-2×10^{-9} $(-1\times10^{-8}, 7\times10^{-9})$ p=0.73	5×10^{-9} $(-4\times10^{-9}, 1\times10^{-8})$ p=0.25	0.27
Total lipids (mmol/l)	-1×10^{-3} (-0.01, 3×10^{-3}) p=0.52	2×10^{-3} $(-2\times10^{-3}, 0.01)$ p=0.34	0.23
Phospholipids (mmol/l)	-1×10^{-3} $(-3\times10^{-3}, 7\times10^{-4})$ p=0.25	8×10^{-4} $(-1\times10^{-3}, 3\times10^{-3})$ p=0.40	0.14
Total cholesterol (mmol/l)	-9.9×10^{-4} $(-3\times10^{-3}, 1\times10^{-3})$ p=0.38	6×10^{-4} $(-2\times10^{-3}, 3\times10^{-3})$ p=0.57	0.28
Cholesterol esters (mmol/l)	-8×10^{-4} $(-3\times10^{-3}, 9.9\times10^{-4})$ p=0.39	5×10^{-4} $(-1\times10^{-3}, 2\times10^{-3})$ p=0.58	0.29
Free cholesterol (mmol/l)	-2×10^{-4} $(-6\times10^{-4}, 2\times10^{-4})$ p=0.38	1×10^{-4} $(-3\times10^{-4}, 6\times10^{-4})$ p=0.55	0.28
Triglycerides (mmol/l)	7×10^{-4} $(4\times10^{-4}, 1\times10^{-3})$ $p=3\times10^{-6}$	6×10^{-4} $(3\times10^{-4}, 9\times10^{-4})$ $p=4\times10^{-4}$	0.42
Small HDL			

S3 Table. One-stage individual participant data (IPD) meta-analysis: offspring lipoproteins, lipids and metabolites absolute concentration differences per 1-SD higher parental BMI.

One stage-Individual Participant Data meta-analysis			
Metabolites	mother	father	P_{boot}
Particle concentration (mol/l)	1×10^{-8} (9×10^{-10}, 3×10^{-8}) p=0.04	3×10^{-8} (1×10^{-8}, 4×10^{-8}) $p=1\times10^{-4}$	0.15
Total lipids (mmol/l)	2×10^{-3} (-9.8×10^{-4}, 0.01) p=0.18	0.01 (3×10^{-3}, 0.01) $p=4\times10^{-4}$	0.07
Phospholipids (mmol/l)	2×10^{-3} (-2×10^{-4}, 3×10^{-3}) p=0.08	3×10^{-3} (1×10^{-3}, 0.01) $p=5\times10^{-4}$	0.16
Total cholesterol (mmol/l)	5×10^{-5} (-2×10^{-3}, 2×10^{-3}) p=0.96	2×10^{-3} (4×10^{-4}, 4×10^{-3}) p=0.02	0.07
Cholesterol esters (mmol/l)	2×10^{-4} (-1×10^{-3}, 2×10^{-3}) p=0.82	2×10^{-3} (2×10^{-4}, 3×10^{-3}) p=0.03	0.12
Free cholesterol (mmol/l)	-1×10^{-4} (-5×10^{-4}, 3×10^{-4}) p=0.56	4×10^{-4} (2×10^{-5}, 8×10^{-4}) p=0.04	0.04
Triglycerides (mmol/l)	5×10^{-4} (2×10^{-4}, 8×10^{-4}) $p=3\times10^{-4}$	6×10^{-4} (3×10^{-4}, 9×10^{-4}) $p=7\times10^{-5}$	0.70
**Lipoprotein particle size**			
VLDL particle size (nm)	0.08 (0.04, 0.11) $p=4\times10^{-6}$	0.06 (0.03, 0.10) $p=8\times10^{-5}$	0.61
LDL particle size (nm)	-3×10^{-3} (-0.01, 8×10^{-4}) p=0.13	-3×10^{-3} (-0.01, 4×10^{-4}) p=0.08	0.79
HDL particle size (nm)	-0.02 (-0.02, -0.01) $p=3\times10^{-11}$	-0.02 (-0.02, -0.01) $p=9\times10^{-10}$	0.87
**Cholesterol**			
Total cholesterol (mmol/l)	0.01 (-0.01, 0.03) p=0.54	5×10^{-3} (-0.02, 0.02) p=0.66	0.91
VLDL cholesterol (mmol/l)	0.01 (0.01, 0.02) $p=3\times10^{-6}$	0.01 (4×10^{-3}, 0.01) $p=5\times10^{-4}$	0.37
Remnant cholesterol (mmol/l)	0.02 (0.01, 0.02) $p=2\times10^{-4}$	0.01 (2×10^{-3}, 0.02) p=0.02	0.33
LDL cholesterol (mmol/l)	0.01 (-3×10^{-3}, 0.02) p=0.15	5×10^{-3} (-0.01, 0.02) p=0.38	0.70
HDL cholesterol (mmol/l)	-0.02 (-0.02, -0.01) $p=1\times10^{-7}$	-0.01 (-0.02, -4×10^{-3}) $p=2\times10^{-3}$	0.16
HDL_2 cholesterol (mmol/l)	-0.01 (-0.02, -0.01) $p=9\times10^{-7}$	-0.01 (-0.01, -4×10^{-3}) $p=1\times10^{-3}$	0.25
HDL_3 cholesterol (mmol/l)	-4×10^{-3} (-0.01, -2×10^{-3}) $p=2\times10^{-6}$	-2×10^{-3} (-3×10^{-3}, 8×10^{-5}) p=0.06	0.06
Esterified cholesterol (mmol/l)	4×10^{-3} (-0.01, 0.02) p=0.56	3×10^{-3} (-0.01, 0.02) p=0.69	0.90
Free cholesterol (mmol/l)	2×10^{-3} (-4×10^{-3}, 0.01) p=0.51	2×10^{-3} (-4×10^{-3}, 0.01) p=0.57	0.96
**Glycerides and phospholipids**			
Triglycerides (mmol/l)	0.02 (0.01, 0.03) $p=8\times10^{-6}$	0.02 (0.01, 0.03) $p=4\times10^{-5}$	0.82
VLDL triglycerides (mmol/l)	0.02 (0.01, 0.03) $p=7\times10^{-7}$	0.02 (0.01, 0.03) $p=9\times10^{-6}$	0.70
LDL triglycerides (mmol/l)	-7×10^{-5} (-1×10^{-3}, 1×10^{-3}) p=0.90	6×10^{-4} (-6×10^{-4}, 2×10^{-3}) p=0.34	0.41
HDL triglycerides (mmol/l)	9.5×10^{-4} (2×10^{-4}, 2×10^{-3}) p=0.02	7×10^{-4} (-7×10^{-5}, 2×10^{-3}) p=0.07	0.70
Phosphoglycerides (mmol/l)	-3×10^{-3} (-0.01, 0.01) p=0.49	2×10^{-3} (-0.01, 0.01) p=0.57	0.37
Phosphatidylcholine + other cholines (mmol/l)	-4×10^{-3} (-0.01, 4×10^{-3}) p=0.36	4×10^{-3} (-5×10^{-3}, 0.01) p=0.41	0.18
Cholines (mmol/l)	-4×10^{-3} (-0.01, 4×10^{-3}) p=0.32	1×10^{-3} (-0.01, 0.01) p=0.82	0.40
**Apolipoproteins**			
Apolipoprotein A-I (g/l)	-0.01 (-0.01, -3×10^{-3}) $p=2\times10^{-3}$	-3×10^{-3} (-0.01, 1×10^{-3}) p=0.15	0.23
Apolipoprotein B (g/l)	0.01 (4×10^{-3}, 0.01) $p=8\times10^{-5}$	0.01 (2×10^{-3}, 0.01) $p=4\times10^{-3}$	0.44
**Fatty acids**			
Total fatty acids (mmol/l)	0.05 (2×10^{-3}, 0.10) p=0.04	0.06 (0.01, 0.11) p=0.02	0.81
Degree of unsaturation	-1×10^{-3} (-3×10^{-3}, 4×10^{-4}) p=0.14	-3×10^{-4} (-2×10^{-3}, 1×10^{-3}) p=0.74	0.39
Docosahexaenoic acid (mmol/l)	-8×10^{-4} (-2×10^{-3}, 2×10^{-4}) p=0.11	9.8×10^{-4} (-1×10^{-4}, 2×10^{-3}) p=0.08	0.01
Linoleic acid (mmol/l)	3×10^{-3} (-0.01, 0.02) p=0.57	3×10^{-3} (-0.01, 0.02) p=0.62	0.97
n-3 fatty acids (mmol/l)	-2×10^{-4} (-3×10^{-3}, 2×10^{-3}) p=0.90	3×10^{-3} (3×10^{-5}, 0.01) p=0.05	0.12
n-6 fatty acids (mmol/l)	5×10^{-3} (-0.01, 0.02) p=0.53	0.01 (-0.01, 0.02) p=0.42	0.87
PUFA (mmol/l)	4×10^{-3} (-0.01, 0.02) p=0.61	0.01 (-0.01, 0.03) p=0.31	0.68
MUFA (mmol/l)	0.03 (0.01, 0.05) $p=4\times10^{-4}$	0.03 (0.01, 0.05) $p=4\times10^{-4}$	0.85
Saturated fatty acids (mmol/l)	0.01 (-0.01, 0.03) p=0.17	0.02 (-5×10^{-3}, 0.03) p=0.13	0.90
**Fatty acids ratios**			
Docosahexaenoic acid (%)	-0.01 (-0.02, -0.01) $p=2\times10^{-4}$	2×10^{-3} (-0.01, 0.01) p=0.66	3×10^{-3}
Linoleic acid (%)	-0.08 (-0.14, -0.02) p=0.01	-0.11 (-0.18, -0.04) $p=1\times10^{-3}$	0.50
n-3 fatty acids (%)	-0.02 (-0.04, -3×10^{-3}) p=0.02	0.01 (-0.01, 0.02) p=0.60	0.04
n-6 fatty acids (%)	-0.10 (-0.16, -0.03) $p=3\times10^{-3}$	-0.11 (-0.18, -0.05) $p=8\times10^{-4}$	0.70
PUFA (%)	-0.12 (-0.19, -0.05) $p=8\times10^{-4}$	-0.11 (-0.18, -0.04) $p=3\times10^{-3}$	0.82
MUFA (%)	0.17 (0.10, 0.25) $p=7\times10^{-6}$	0.18 (0.10, 0.25) $p=1\times10^{-5}$	0.99
Saturated fatty acids (%)	-0.05 (-0.11, 4×10^{-3}) p=0.07	-0.07 (-0.12, -0.01) p=0.03	0.74
**Glycolysis related metabolites**			
Glucose (mmol/l)	0.01 (-5×10^{-3}, 0.02) p=0.23	0.01 (-5×10^{-3}, 0.02) p=0.19	0.83
Lactate (mmol/l)	2×10^{-3} (-0.01, 0.01) p=0.76	-0.01 (-0.02, 4×10^{-3}) p=0.18	0.19

S3 Table. One-stage individual participant data (IPD) meta-analysis: offspring lipoproteins, lipids and metabolites absolute concentration differences per 1-SD higher parental BMI.

One stage-Individual Participant Data meta-analysis			
Metabolites	mother	father	P_{boot}
Pyruvate (mmol/l)	1×10^{-3} (4×10^{-4}, 2×10^{-3}) p=4×10^{-3}	3×10^{-4} (-5×10^{-4}, 1×10^{-3}) p=0.45	0.12
Citrate (mmol/l)	-9.7×10^{-4} (-2×10^{-3}, -4×10^{-4}) p=1×10^{-3}	-1×10^{-3} (-2×10^{-3}, -6×10^{-4}) p=7×10^{-5}	0.51
**Amino acids**			
Alanine (mmol/l)	2×10^{-4} (-1×10^{-3}, 2×10^{-3}) p=0.77	-3×10^{-4} (-2×10^{-3}, 1×10^{-3}) p=0.71	0.61
Glutamine (mmol/l)	-6×10^{-4} (-2×10^{-3}, 1×10^{-3}) p=0.54	-3×10^{-3} (-4×10^{-3}, -9×10^{-4}) p=4×10^{-3}	0.08
Histidine (mmol/l)	3×10^{-4} (-3×10^{-5}, 6×10^{-4}) p=0.08	3×10^{-4} (1×10^{-5}, 7×10^{-4}) p=0.04	0.77
Branched-chain amino acids			
Isoleucine (mmol/l)	8×10^{-4} (4×10^{-4}, 1×10^{-3}) p=9×10^{-6}	7×10^{-4} (4×10^{-4}, 1×10^{-3}) p=3×10^{-5}	0.83
Leucine (mmol/l)	5×10^{-4} (2×10^{-4}, 8×10^{-4}) p=2×10^{-3}	8×10^{-4} (4×10^{-4}, 1×10^{-3}) p=9×10^{-6}	0.24
Valine (mmol/l)	1×10^{-3} (6×10^{-4}, 2×10^{-3}) p=9.8×10^{-4}	2×10^{-3} (9.5×10^{-4}, 3×10^{-3}) p=4×10^{-5}	0.50
Aromatic amino acids			
Phenylalanine (mmol/l)	5×10^{-4} (3×10^{-4}, 7×10^{-4}) p=9×10^{-6}	3×10^{-4} (1×10^{-4}, 6×10^{-4}) p=3×10^{-3}	0.29
Tyrosine (mmol/l)	5×10^{-4} (2×10^{-4}, 8×10^{-4}) p=2×10^{-3}	4×10^{-4} (8×10^{-5}, 7×10^{-4}) p=0.01	0.65
**Ketone bodies**			
Acetate (mmol/l)	-8×10^{-4} (-1×10^{-3}, -3×10^{-4}) p=2×10^{-3}	-5×10^{-4} (-10×10^{-4}, 5×10^{-5}) p=0.07	0.25
Acetoacetate (mmol/l)	4×10^{-4} (-9.6×10^{-4}, 2×10^{-3}) p=0.55	4×10^{-4} (-8×10^{-4}, 2×10^{-3}) p=0.51	0.97
Beta-hydroxybutyrate (mmol/l)	-2×10^{-3} (-0.01, 5×10^{-4}) p=0.11	-5×10^{-4} (-4×10^{-3}, 3×10^{-3}) p=0.73	0.37
**Fluid balance**			
Creatinine (mmol/l)	3×10^{-4} (6×10^{-5}, 6×10^{-4}) p=0.01	5×10^{-4} (2×10^{-4}, 7×10^{-4}) p=2×10^{-4}	0.32
Albumin (signal area)	6×10^{-5} (-2×10^{-4}, 3×10^{-4}) p=0.62	5×10^{-5} (-2×10^{-4}, 3×10^{-4}) p=0.69	0.94
**Inflammation**			
Glycoprotein acetyls (mmol/l)	0.01 (0.01, 0.02) p=8×10^{-7}	0.01 (0.01, 0.02) p=2×10^{-5}	0.51

Associations were adjusted for parental age, smoking, education, head of household social class, maternal parity, offspring's age at blood collection, sex and cohort membership.
Categorical variables were harmonized across the three cohorts (see S1 Table).
S3 Table correspond to the SD-scaled metabolite concentration shown in Fig 2.
