# Supplementary material for: Association of pre-pregnancy body mass index with offspring metabolic profile: Analyses of 3 European prospective birth cohorts
Source: PLoS Med. 2017 Aug 22;14(8):e1002376. doi: 10.1371/journal.pmed.1002376 (PMC5568725; doi:10.1371/journal.pmed.1002376)
Supplement: S4 Table — (PDF) [file pmed.1002376.s011.pdf]

S4 Table. Two-stage IPD meta-analysis: offspring lipoproteins, lipids and metabolites absolute concentration differences per 1-SD higher parental BMI.

Two stage-Individual Participant Data meta-analysis				
Metabolites	mother	I^2_{mother}	father	I^2_{father}
**Lipoprotein subclasses**				
Extremely large VLDL				
Particle concentration (mol/l)	6×10^{-12} (3×10^{-12}, 8×10^{-12}) p=4×10^{-7}	10	4×10^{-12} (1×10^{-12}, 6×10^{-12}) p=3×10^{-3}	17
Total lipids (mmol/l)	1×10^{-3} (7×10^{-4}, 2×10^{-3}) p=2×10^{-6}	14	8×10^{-4} (2×10^{-4}, 1×10^{-3}) p=0.00	20
Phospholipids (mmol/l)	1×10^{-4} (8×10^{-5}, 2×10^{-4}) p=2×10^{-6}	9	1×10^{-4} (4×10^{-5}, 2×10^{-4}) p=1×10^{-3}	0
Total cholesterol (mmol/l)	2×10^{-4} (1×10^{-4}, 3×10^{-4}) p=2×10^{-7}	5	2×10^{-4} (4×10^{-5}, 3×10^{-4}) p=0.01	30
Cholesterol esters (mmol/l)	1×10^{-4} (9×10^{-5}, 2×10^{-4}) p=4×10^{-8}	0	8×10^{-5} (1×10^{-5}, 2×10^{-4}) p=0.02	42
Free cholesterol (mmol/l)	9×10^{-5} (5×10^{-5}, 1×10^{-4}) p=3×10^{-6}	14	7×10^{-5} (3×10^{-5}, 1×10^{-4}) p=9.9×10^{-4}	0
Triglycerides (mmol/l)	8×10^{-4} (5×10^{-4}, 1×10^{-3}) p=3×10^{-6}	17	6×10^{-4} (2×10^{-4}, 9.5×10^{-4}) p=0.01	21
Very large VLDL				
Particle concentration (mol/l)	3×10^{-11} (2×10^{-11}, 4×10^{-11}) p=1×10^{-7}	0	3×10^{-11} (1×10^{-11}, 4×10^{-11}) p=1×10^{-4}	0
Total lipids (mmol/l)	3×10^{-3} (2×10^{-3}, 4×10^{-3}) p=1×10^{-7}	0	2×10^{-3} (1×10^{-3}, 4×10^{-3}) p=1×10^{-4}	0
Phospholipids (mmol/l)	5×10^{-4} (3×10^{-4}, 7×10^{-4}) p=1×10^{-7}	0	4×10^{-4} (2×10^{-4}, 6×10^{-4}) p=1×10^{-4}	0
Total cholesterol (mmol/l)	7×10^{-4} (4×10^{-4}, 9×10^{-4}) p=3×10^{-7}	9	5×10^{-4} (2×10^{-4}, 7×10^{-4}) p=5×10^{-4}	1
Cholesterol esters (mmol/l)	4×10^{-4} (2×10^{-4}, 5×10^{-4}) p=1×10^{-6}	19	2×10^{-4} (8×10^{-5}, 4×10^{-4}) p=4×10^{-3}	26
Free cholesterol (mmol/l)	3×10^{-4} (2×10^{-4}, 4×10^{-4}) p=1×10^{-7}	0	2×10^{-4} (1×10^{-4}, 4×10^{-4}) p=4×10^{-4}	0
Triglycerides (mmol/l)	2×10^{-3} (1×10^{-3}, 3×10^{-3}) p=2×10^{-7}	0	2×10^{-3} (8×10^{-4}, 2×10^{-3}) p=8×10^{-5}	0
Large VLDL				
Particle concentration (mol/l)	2×10^{-10} (1×10^{-10}, 2×10^{-10}) p=1×10^{-7}	0	1×10^{-10} (8×10^{-11}, 2×10^{-10}) p=2×10^{-5}	0
Total lipids (mmol/l)	0.01 (0.01, 0.01) p=9×10^{-8}	0	0.01 (5×10^{-3}, 0.01) p=2×10^{-5}	0
Phospholipids (mmol/l)	2×10^{-3} (1×10^{-3}, 2×10^{-3}) p=9×10^{-8}	0	2×10^{-3} (8×10^{-4}, 2×10^{-3}) p=2×10^{-5}	0
Total cholesterol (mmol/l)	2×10^{-3} (2×10^{-3}, 3×10^{-3}) p=3×10^{-8}	0	2×10^{-3} (9×10^{-4}, 3×10^{-3}) p=2×10^{-4}	9
Cholesterol esters (mmol/l)	1×10^{-3} (8×10^{-4}, 2×10^{-3}) p=1×10^{-8}	0	9×10^{-4} (3×10^{-4}, 1×10^{-3}) p=3×10^{-3}	32
Free cholesterol (mmol/l)	1×10^{-3} (7×10^{-4}, 2×10^{-3}) p=1×10^{-7}	0	9.8×10^{-4} (5×10^{-4}, 1×10^{-3}) p=4×10^{-5}	0
Triglycerides (mmol/l)	0.01 (3×10^{-3}, 0.01) p=2×10^{-7}	0	0.01 (3×10^{-3}, 0.01) p=1×10^{-5}	0
Medium VLDL				
Particle concentration (mol/l)	4×10^{-10} (3×10^{-10}, 6×10^{-10}) p=3×10^{-8}	0	4×10^{-10} (2×10^{-10}, 5×10^{-10}) p=7×10^{-6}	0
Total lipids (mmol/l)	0.01 (0.01, 0.02) p=3×10^{-8}	0	0.01 (0.01, 0.02) p=9×10^{-6}	0
Phospholipids (mmol/l)	3×10^{-3} (2×10^{-3}, 4×10^{-3}) p=5×10^{-8}	0	2×10^{-3} (1×10^{-3}, 3×10^{-3}) p=1×10^{-5}	0
Total cholesterol (mmol/l)	4×10^{-3} (2×10^{-3}, 0.01) p=4×10^{-8}	0	3×10^{-3} (2×10^{-3}, 5×10^{-3}) p=8×10^{-5}	0
Cholesterol esters (mmol/l)	2×10^{-3} (1×10^{-3}, 3×10^{-3}) p=3×10^{-8}	0	2×10^{-3} (6×10^{-4}, 3×10^{-3}) p=1×10^{-3}	17
Free cholesterol (mmol/l)	2×10^{-3} (1×10^{-3}, 2×10^{-3}) p=2×10^{-7}	0	2×10^{-3} (8×10^{-4}, 2×10^{-3}) p=2×10^{-5}	0
Triglycerides (mmol/l)	0.01 (5×10^{-3}, 0.01) p=5×10^{-8}	0	0.01 (4×10^{-3}, 0.01) p=4×10^{-6}	0
Small VLDL				
Particle concentration (mol/l)	4×10^{-10} (3×10^{-10}, 6×10^{-10}) p=1×10^{-7}	0	4×10^{-10} (2×10^{-10}, 6×10^{-10}) p=1×10^{-5}	0
Total lipids (mmol/l)	0.01 (0.01, 0.01) p=2×10^{-7}	0	0.01 (4×10^{-3}, 0.01) p=3×10^{-5}	0
Phospholipids (mmol/l)	2×10^{-3} (1×10^{-3}, 3×10^{-3}) p=4×10^{-7}	0	2×10^{-3} (9.9×10^{-4}, 3×10^{-3}) p=1×10^{-5}	0
Total cholesterol (mmol/l)	3×10^{-3} (2×10^{-3}, 5×10^{-3}) p=1×10^{-6}	0	3×10^{-3} (1×10^{-3}, 4×10^{-3}) p=1×10^{-3}	0
Cholesterol esters (mmol/l)	2×10^{-3} (1×10^{-3}, 3×10^{-3}) p=2×10^{-6}	0	1×10^{-3} (4×10^{-4}, 3×10^{-3}) p=0.01	0
Free cholesterol (mmol/l)	1×10^{-3} (6×10^{-4}, 2×10^{-3}) p=4×10^{-6}	0	1×10^{-3} (6×10^{-4}, 2×10^{-3}) p=7×10^{-5}	0
Triglycerides (mmol/l)	4×10^{-3} (2×10^{-3}, 0.01) p=4×10^{-7}	0	4×10^{-3} (2×10^{-3}, 0.01) p=9×10^{-6}	0
Very Small VLDL				
Particle concentration (mol/l)	3×10^{-10} (9×10^{-11}, 4×10^{-10}) p=2×10^{-3}	0	2×10^{-10} (3×10^{-11}, 4×10^{-10}) p=0.02	0
Total lipids (mmol/l)	4×10^{-3} (2×10^{-3}, 0.01) p=6×10^{-4}	0	3×10^{-3} (-3×10^{-6}, 0.01) p=0.05	0
Phospholipids (mmol/l)	9×10^{-4} (2×10^{-4}, 2×10^{-3}) p=0.01	0	6×10^{-4} (-2×10^{-4}, 1×10^{-3}) p=0.16	0
Total cholesterol (mmol/l)	2×10^{-3} (9×10^{-4}, 3×10^{-3}) p=9×10^{-4}	0	9×10^{-4} (-6×10^{-4}, 2×10^{-3}) p=0.22	0
Cholesterol esters (mmol/l)	2×10^{-3} (9×10^{-4}, 3×10^{-3}) p=1×10^{-4}	0	7×10^{-4} (-4×10^{-4}, 2×10^{-3}) p=0.22	0
Free cholesterol (mmol/l)	4×10^{-4} (-2×10^{-5}, 8×10^{-4}) p=0.07	0	3×10^{-4} (-2×10^{-4}, 7×10^{-4}) p=0.27	0
Triglycerides (mmol/l)	9.9×10^{-4} (4×10^{-4}, 2×10^{-3}) p=4×10^{-4}	0	1×10^{-3} (5×10^{-4}, 2×10^{-3}) p=5×10^{-4}	0
IDL				
Particle concentration (mol/l)	4×10^{-10} (-7×10^{-11}, 8×10^{-10}) p=0.10	0	1×10^{-10} (-4×10^{-10}, 6×10^{-10}) p=0.69	0
Total lipids (mmol/l)	5×10^{-3} (-1×10^{-4}, 0.01) p=0.06	0	2×10^{-3} (-4×10^{-3}, 0.01) p=0.61	0
Phospholipids (mmol/l)	1×10^{-3} (-3×10^{-4}, 2×10^{-3}) p=0.12	0	2×10^{-4} (-1×10^{-3}, 2×10^{-3}) p=0.81	0
Total cholesterol (mmol/l)	4×10^{-3} (4×10^{-4}, 0.01) p=0.03	0	9.9×10^{-4} (-3×10^{-3}, 0.01) p=0.63	0

S4 Table. Two-stage IPD meta-analysis: offspring lipoproteins, lipids and metabolites absolute concentration differences per 1-SD higher parental BMI.

**Two stage-Individual Participant Data meta-analysis**				
Metabolites	mother	I^2_{mother}	father	I^2_{father}
Cholesterol esters (mmol/l)	3×10^{-3} (9×10^{-4}, 0.01) p=0.01	0	1×10^{-3} (-2×10^{-3}, 4×10^{-3}) p=0.44	0
Free cholesterol (mmol/l)	5×10^{-4} (-5×10^{-4}, 2×10^{-3}) p=0.34	0	-2×10^{-4} (-1×10^{-3}, 9.9×10^{-4}) p=0.78	0
Triglycerides (mmol/l)	2×10^{-4} (-4×10^{-4}, 8×10^{-4}) p=0.46	0	4×10^{-4} (-2×10^{-4}, 1×10^{-3}) p=0.18	0
Large LDL				
Particle concentration (mol/l)	7×10^{-10} (-3×10^{-11}, 1×10^{-9}) p=0.06	0	3×10^{-10} (-5×10^{-10}, 1×10^{-9}) p=0.45	0
Total lipids (mmol/l)	0.01 (-1×10^{-4}, 0.01) p=0.05	0	3×10^{-3} (-4×10^{-3}, 0.01) p=0.44	0
Phospholipids (mmol/l)	1×10^{-3} (2×10^{-4}, 3×10^{-3}) p=0.02	0	8×10^{-4} (-7×10^{-4}, 2×10^{-3}) p=0.31	0
Total cholesterol (mmol/l)	5×10^{-3} (1×10^{-4}, 0.01) p=0.04	0	2×10^{-3} (-4×10^{-3}, 0.01) p=0.50	0
Cholesterol esters (mmol/l)	4×10^{-3} (5×10^{-4}, 0.01) p=0.02	0	2×10^{-3} (-2×10^{-3}, 0.01) p=0.39	0
Free cholesterol (mmol/l)	7×10^{-4} (-4×10^{-4}, 2×10^{-3}) p=0.22	0	5×10^{-5} (-1×10^{-3}, 1×10^{-3}) p=0.94	0
Triglycerides (mmol/l)	-1×10^{-5} (-6×10^{-4}, 6×10^{-4}) p=0.97	0	2×10^{-4} (-4×10^{-4}, 9×10^{-4}) p=0.53	0
Medium LDL				
Particle concentration (mol/l)	7×10^{-10} (9×10^{-11}, 1×10^{-9}) p=0.02	0	4×10^{-10} (-3×10^{-10}, 1×10^{-9}) p=0.27	0
Total lipids (mmol/l)	4×10^{-3} (4×10^{-4}, 0.01) p=0.03	0	2×10^{-3} (-2×10^{-3}, 0.01) p=0.31	0
Phospholipids (mmol/l)	1×10^{-3} (4×10^{-4}, 2×10^{-3}) p=4×10^{-3}	0	7×10^{-4} (-2×10^{-4}, 2×10^{-3}) p=0.14	0
Total cholesterol (mmol/l)	3×10^{-3} (2×10^{-4}, 0.01) p=0.04	0	1×10^{-3} (-2×10^{-3}, 5×10^{-3}) p=0.38	0
Cholesterol esters (mmol/l)	3×10^{-3} (2×10^{-4}, 5×10^{-3}) p=0.03	0	1×10^{-3} (-1×10^{-3}, 4×10^{-3}) p=0.35	0
Free cholesterol (mmol/l)	5×10^{-4} (-4×10^{-5}, 1×10^{-3}) p=0.07	0	2×10^{-4} (-5×10^{-4}, 8×10^{-4}) p=0.58	0
Triglycerides (mmol/l)	-3×10^{-5} (-3×10^{-4}, 3×10^{-4}) p=0.88	0	8×10^{-5} (-3×10^{-4}, 5×10^{-4}) p=0.66	7
Small LDL				
Particle concentration (mol/l)	8×10^{-10} (6×10^{-11}, 1×10^{-9}) p=0.03	0	4×10^{-10} (-4×10^{-10}, 1×10^{-9}) p=0.30	0
Total lipids (mmol/l)	2×10^{-3} (1×10^{-4}, 5×10^{-3}) p=0.04	0	1×10^{-3} (-1×10^{-3}, 4×10^{-3}) p=0.30	0
Phospholipids (mmol/l)	6×10^{-4} (1×10^{-4}, 1×10^{-3}) p=0.02	0	4×10^{-4} (-2×10^{-4}, 1×10^{-3}) p=0.17	0
Total cholesterol (mmol/l)	2×10^{-3} (-6×10^{-5}, 3×10^{-3}) p=0.06	0	8×10^{-4} (-1×10^{-3}, 3×10^{-3}) p=0.44	0
Cholesterol esters (mmol/l)	1×10^{-3} (-3×10^{-5}, 3×10^{-3}) p=0.06	0	7×10^{-4} (-9.8×10^{-4}, 2×10^{-3}) p=0.42	0
Free cholesterol (mmol/l)	3×10^{-4} (-5×10^{-5}, 6×10^{-4}) p=0.10	0	1×10^{-4} (-3×10^{-4}, 5×10^{-4}) p=0.59	0
Triglycerides (mmol/l)	2×10^{-4} (-3×10^{-5}, 4×10^{-4}) p=0.10	0	2×10^{-4} (-8×10^{-5}, 5×10^{-4}) p=0.16	39
Very large HDL				
Particle concentration (mol/l)	-9.9×10^{-9} (-1×10^{-8}, -7×10^{-9}) p=6×10^{-10}	0	-9×10^{-9} (-1×10^{-8}, -5×10^{-9}) p=2×10^{-6}	0
Total lipids (mmol/l)	-0.01 (-0.01, -0.01) p=1×10^{-7}	20	-0.01 (-0.02, -0.01) p=8×10^{-7}	0
Phospholipids (mmol/l)	-0.01 (-0.01, -4×10^{-3}) p=3×10^{-11}	0	-0.01 (-0.01, -3×10^{-3}) p=1×10^{-6}	0
Total cholesterol (mmol/l)	-4×10^{-3} (-0.01, -2×10^{-3}) p=1×10^{-3}	53	-0.01 (-0.01, -3×10^{-3}) p=2×10^{-6}	0
Cholesterol esters (mmol/l)	-3×10^{-3} (-0.01, -9×10^{-4}) p=0.01	60	-4×10^{-3} (-0.01, -2×10^{-3}) p=3×10^{-6}	0
Free cholesterol (mmol/l)	-1×10^{-3} (-2×10^{-3}, -9×10^{-4}) p=9×10^{-8}	13	-1×10^{-3} (-2×10^{-3}, -9×10^{-4}) p=1×10^{-6}	0
Triglycerides (mmol/l)	-1×10^{-4} (-3×10^{-4}, 7×10^{-7}) p=0.05	0	-1×10^{-4} (-3×10^{-4}, 4×10^{-5}) p=0.16	0
Large HDL				
Particle concentration (mol/l)	-2×10^{-8} (-3×10^{-8}, -2×10^{-8}) p=2×10^{-10}	0	-2×10^{-8} (-3×10^{-8}, -6×10^{-9}) p=1×10^{-3}	25
Total lipids (mmol/l)	-0.02 (-0.02, -0.01) p=1×10^{-10}	0	-0.01 (-0.02, -0.01) p=9×10^{-5}	0
Phospholipids (mmol/l)	-0.01 (-0.01, -5×10^{-3}) p=3×10^{-9}	0	-4×10^{-3} (-0.01, -2×10^{-3}) p=7×10^{-4}	0
Total cholesterol (mmol/l)	-0.01 (-0.01, -0.01) p=4×10^{-12}	0	-0.01 (-0.01, -4×10^{-3}) p=1×10^{-5}	0
Cholesterol esters (mmol/l)	-0.01 (-0.01, -0.01) p=4×10^{-12}	0	-0.01 (-0.01, -3×10^{-3}) p=1×10^{-5}	0
Free cholesterol (mmol/l)	-2×10^{-3} (-3×10^{-3}, -2×10^{-3}) p=8×10^{-12}	0	-2×10^{-3} (-3×10^{-3}, -7×10^{-4}) p=6×10^{-4}	37
Triglycerides (mmol/l)	-1×10^{-4} (-4×10^{-4}, 1×10^{-4}) p=0.28	26	-2×10^{-4} (-7×10^{-4}, 3×10^{-4}) p=0.50	69
Medium HDL				
Particle concentration (mol/l)	-1×10^{-9} (-8×10^{-9}, 6×10^{-9}) p=0.77	0	6×10^{-9} (-2×10^{-9}, 1×10^{-8}) p=0.12	0
Total lipids (mmol/l)	-1×10^{-3} (-4×10^{-3}, 2×10^{-3}) p=0.47	0	2×10^{-3} (-1×10^{-3}, 0.01) p=0.23	0
Phospholipids (mmol/l)	-9.9×10^{-4} (-3×10^{-3}, 5×10^{-4}) p=0.21	0	9×10^{-4} (-8×10^{-4}, 3×10^{-3}) p=0.30	0
Total cholesterol (mmol/l)	-9×10^{-4} (-3×10^{-3}, 8×10^{-4}) p=0.29	0	6×10^{-4} (-1×10^{-3}, 2×10^{-3}) p=0.53	0
Cholesterol esters (mmol/l)	-8×10^{-4} (-2×10^{-3}, 6×10^{-4}) p=0.27	0	4×10^{-4} (-1×10^{-3}, 2×10^{-3}) p=0.57	0
Free cholesterol (mmol/l)	-1×10^{-4} (-5×10^{-4}, 2×10^{-4}) p=0.45	14	1×10^{-4} (-2×10^{-4}, 5×10^{-4}) p=0.38	0
Triglycerides (mmol/l)	7×10^{-4} (4×10^{-4}, 9×10^{-4}) p=2×10^{-7}	0	6×10^{-4} (1×10^{-4}, 1×10^{-3}) p=0.02	60
Small HDL				
Particle concentration (mol/l)	1×10^{-8} (4×10^{-9}, 2×10^{-8}) p=0.01	2	2×10^{-8} (1×10^{-8}, 3×10^{-8}) p=4×10^{-5}	0
Total lipids (mmol/l)	2×10^{-3} (-4×10^{-4}, 5×10^{-3}) p=0.10	0	0.01 (3×10^{-3}, 0.01) p=3×10^{-4}	0

S4 Table. Two-stage IPD meta-analysis: offspring lipoproteins, lipids and metabolites absolute concentration differences per 1-SD higher parental BMI.

Two stage-Individual Participant Data meta-analysis				
Metabolites	mother	I^2_{mother}	father	I^2_{father}
Phospholipids (mmol/l)	1×10^{-3} (-6×10^{-4}, 3×10^{-3}) $p=0.18$	31	3×10^{-3} (1×10^{-3}, 5×10^{-3}) $p=3\times10^{-4}$	0
Total cholesterol (mmol/l)	6×10^{-5} (-1×10^{-3}, 1×10^{-3}) $p=0.93$	0	2×10^{-3} (1×10^{-4}, 3×10^{-3}) $p=0.04$	0
Cholesterol esters (mmol/l)	1×10^{-4} (-9.9×10^{-4}, 1×10^{-3}) $p=0.83$	0	1×10^{-3} (-3×10^{-5}, 3×10^{-3}) $p=0.06$	0
Free cholesterol (mmol/l)	-8×10^{-5} (-4×10^{-4}, 3×10^{-4}) $p=0.66$	0	4×10^{-4} (1×10^{-5}, 8×10^{-4}) $p=0.04$	0
Triglycerides (mmol/l)	5×10^{-4} (3×10^{-4}, 8×10^{-4}) $p=5\times10^{-5}$	0	6×10^{-4} (3×10^{-4}, 9×10^{-4}) $p=7\times10^{-5}$	9
**Lipoprotein particle size**				
VLDL particle size (nm)	0.06 (0.02, 0.09) $p=3\times10^{-3}$	59	0.06 (0.03, 0.09) $p=1\times10^{-4}$	0
LDL particle size (nm)	-2×10^{-3} (-0.01, 3×10^{-3}) $p=0.36$	69	-3×10^{-3} (-0.01, 6×10^{-4}) $p=0.10$	0
HDL particle size (nm)	-0.01 (-0.02, -0.01) $p=1\times10^{-12}$	0	-0.01 (-0.02, -0.01) $p=7\times10^{-9}$	0
**Cholesterol**				
Total cholesterol (mmol/l)	0.01 (-0.01, 0.03) $p=0.26$	0	4×10^{-3} (-0.02, 0.02) $p=0.70$	0
VLDL cholesterol (mmol/l)	0.01 (0.01, 0.02) $p=5\times10^{-8}$	0	0.01 (4×10^{-3}, 0.01) $p=6\times10^{-4}$	0
Remnant cholesterol (mmol/l)	0.02 (0.01, 0.02) $p=1\times10^{-5}$	0	0.01 (2×10^{-3}, 0.02) $p=0.02$	0
LDL cholesterol (mmol/l)	0.01 (2×10^{-5}, 0.02) $p=0.05$	0	4×10^{-3} (-0.01, 0.01) $p=0.45$	0
HDL cholesterol (mmol/l)	-0.02 (-0.02, -0.01) $p=7\times10^{-8}$	0	-0.01 (-0.02, -3×10^{-3}) $p=3\times10^{-3}$	0
HDL_2 cholesterol (mmol/l)	-0.01 (-0.02, -0.01) $p=2\times10^{-8}$	0	-0.01 (-0.01, -3×10^{-3}) $p=1\times10^{-3}$	0
HDL_3 cholesterol (mmol/l)	-2×10^{-3} (-3×10^{-3}, 9×10^{-5}) $p=0.06$	63	-9×10^{-4} (-3×10^{-3}, 1×10^{-3}) $p=0.46$	55
Esterified cholesterol (mmol/l)	0.01 (-5×10^{-3}, 0.02) $p=0.23$	0	2×10^{-3} (-0.01, 0.02) $p=0.76$	0
Free cholesterol (mmol/l)	3×10^{-3} (-2×10^{-3}, 0.01) $p=0.28$	0	2×10^{-3} (-4×10^{-3}, 0.01) $p=0.61$	0
**Glycerides and phospholipids**				
Triglycerides (mmol/l)	0.02 (0.01, 0.03) $p=2\times10^{-6}$	0	0.02 (0.01, 0.03) $p=4\times10^{-5}$	0
VLDL triglycerides (mmol/l)	0.02 (0.01, 0.03) $p=1\times10^{-7}$	0	0.02 (0.01, 0.03) $p=8\times10^{-6}$	0
LDL triglycerides (mmol/l)	1×10^{-4} (-9.5×10^{-4}, 1×10^{-3}) $p=0.83$	0	5×10^{-4} (-8×10^{-4}, 2×10^{-3}) $p=0.47$	15
HDL triglycerides (mmol/l)	9.5×10^{-4} (3×10^{-4}, 2×10^{-3}) $p=0.01$	0	8×10^{-4} (-6×10^{-4}, 2×10^{-3}) $p=0.27$	69
Phosphoglycerides (mmol/l)	9×10^{-5} (-0.01, 0.01) $p=0.98$	0	3×10^{-3} (-0.01, 0.01) $p=0.47$	0
Phosphatidylcholine + other cholines (mmol/l)	-1×10^{-3} (-0.01, 0.01) $p=0.77$	0	4×10^{-3} (-4×10^{-3}, 0.01) $p=0.34$	0
Cholines (mmol/l)	-1×10^{-3} (-0.01, 0.01) $p=0.72$	0	1×10^{-3} (-0.01, 0.01) $p=0.76$	0
**Apolipoproteins**				
Apolipoprotein A-I (g/l)	-0.01 (-0.01, -2×10^{-3}) $p=1\times10^{-3}$	0	-3×10^{-3} (-0.01, 1×10^{-3}) $p=0.17$	0
Apolipoprotein B (g/l)	0.01 (4×10^{-3}, 0.01) $p=1\times10^{-5}$	0	0.01 (2×10^{-3}, 0.01) $p=0.01$	0
**Fatty acids**				
Total fatty acids (mmol/l)	0.05 (0.01, 0.10) $p=0.01$	0	0.06 (0.01, 0.11) $p=0.02$	0
Degree of unsaturation	-6×10^{-4} (-2×10^{-3}, 5×10^{-4}) $p=0.29$	0	7×10^{-5} (-2×10^{-3}, 2×10^{-3}) $p=0.94$	37
Docosahexaenoic acid (mmol/l)	-9×10^{-5} (-1×10^{-3}, 1×10^{-3}) $p=0.90$	53	1×10^{-3} (9×10^{-5}, 2×10^{-3}) $p=0.03$	0
Linoleic acid (mmol/l)	3×10^{-3} (-0.01, 0.02) $p=0.64$	48	3×10^{-3} (-0.01, 0.02) $p=0.63$	0
n-3 fatty acids (mmol/l)	6×10^{-4} (-2×10^{-3}, 3×10^{-3}) $p=0.60$	0	3×10^{-3} (-5×10^{-4}, 0.01) $p=0.10$	36
n-6 fatty acids (mmol/l)	0.01 (-0.01, 0.02) $p=0.34$	18	0.01 (-0.01, 0.02) $p=0.43$	0
PUFA (mmol/l)	0.01 (-0.01, 0.02) $p=0.27$	0	0.01 (-0.01, 0.02) $p=0.31$	0
MUFA (mmol/l)	0.03 (0.01, 0.04) $p=2\times10^{-4}$	0	0.03 (0.01, 0.05) $p=0.01$	38
Saturated fatty acids (mmol/l)	0.02 (6×10^{-4}, 0.03) $p=0.04$	0	0.02 (-3×10^{-3}, 0.04) $p=0.09$	0
**Fatty acids ratios**				
Docosahexaenoic acid (%)	-0.01 (-0.02, 0.01) $p=0.44$	80	5×10^{-3} (-4×10^{-3}, 0.01) $p=0.26$	14
Linoleic acid (%)	-0.09 (-0.15, -0.04) $p=6\times10^{-4}$	14	-0.10 (-0.26, 0.06) $p=0.21$	83
n-3 fatty acids (%)	-0.01 (-0.03, 0.01) $p=0.28$	37	0.01 (-0.02, 0.04) $p=0.59$	68
n-6 fatty acids (%)	-0.10 (-0.15, -0.05) $p=2\times10^{-4}$	0	-0.11 (-0.27, 0.05) $p=0.19$	83
PUFA (%)	-0.11 (-0.16, -0.05) $p=2\times10^{-4}$	0	-0.10 (-0.29, 0.10) $p=0.33$	87
MUFA (%)	0.11 (0.05, 0.17) $p=2\times10^{-4}$	13	0.15 (-0.01, 0.31) $p=0.07$	77
Saturated fatty acids (%)	-0.01 (-0.08, 0.06) $p=0.75$	63	-0.06 (-0.11, 5×10^{-4}) $p=0.05$	0
**Glycolysis related metabolites**				
Glucose (mmol/l)	5×10^{-3} (-0.01, 0.02) $p=0.58$	42	0.01 (-0.01, 0.02) $p=0.30$	0
Lactate (mmol/l)	2×10^{-3} (-0.01, 0.01) $p=0.67$	0	-0.01 (-0.02, 4×10^{-3}) $p=0.25$	0
Pyruvate (mmol/l)	6×10^{-4} (-4×10^{-4}, 2×10^{-3}) $p=0.26$	72	2×10^{-5} (-8×10^{-4}, 9×10^{-4}) $p=0.96$	32
Citrate (mmol/l)	-8×10^{-4} (-1×10^{-3}, -4×10^{-5}) $p=0.04$	65	-1×10^{-3} (-2×10^{-3}, -6×10^{-4}) $p=2\times10^{-4}$	0
**Amino acids**				

S4 Table. Two-stage IPD meta-analysis: offspring lipoproteins, lipids and metabolites absolute concentration differences per 1-SD higher parental BMI.

**Two stage-Individual Participant Data meta-analysis**				
Metabolites	mother	I^2_{mother}	father	I^2_{father}
Alanine (mmol/l)	5×10^{-4} (-8×10^{-4}, 2×10^{-3}) p=0.47	0	-6×10^{-4} (-2×10^{-3}, 1×10^{-3}) p=0.48	0
Glutamine (mmol/l)	-1×10^{-3} (-3×10^{-3}, 6×10^{-4}) p=0.20	0	-2×10^{-3} (-4×10^{-3}, -7×10^{-4}) p=0.00	0
Histidine (mmol/l)	1×10^{-4} (-1×10^{-4}, 4×10^{-4}) p=0.34	22	3×10^{-4} (-3×10^{-5}, 6×10^{-4}) p=0.08	0
Branched-chain amino acids				
Isoleucine (mmol/l)	7×10^{-4} (4×10^{-4}, 1×10^{-3}) $p=5\times10^{-6}$	23	7×10^{-4} (4×10^{-4}, 1×10^{-3}) $p=2\times10^{-5}$	0
Leucine (mmol/l)	6×10^{-4} (3×10^{-4}, 9×10^{-4}) $p=1\times10^{-5}$	13	7×10^{-4} (4×10^{-5}, 1×10^{-3}) p=0.04	77
Valine (mmol/l)	2×10^{-3} (9×10^{-4}, 2×10^{-3}) $p=5\times10^{-5}$	24	2×10^{-3} (9.7×10^{-4}, 3×10^{-3}) $p=3\times10^{-5}$	0
Aromatic amino acids				
Phenylalanine (mmol/l)	7×10^{-4} (2×10^{-4}, 1×10^{-3}) p=0.01	84	3×10^{-4} (-8×10^{-5}, 6×10^{-4}) p=0.13	60
Tyrosine (mmol/l)	5×10^{-4} (1×10^{-4}, 8×10^{-4}) p=0.01	48	4×10^{-4} (5×10^{-5}, 7×10^{-4}) p=0.02	0
**Ketone bodies**				
Acetate (mmol/l)	-5×10^{-4} (-1×10^{-3}, 3×10^{-4}) p=0.24	80	-3×10^{-4} (-1×10^{-3}, 4×10^{-4}) p=0.41	57
Acetoacetate (mmol/l)	-5×10^{-4} (-2×10^{-3}, 1×10^{-3}) p=0.56	76	3×10^{-4} (-8×10^{-4}, 1×10^{-3}) p=0.58	0
Beta-hydroxybutyrate (mmol/l)	-2×10^{-3} (-0.01, 2×10^{-3}) p=0.25	70	-2×10^{-4} (-3×10^{-3}, 3×10^{-3}) p=0.92	7
**Fluid balance**				
Creatinine (mmol/l)	2×10^{-4} (-1×10^{-5}, 4×10^{-4}) p=0.06	0	4×10^{-4} (2×10^{-4}, 7×10^{-4}) $p=8\times10^{-4}$	0
Albumin (signal area)	-8×10^{-5} (-3×10^{-4}, 1×10^{-4}) p=0.38	15	-6×10^{-6} (-2×10^{-4}, 2×10^{-4}) p=0.94	0
**Inflammation**				
Glycoprotein acetyls (mmol/l)	0.01 (0.01, 0.02) $p=7\times10^{-8}$	0	0.01 (5×10^{-3}, 0.01) $p=4\times10^{-5}$	0

Associations were adjusted for parental age, smoking, education, head of household social class, maternal parity, offspring's age at blood collection and sex.
I^2 statistics indicates between-cohort heterogeneity (very low $\leq$ 50%; substantial=50-75%; very high $\geq$ 75%).
S4 Table correspond to the SD-scaled metabolite concentration shown in S3 Fig.
