# Supplementary material for: Association of pre-pregnancy body mass index with offspring metabolic profile: Analyses of 3 European prospective birth cohorts
Source: PLoS Med. 2017 Aug 22;14(8):e1002376. doi: 10.1371/journal.pmed.1002376 (PMC5568725; doi:10.1371/journal.pmed.1002376)
Supplement: S5 Table — (PDF) [file pmed.1002376.s012.pdf]

S5 Table. ALSPAC: offspring lipoproteins, lipids and metabolites absolute concentration differences per 1-SD higher parental BMI.

Avon Longitudinal Study of Parents and Children (ALSPAC)			
Metabolites	mother	father	P_{boot}
**Lipoprotein subclasses**			
Extremely large VLDL			
Particle concentration (mol/l)	7×10^{-12} (4×10^{-12}, 10×10^{-12}) p=2×10^{-5}	5×10^{-12} (2×10^{-12}, 8×10^{-12}) p=2×10^{-3}	0.37
Total lipids (mmol/l)	1×10^{-3} (8×10^{-4}, 2×10^{-3}) p=3×10^{-5}	1×10^{-3} (4×10^{-4}, 2×10^{-3}) p=3×10^{-3}	0.38
Phospholipids (mmol/l)	2×10^{-4} (9×10^{-5}, 3×10^{-4}) p=5×10^{-5}	1×10^{-4} (4×10^{-5}, 2×10^{-4}) p=3×10^{-3}	0.40
Total cholesterol (mmol/l)	3×10^{-4} (1×10^{-4}, 4×10^{-4}) p=2×10^{-5}	2×10^{-4} (8×10^{-5}, 3×10^{-4}) p=1×10^{-3}	0.43
Cholesterol esters (mmol/l)	1×10^{-4} (8×10^{-5}, 2×10^{-4}) p=2×10^{-5}	1×10^{-4} (5×10^{-5}, 2×10^{-4}) p=1×10^{-3}	0.45
Free cholesterol (mmol/l)	1×10^{-4} (6×10^{-5}, 2×10^{-4}) p=5×10^{-5}	9×10^{-5} (3×10^{-5}, 1×10^{-4}) p=3×10^{-3}	0.43
Triglycerides (mmol/l)	1×10^{-3} (6×10^{-4}, 2×10^{-3}) p=4×10^{-5}	8×10^{-4} (3×10^{-4}, 1×10^{-3}) p=3×10^{-3}	0.36
Very large VLDL			
Particle concentration (mol/l)	4×10^{-11} (2×10^{-11}, 6×10^{-11}) p=2×10^{-5}	3×10^{-11} (1×10^{-11}, 5×10^{-11}) p=7×10^{-4}	0.46
Total lipids (mmol/l)	4×10^{-3} (2×10^{-3}, 0.01) p=2×10^{-5}	3×10^{-3} (1×10^{-3}, 5×10^{-3}) p=7×10^{-4}	0.47
Phospholipids (mmol/l)	6×10^{-4} (3×10^{-4}, 9×10^{-4}) p=2×10^{-5}	5×10^{-4} (2×10^{-4}, 8×10^{-4}) p=9×10^{-4}	0.50
Total cholesterol (mmol/l)	8×10^{-4} (4×10^{-4}, 1×10^{-3}) p=1×10^{-5}	6×10^{-4} (2×10^{-4}, 9×10^{-4}) p=1×10^{-3}	0.38
Cholesterol esters (mmol/l)	4×10^{-4} (2×10^{-4}, 6×10^{-4}) p=5×10^{-6}	3×10^{-4} (1×10^{-4}, 5×10^{-4}) p=9×10^{-4}	0.34
Free cholesterol (mmol/l)	4×10^{-4} (2×10^{-4}, 5×10^{-4}) p=3×10^{-5}	3×10^{-4} (1×10^{-4}, 4×10^{-4}) p=2×10^{-3}	0.43
Triglycerides (mmol/l)	2×10^{-3} (1×10^{-3}, 3×10^{-3}) p=2×10^{-5}	2×10^{-3} (8×10^{-4}, 3×10^{-3}) p=6×10^{-4}	0.50
Large VLDL			
Particle concentration (mol/l)	2×10^{-10} (1×10^{-10}, 3×10^{-10}) p=1×10^{-5}	2×10^{-10} (8×10^{-11}, 3×10^{-10}) p=2×10^{-4}	0.62
Total lipids (mmol/l)	0.01 (0.01, 0.02) p=1×10^{-5}	0.01 (5×10^{-3}, 0.02) p=2×10^{-4}	0.58
Phospholipids (mmol/l)	2×10^{-3} (1×10^{-3}, 3×10^{-3}) p=2×10^{-5}	2×10^{-3} (8×10^{-4}, 3×10^{-3}) p=2×10^{-4}	0.61
Total cholesterol (mmol/l)	3×10^{-3} (2×10^{-3}, 4×10^{-3}) p=8×10^{-6}	2×10^{-3} (1×10^{-3}, 4×10^{-3}) p=2×10^{-4}	0.54
Cholesterol esters (mmol/l)	1×10^{-3} (8×10^{-4}, 2×10^{-3}) p=6×10^{-6}	1×10^{-3} (5×10^{-4}, 2×10^{-3}) p=2×10^{-4}	0.49
Free cholesterol (mmol/l)	1×10^{-3} (8×10^{-4}, 2×10^{-3}) p=2×10^{-5}	1×10^{-3} (5×10^{-4}, 2×10^{-3}) p=3×10^{-4}	0.58
Triglycerides (mmol/l)	0.01 (4×10^{-3}, 0.01) p=2×10^{-5}	0.01 (3×10^{-3}, 0.01) p=2×10^{-4}	0.60
Medium VLDL			
Particle concentration (mol/l)	5×10^{-10} (3×10^{-10}, 7×10^{-10}) p=6×10^{-6}	4×10^{-10} (2×10^{-10}, 6×10^{-10}) p=2×10^{-4}	0.53
Total lipids (mmol/l)	0.02 (0.01, 0.02) p=5×10^{-6}	0.01 (0.01, 0.02) p=2×10^{-4}	0.51
Phospholipids (mmol/l)	3×10^{-3} (2×10^{-3}, 5×10^{-3}) p=9.7×10^{-6}	3×10^{-3} (1×10^{-3}, 4×10^{-3}) p=2×10^{-4}	0.56
Total cholesterol (mmol/l)	5×10^{-3} (3×10^{-3}, 0.01) p=9.8×10^{-6}	4×10^{-3} (2×10^{-3}, 0.01) p=3×10^{-4}	0.53
Cholesterol esters (mmol/l)	3×10^{-3} (1×10^{-3}, 4×10^{-3}) p=1×10^{-5}	2×10^{-3} (8×10^{-4}, 3×10^{-3}) p=6×10^{-4}	0.48
Free cholesterol (mmol/l)	2×10^{-3} (1×10^{-3}, 3×10^{-3}) p=2×10^{-5}	2×10^{-3} (8×10^{-4}, 3×10^{-3}) p=3×10^{-4}	0.61
Triglycerides (mmol/l)	0.01 (0.01, 0.01) p=4×10^{-6}	0.01 (4×10^{-3}, 0.01) p=2×10^{-4}	0.49
Small VLDL			
Particle concentration (mol/l)	5×10^{-10} (3×10^{-10}, 7×10^{-10}) p=1×10^{-5}	4×10^{-10} (2×10^{-10}, 7×10^{-10}) p=2×10^{-4}	0.64
Total lipids (mmol/l)	0.01 (0.01, 0.02) p=9×10^{-6}	0.01 (4×10^{-3}, 0.01) p=4×10^{-4}	0.53
Phospholipids (mmol/l)	2×10^{-3} (1×10^{-3}, 3×10^{-3}) p=9×10^{-6}	2×10^{-3} (9.7×10^{-4}, 3×10^{-3}) p=1×10^{-4}	0.74
Total cholesterol (mmol/l)	4×10^{-3} (2×10^{-3}, 0.01) p=4×10^{-5}	3×10^{-3} (7×10^{-4}, 5×10^{-3}) p=0.01	0.30
Cholesterol esters (mmol/l)	3×10^{-3} (1×10^{-3}, 4×10^{-3}) p=8×10^{-5}	1×10^{-3} (8×10^{-5}, 3×10^{-3}) p=0.04	0.18
Free cholesterol (mmol/l)	1×10^{-3} (7×10^{-4}, 2×10^{-3}) p=5×10^{-5}	1×10^{-3} (5×10^{-4}, 2×10^{-3}) p=6×10^{-4}	0.72
Triglycerides (mmol/l)	5×10^{-3} (2×10^{-3}, 0.01) p=3×10^{-5}	4×10^{-3} (2×10^{-3}, 0.01) p=2×10^{-4}	0.75
Very Small VLDL			
Particle concentration (mol/l)	2×10^{-10} (1×10^{-11}, 4×10^{-10}) p=0.04	2×10^{-10} (-1×10^{-11}, 4×10^{-10}) p=0.06	0.96
Total lipids (mmol/l)	4×10^{-3} (8×10^{-4}, 0.01) p=0.01	3×10^{-3} (-9×10^{-4}, 0.01) p=0.14	0.51
Phospholipids (mmol/l)	8×10^{-4} (-2×10^{-4}, 2×10^{-3}) p=0.12	6×10^{-4} (-5×10^{-4}, 2×10^{-3}) p=0.32	0.74
Total cholesterol (mmol/l)	2×10^{-3} (3×10^{-4}, 4×10^{-3}) p=0.02	6×10^{-4} (-1×10^{-3}, 3×10^{-3}) p=0.51	0.21
Cholesterol esters (mmol/l)	2×10^{-3} (6×10^{-4}, 3×10^{-3}) p=5×10^{-3}	5×10^{-4} (-8×10^{-4}, 2×10^{-3}) p=0.46	0.12
Free cholesterol (mmol/l)	3×10^{-4} (-3×10^{-4}, 8×10^{-4}) p=0.32	1×10^{-4} (-5×10^{-4}, 7×10^{-4}) p=0.68	0.68
Triglycerides (mmol/l)	1×10^{-3} (3×10^{-4}, 2×10^{-3}) p=0.01	1×10^{-3} (5×10^{-4}, 2×10^{-3}) p=1×10^{-3}	0.60
IDL			
Particle concentration (mol/l)	2×10^{-10} (-3×10^{-10}, 8×10^{-10}) p=0.41	4×10^{-11} (-5×10^{-10}, 6×10^{-10}) p=0.88	0.64
Total lipids (mmol/l)	4×10^{-3} (-3×10^{-3}, 0.01) p=0.26	1×10^{-3} (-0.01, 0.01) p=0.80	0.54
Phospholipids (mmol/l)	7×10^{-4} (-1×10^{-3}, 2×10^{-3}) p=0.45	-4×10^{-5} (-2×10^{-3}, 2×10^{-3}) p=0.96	0.56
Total cholesterol (mmol/l)	4×10^{-3} (-1×10^{-3}, 0.01) p=0.15	3×10^{-4} (-5×10^{-3}, 0.01) p=0.91	0.35

S5 Table. ALSPAC: offspring lipoproteins, lipids and metabolites absolute concentration differences per 1-SD higher parental BMI.

Avon Longitudinal Study of Parents and Children (ALSPAC)			
Metabolites	mother	father	P_{boot}
Cholesterol esters (mmol/l)	3×10^{-3} $(-2\times10^{-4}$, 0.01) p=0.06	7×10^{-4} $(-3\times10^{-3}, 5\times10^{-3})$ p=0.71	0.29
Free cholesterol (mmol/l)	2×10^{-4} $(-1\times10^{-3}, 2\times10^{-3})$ p=0.74	-4×10^{-4} $(-2\times10^{-3}, 1\times10^{-3})$ p=0.60	0.52
Triglycerides (mmol/l)	-1×10^{-4} $(-9\times10^{-4}, 7\times10^{-4})$ p=0.79	7×10^{-4} $(-1\times10^{-4}, 2\times10^{-3})$ p=0.10	0.16
Large LDL			
Particle concentration (mol/l)	6×10^{-10} $(-4\times10^{-10}, 2\times10^{-9})$ p=0.23	2×10^{-10} $(-8\times10^{-10}, 1\times10^{-9})$ p=0.68	0.59
Total lipids (mmol/l)	0.01 $(-3\times10^{-3}$, 0.01) p=0.21	2×10^{-3} (-0.01, 0.01) p=0.70	0.56
Phospholipids (mmol/l)	2×10^{-3} $(-3\times10^{-4}, 3\times10^{-3})$ p=0.10	6×10^{-4} $(-1\times10^{-3}, 3\times10^{-3})$ p=0.53	0.49
Total cholesterol (mmol/l)	4×10^{-3} $(-2\times10^{-3}$, 0.01) p=0.18	7×10^{-4} (-0.01, 0.01) p=0.84	0.42
Cholesterol esters (mmol/l)	4×10^{-3} $(-1\times10^{-3}$, 0.01) p=0.12	9.5×10^{-4} $(-4\times10^{-3}$, 0.01) p=0.73	0.41
Free cholesterol (mmol/l)	6×10^{-4} $(-1\times10^{-3}, 2\times10^{-3})$ p=0.49	-2×10^{-4} $(-2\times10^{-3}, 1\times10^{-3})$ p=0.80	0.48
Triglycerides (mmol/l)	-3×10^{-4} $(-1\times10^{-3}, 5\times10^{-4})$ p=0.46	5×10^{-4} $(-4\times10^{-4}, 1\times10^{-3})$ p=0.26	0.17
Medium LDL			
Particle concentration (mol/l)	7×10^{-10} $(-1\times10^{-10}, 2\times10^{-9})$ p=0.09	4×10^{-10} $(-5\times10^{-10}, 1\times10^{-9})$ p=0.44	0.55
Total lipids (mmol/l)	4×10^{-3} $(-1\times10^{-3}$, 0.01) p=0.12	2×10^{-3} $(-4\times10^{-3}$, 0.01) p=0.54	0.53
Phospholipids (mmol/l)	1×10^{-3} $(2\times10^{-4}, 2\times10^{-3})$ p=0.02	7×10^{-4} $(-5\times10^{-4}, 2\times10^{-3})$ p=0.25	0.47
Total cholesterol (mmol/l)	3×10^{-3} $(-8\times10^{-4}$, 0.01) p=0.12	9×10^{-4} $(-4\times10^{-3}$, 0.01) p=0.70	0.42
Cholesterol esters (mmol/l)	3×10^{-3} $(-6\times10^{-4}$, 0.01) p=0.11	8×10^{-4} $(-3\times10^{-3}, 4\times10^{-3})$ p=0.66	0.43
Free cholesterol (mmol/l)	5×10^{-4} $(-2\times10^{-4}, 1\times10^{-3})$ p=0.17	6×10^{-5} $(-8\times10^{-4}, 9\times10^{-4})$ p=0.89	0.39
Triglycerides (mmol/l)	-1×10^{-4} $(-6\times10^{-4}, 4\times10^{-4})$ p=0.60	3×10^{-4} $(-2\times10^{-4}, 8\times10^{-4})$ p=0.29	0.23
Small LDL			
Particle concentration (mol/l)	8×10^{-10} $(-1\times10^{-10}, 2\times10^{-9})$ p=0.09	3×10^{-10} $(-7\times10^{-10}, 1\times10^{-9})$ p=0.55	0.45
Total lipids (mmol/l)	3×10^{-3} $(-5\times10^{-4}$, 0.01) p=0.09	9.9×10^{-4} $(-3\times10^{-3}, 5\times10^{-3})$ p=0.60	0.43
Phospholipids (mmol/l)	8×10^{-4} $(3\times10^{-5}, 1\times10^{-3})$ p=0.04	3×10^{-4} $(-5\times10^{-4}, 1\times10^{-3})$ p=0.43	0.41
Total cholesterol (mmol/l)	2×10^{-3} $(-5\times10^{-4}, 4\times10^{-3})$ p=0.12	3×10^{-4} $(-2\times10^{-3}, 3\times10^{-3})$ p=0.83	0.35
Cholesterol esters (mmol/l)	2×10^{-3} $(-4\times10^{-4}, 4\times10^{-3})$ p=0.12	3×10^{-4} $(-2\times10^{-3}, 3\times10^{-3})$ p=0.78	0.38
Free cholesterol (mmol/l)	3×10^{-4} $(-1\times10^{-4}, 8\times10^{-4})$ p=0.15	-9.9×10^{-6} $(-5\times10^{-4}, 5\times10^{-4})$ p=0.97	0.28
Triglycerides (mmol/l)	2×10^{-4} $(-8\times10^{-5}, 5\times10^{-4})$ p=0.15	4×10^{-4} $(3\times10^{-5}, 7\times10^{-4})$ p=0.03	0.53
Very large HDL			
Particle concentration (mol/l)	-1×10^{-8} $(-1\times10^{-8}, -6\times10^{-9})$ $p=6\times10^{-7}$	-9×10^{-9} $(-1\times10^{-8}, -4\times10^{-9})$ $p=2\times10^{-4}$	0.48
Total lipids (mmol/l)	-0.01 (-0.02, -0.01) $p=1\times10^{-6}$	-0.01 (-0.02, -0.01) $p=7\times10^{-5}$	0.68
Phospholipids (mmol/l)	-0.01 (-0.01, -4×10^{-3}) $p=3\times10^{-7}$	-0.01 (-0.01, -2×10^{-3}) $p=4\times10^{-4}$	0.30
Total cholesterol (mmol/l)	-0.01 (-0.01, -3×10^{-3}) $p=2\times10^{-5}$	-0.01 (-0.01, -3×10^{-3}) $p=3\times10^{-5}$	0.79
Cholesterol esters (mmol/l)	-4×10^{-3} (-0.01, -2×10^{-3}) $p=9\times10^{-5}$	-4×10^{-3} (-0.01, -2×10^{-3}) $p=2\times10^{-5}$	0.62
Free cholesterol (mmol/l)	-2×10^{-3} $(-2\times10^{-3}, -9\times10^{-4})$ $p=2\times10^{-6}$	-1×10^{-3} $(-2\times10^{-3}, -7\times10^{-4})$ $p=1\times10^{-4}$	0.70
Triglycerides (mmol/l)	-2×10^{-4} $(-3\times10^{-4}, 1\times10^{-5})$ p=0.06	-8×10^{-5} $(-3\times10^{-4}, 1\times10^{-4})$ p=0.38	0.50
Large HDL			
Particle concentration (mol/l)	-2×10^{-8} $(-3\times10^{-8}, -1\times10^{-8})$ $p=3\times10^{-6}$	-1×10^{-8} $(-2\times10^{-8}, -4\times10^{-9})$ p=0.01	0.16
Total lipids (mmol/l)	-0.02 (-0.02, -0.01) $p=2\times10^{-6}$	-0.01 (-0.02, -3×10^{-3}) p=0.01	0.16
Phospholipids (mmol/l)	-0.01 (-0.01, -3×10^{-3}) $p=3\times10^{-5}$	-4×10^{-3} (-0.01, -6×10^{-4}) p=0.02	0.19
Total cholesterol (mmol/l)	-0.01 (-0.01, -0.01) $p=3\times10^{-7}$	-0.01 (-0.01, -2×10^{-3}) $p=2\times10^{-3}$	0.14
Cholesterol esters (mmol/l)	-0.01 (-0.01, -5×10^{-3}) $p=3\times10^{-7}$	-5×10^{-3} (-0.01, -2×10^{-3}) $p=2\times10^{-3}$	0.14
Free cholesterol (mmol/l)	-2×10^{-3} $(-3\times10^{-3}, -1\times10^{-3})$ $p=8\times10^{-7}$	-1×10^{-3} $(-2\times10^{-3}, -5\times10^{-4})$ $p=2\times10^{-3}$	0.18
Triglycerides (mmol/l)	-4×10^{-5} $(-2\times10^{-4}, 9.6\times10^{-5})$ p=0.58	2×10^{-5} $(-1\times10^{-4}, 2\times10^{-4})$ p=0.76	0.52
Medium HDL			
Particle concentration (mol/l)	1×10^{-9} $(-7\times10^{-9}, 9\times10^{-9})$ p=0.76	6×10^{-9} $(-2\times10^{-9}, 1\times10^{-8})$ p=0.16	0.35
Total lipids (mmol/l)	-1×10^{-4} $(-4\times10^{-3}, 4\times10^{-3})$ p=0.95	2×10^{-3} $(-2\times10^{-3}$, 0.01) p=0.30	0.36
Phospholipids (mmol/l)	-6×10^{-4} $(-3\times10^{-3}, 1\times10^{-3})$ p=0.58	9.8×10^{-4} $(-1\times10^{-3}, 3\times10^{-3})$ p=0.38	0.24
Total cholesterol (mmol/l)	-4×10^{-4} $(-2\times10^{-3}, 2\times10^{-3})$ p=0.71	4×10^{-4} $(-2\times10^{-3}, 2\times10^{-3})$ p=0.68	0.52
Cholesterol esters (mmol/l)	-4×10^{-4} $(-2\times10^{-3}, 1\times10^{-3})$ p=0.66	3×10^{-4} $(-1\times10^{-3}, 2\times10^{-3})$ p=0.74	0.54
Free cholesterol (mmol/l)	8×10^{-6} $(-3\times10^{-4}, 3\times10^{-4})$ p=0.96	1×10^{-4} $(-2\times10^{-4}, 5\times10^{-4})$ p=0.43	0.54
Triglycerides (mmol/l)	8×10^{-4} $(5\times10^{-4}, 1\times10^{-3})$ $p=4\times10^{-6}$	8×10^{-4} $(4\times10^{-4}, 1\times10^{-3})$ $p=5\times10^{-6}$	0.90
Small HDL			
Particle concentration (mol/l)	2×10^{-8} $(6\times10^{-9}, 3\times10^{-8})$ $p=3\times10^{-3}$	2×10^{-8} $(9\times10^{-9}, 3\times10^{-8})$ $p=7\times10^{-4}$	0.70
Total lipids (mmol/l)	3×10^{-3} $(-3\times10^{-4}$, 0.01) p=0.07	0.01 $(1\times10^{-3}$, 0.01) p=0.01	0.40

S5 Table. ALSPAC: offspring lipoproteins, lipids and metabolites absolute concentration differences per 1-SD higher parental BMI.

Avon Longitudinal Study of Parents and Children (ALSPAC)			
Metabolites	mother	father	P_{boot}
Phospholipids (mmol/l)	3×10^{-3} (5×10^{-4}, 5×10^{-3}) p=0.01	3×10^{-3} (9.6×10^{-4}, 0.01) p=5×10^{-3}	0.73
Total cholesterol (mmol/l)	-7×10^{-7} (-2×10^{-3}, 2×10^{-3}) p=1.00	1×10^{-3} (-6×10^{-4}, 3×10^{-3}) p=0.20	0.28
Cholesterol esters (mmol/l)	-1×10^{-5} (-1×10^{-3}, 1×10^{-3}) p=0.98	9×10^{-4} (-7×10^{-4}, 2×10^{-3}) p=0.26	0.33
Free cholesterol (mmol/l)	2×10^{-5} (-4×10^{-4}, 5×10^{-4}) p=0.95	3×10^{-4} (-2×10^{-4}, 8×10^{-4}) p=0.20	0.31
Triglycerides (mmol/l)	5×10^{-4} (2×10^{-4}, 9×10^{-4}) p=2×10^{-3}	7×10^{-4} (4×10^{-4}, 1×10^{-3}) p=7×10^{-5}	0.47
**Lipoprotein particle size**			
VLDL particle size (nm)	0.10 (0.05, 0.14) p=3×10^{-5}	0.07 (0.03, 0.12) p=9×10^{-4}	0.37
LDL particle size (nm)	-0.01 (-0.01, -3×10^{-3}) p=2×10^{-3}	-2×10^{-3} (-0.01, 3×10^{-3}) p=0.53	0.06
HDL particle size (nm)	-0.02 (-0.02, -0.01) p=9×10^{-8}	-0.01 (-0.02, -0.01) p=3×10^{-5}	0.43
**Cholesterol**			
Total cholesterol (mmol/l)	0.01 (-0.01, 0.04) p=0.30	2×10^{-3} (-0.02, 0.03) p=0.87	0.54
VLDL cholesterol (mmol/l)	0.01 (0.01, 0.02) p=1×10^{-5}	0.01 (3×10^{-3}, 0.02) p=4×10^{-3}	0.31
Remnant cholesterol (mmol/l)	0.02 (0.01, 0.03) p=7×10^{-4}	0.01 (-8×10^{-4}, 0.02) p=0.07	0.29
LDL cholesterol (mmol/l)	0.01 (-3×10^{-3}, 0.02) p=0.15	2×10^{-3} (-0.01, 0.02) p=0.79	0.41
HDL cholesterol (mmol/l)	-0.02 (-0.02, -0.01) p=2×10^{-4}	-0.01 (-0.02, -1×10^{-3}) p=0.02	0.34
HDL_2 cholesterol (mmol/l)	-0.01 (-0.02, -0.01) p=6×10^{-5}	-0.01 (-0.01, -2×10^{-3}) p=0.01	0.33
HDL_3 cholesterol (mmol/l)	-4×10^{-3} (-0.01, -1×10^{-3}) p=3×10^{-3}	-3×10^{-3} (-0.01, 5×10^{-4}) p=0.10	0.39
Esterified cholesterol (mmol/l)	0.01 (-0.01, 0.03) p=0.29	-1×10^{-3} (-0.02, 0.02) p=0.91	0.38
Free cholesterol (mmol/l)	3×10^{-3} (-4×10^{-3}, 0.01) p=0.36	3×10^{-3} (-5×10^{-3}, 0.01) p=0.40	0.99
**Glycerides and phospholipids**			
Triglycerides (mmol/l)	0.03 (0.01, 0.04) p=1×10^{-4}	0.03 (0.01, 0.04) p=3×10^{-4}	0.89
VLDL triglycerides (mmol/l)	0.03 (0.01, 0.04) p=1×10^{-5}	0.02 (0.01, 0.03) p=2×10^{-4}	0.61
LDL triglycerides (mmol/l)	-2×10^{-4} (-2×10^{-3}, 1×10^{-3}) p=0.79	1×10^{-3} (-6×10^{-4}, 3×10^{-3}) p=0.19	0.24
HDL triglycerides (mmol/l)	1×10^{-3} (3×10^{-4}, 2×10^{-3}) p=0.01	1×10^{-3} (6×10^{-4}, 2×10^{-3}) p=9×10^{-4}	0.62
Phosphoglycerides (mmol/l)	2×10^{-3} (-0.01, 0.01) p=0.76	4×10^{-3} (-0.01, 0.01) p=0.49	0.73
Phosphatidylcholine + other cholines (mmol/l)	8×10^{-4} (-0.01, 0.01) p=0.87	0.01 (-4×10^{-3}, 0.02) p=0.22	0.38
Cholines (mmol/l)	-7×10^{-4} (-0.01, 0.01) p=0.90	2×10^{-3} (-0.01, 0.01) p=0.78	0.75
**Apolipoproteins**			
Apolipoprotein A-I (g/l)	-5×10^{-3} (-0.01, -3×10^{-4}) p=0.04	-3×10^{-3} (-0.01, 2×10^{-3}) p=0.27	0.52
Apolipoprotein B (g/l)	0.01 (4×10^{-3}, 0.01) p=4×10^{-4}	0.01 (9×10^{-4}, 0.01) p=0.02	0.42
**Fatty acids**			
Total fatty acids (mmol/l)	0.07 (0.01, 0.13) p=0.02	0.07 (0.01, 0.14) p=0.03	0.97
Degree of unsaturation	-2×10^{-3} (-4×10^{-3}, 8×10^{-4}) p=0.19	-1×10^{-3} (-4×10^{-3}, 1×10^{-3}) p=0.38	0.76
Docosahexaenoic acid (mmol/l)	-1×10^{-3} (-2×10^{-3}, 9×10^{-5}) p=0.07	8×10^{-4} (-6×10^{-4}, 2×10^{-3}) p=0.25	0.03
Linoleic acid (mmol/l)	0.02 (1×10^{-4}, 0.03) p=0.05	-2×10^{-5} (-0.02, 0.02) p=1.00	0.14
n-3 fatty acids (mmol/l)	-3×10^{-4} (-3×10^{-3}, 2×10^{-3}) p=0.81	1×10^{-3} (-2×10^{-3}, 4×10^{-3}) p=0.40	0.41
n-6 fatty acids (mmol/l)	0.02 (-1×10^{-3}, 0.04) p=0.07	3×10^{-3} (-0.02, 0.02) p=0.73	0.27
PUFA (mmol/l)	0.02 (-4×10^{-3}, 0.04) p=0.12	5×10^{-3} (-0.02, 0.03) p=0.68	0.41
MUFA (mmol/l)	0.03 (0.01, 0.06) p=2×10^{-3}	0.04 (0.02, 0.06) p=8×10^{-4}	0.72
Saturated fatty acids (mmol/l)	0.02 (-0.01, 0.04) p=0.18	0.02 (-3×10^{-3}, 0.05) p=0.09	0.76
**Fatty acids ratios**			
Docosahexaenoic acid (%)	-0.02 (-0.03, -0.01) p=4×10^{-4}	4×10^{-4} (-0.01, 0.01) p=0.95	0.01
Linoleic acid (%)	-0.03 (-0.13, 0.07) p=0.58	-0.18 (-0.29, -0.08) p=5×10^{-4}	0.01
n-3 fatty acids (%)	-0.03 (-0.05, -4×10^{-3}) p=0.02	-0.01 (-0.03, 0.02) p=0.58	0.27
n-6 fatty acids (%)	-0.06 (-0.16, 0.04) p=0.23	-0.19 (-0.29, -0.09) p=3×10^{-4}	0.06
PUFA (%)	-0.09 (-0.19, 0.02) p=0.09	-0.20 (-0.31, -0.09) p=3×10^{-4}	0.12
MUFA (%)	0.18 (0.07, 0.30) p=2×10^{-3}	0.23 (0.11, 0.36) p=2×10^{-4}	0.52
Saturated fatty acids (%)	-0.09 (-0.17, -4×10^{-3}) p=0.04	-0.03 (-0.12, 0.05) p=0.45	0.34
**Glycolysis related metabolites**			
Glucose (mmol/l)	0.02 (4×10^{-4}, 0.04) p=0.04	0.01 (-0.01, 0.02) p=0.51	0.34
Lactate (mmol/l)	-6×10^{-4} (-0.02, 0.02) p=0.94	-0.01 (-0.03, 5×10^{-3}) p=0.14	0.25
Pyruvate (mmol/l)	2×10^{-3} (6×10^{-4}, 3×10^{-3}) p=4×10^{-3}	6×10^{-4} (-7×10^{-4}, 2×10^{-3}) p=0.33	0.16
Citrate (mmol/l)	-2×10^{-3} (-2×10^{-3}, -7×10^{-4}) p=5×10^{-4}	-1×10^{-3} (-2×10^{-3}, -4×10^{-4}) p=5×10^{-3}	0.69
**Amino acids**			

S5 Table. ALSPAC: offspring lipoproteins, lipids and metabolites absolute concentration differences per 1-SD higher parental BMI.

Avon Longitudinal Study of Parents and Children (ALSPAC)			
Metabolites	mother	father	P_{boot}
Alanine (mmol/l)	5×10^{-4} (-1×10^{-3}, 3×10^{-3}) p=0.62	-7×10^{-4} (-3×10^{-3}, 1×10^{-3}) p=0.53	0.37
Glutamine (mmol/l)	-6×10^{-5} (-3×10^{-3}, 3×10^{-3}) p=0.96	-3×10^{-3} (-0.01, -6×10^{-4}) p=0.01	0.06
Histidine (mmol/l)	5×10^{-4} (-2×10^{-5}, 9.6×10^{-4}) p=0.06	4×10^{-4} (-1×10^{-4}, 10×10^{-4}) p=0.11	0.96
Branched-chain amino acids			
Isoleucine (mmol/l)	1×10^{-3} (5×10^{-4}, 2×10^{-3}) p=5×10^{-5}	6×10^{-4} (9×10^{-5}, 1×10^{-3}) p=0.02	0.16
Leucine (mmol/l)	5×10^{-4} (2×10^{-4}, 9×10^{-4}) p=4×10^{-3}	4×10^{-4} (3×10^{-5}, 8×10^{-4}) p=0.03	0.64
Valine (mmol/l)	2×10^{-3} (1×10^{-3}, 3×10^{-3}) p=6×10^{-5}	1×10^{-3} (4×10^{-4}, 3×10^{-3}) p=0.01	0.34
Aromatic amino acids			
Phenylalanine (mmol/l)	3×10^{-4} (5×10^{-5}, 5×10^{-4}) p=0.02	1×10^{-4} (-9.9×10^{-5}, 3×10^{-4}) p=0.28	0.30
Tyrosine (mmol/l)	7×10^{-4} (3×10^{-4}, 1×10^{-3}) p=1×10^{-3}	3×10^{-4} (-2×10^{-4}, 7×10^{-4}) p=0.19	0.11
**Ketone bodies**			
Acetate (mmol/l)	-1×10^{-3} (-2×10^{-3}, -5×10^{-4}) p=2×10^{-3}	-9×10^{-4} (-2×10^{-3}, 1×10^{-4}) p=0.09	0.26
Acetoacetate (mmol/l)	-1×10^{-3} (-3×10^{-3}, -3×10^{-5}) p=0.04	4×10^{-5} (-2×10^{-3}, 2×10^{-3}) p=0.96	0.17
Beta-hydroxybutyrate (mmol/l)	-0.01 (-0.01, -2×10^{-3}) p=0.01	-2×10^{-3} (-0.01, 3×10^{-3}) p=0.39	0.26
**Fluid balance**			
Creatinine (mmol/l)	4×10^{-4} (-1×10^{-5}, 7×10^{-4}) p=0.06	5×10^{-4} (1×10^{-4}, 9×10^{-4}) p=0.01	0.58
Albumin (signal area)	-1×10^{-4} (-3×10^{-4}, 4×10^{-5}) p=0.15	-1×10^{-5} (-2×10^{-4}, 2×10^{-4}) p=0.89	0.37
**Inflammation**			
Glycoprotein acetyls (mmol/l)	0.01 (4×10^{-3}, 0.02) p=5×10^{-4}	0.01 (3×10^{-3}, 0.01) p=2×10^{-3}	0.70

Associations were adjusted for parental age, smoking, education, head of household social class, maternal parity, offspring's age at blood collection and sex.
S5 Table correspond to the SD-scaled metabolite concentration shown in S3 Fig.
