# Supplementary material for: Association of pre-pregnancy body mass index with offspring metabolic profile: Analyses of 3 European prospective birth cohorts
Source: PLoS Med. 2017 Aug 22;14(8):e1002376. doi: 10.1371/journal.pmed.1002376 (PMC5568725; doi:10.1371/journal.pmed.1002376)
Supplement: S6 Table — (PDF) [file pmed.1002376.s013.pdf]

S6 Table. NFBC66 and NFBC86: offspring lipoproteins, lipids and metabolites absolute concentration differences per 1-SD higher parental BMI.

	NFBC86			NFBC66
Metabolites	mother	father	P_{boot}	mother
**Lipoprotein subclasses**				
Extremely large VLDL				
Particle concentration (mol/l)	3×10^{-12} (-3×10^{-13}, 7×10^{-12}) p=0.07	2×10^{-12} (-9×10^{-13}, 6×10^{-12}) p=0.16	0.69	6×10^{-12} (2×10^{-12}, 1×10^{-11}) $p=3\times10^{-3}$
Total lipids (mmol/l)	7×10^{-4} (-7×10^{-5}, 1×10^{-3}) p=0.07	5×10^{-4} (-2×10^{-4}, 1×10^{-3}) p=0.16	0.69	1×10^{-3} (4×10^{-4}, 2×10^{-3}) $p=3\times10^{-3}$
Phospholipids (mmol/l)	8×10^{-5} (-1×10^{-5}, 2×10^{-4}) p=0.09	7×10^{-5} (-2×10^{-5}, 2×10^{-4}) p=0.11	0.86	2×10^{-4} (4×10^{-5}, 3×10^{-4}) p=0.01
Total cholesterol (mmol/l)	1×10^{-4} (-2×10^{-5}, 3×10^{-4}) p=0.08	9×10^{-5} (-5×10^{-5}, 2×10^{-4}) p=0.21	0.62	3×10^{-4} (1×10^{-4}, 5×10^{-4}) $p=2\times10^{-3}$
Cholesterol esters (mmol/l)	8×10^{-5} (-1×10^{-5}, 2×10^{-4}) p=0.09	4×10^{-5} (-4×10^{-5}, 1×10^{-4}) p=0.34	0.49	2×10^{-4} (7×10^{-5}, 3×10^{-4}) $p=1\times10^{-3}$
Free cholesterol (mmol/l)	6×10^{-5} (-7×10^{-6}, 1×10^{-4}) p=0.08	5×10^{-5} (-9×10^{-6}, 1×10^{-4}) p=0.10	0.86	1×10^{-4} (3×10^{-5}, 2×10^{-4}) $p=5\times10^{-3}$
Triglycerides (mmol/l)	5×10^{-4} (-4×10^{-5}, 1×10^{-3}) p=0.07	4×10^{-4} (-1×10^{-4}, 8×10^{-4}) p=0.16	0.69	9×10^{-4} (3×10^{-4}, 2×10^{-3}) $p=4\times10^{-3}$
Very large VLDL				
Particle concentration (mol/l)	2×10^{-11} (5×10^{-13}, 4×10^{-11}) p=0.04	2×10^{-11} (6×10^{-13}, 4×10^{-11}) p=0.04	0.92	3×10^{-11} (7×10^{-12}, 5×10^{-11}) p=0.01
Total lipids (mmol/l)	2×10^{-3} (3×10^{-5}, 4×10^{-3}) p=0.05	2×10^{-3} (3×10^{-5}, 4×10^{-3}) p=0.05	0.91	3×10^{-3} (8×10^{-4}, 0.01) p=0.01
Phospholipids (mmol/l)	4×10^{-4} (2×10^{-5}, 7×10^{-4}) p=0.04	3×10^{-4} (9×10^{-6}, 6×10^{-4}) p=0.04	0.89	5×10^{-4} (1×10^{-4}, 9×10^{-4}) p=0.01
Total cholesterol (mmol/l)	4×10^{-4} (-5×10^{-5}, 8×10^{-4}) p=0.08	3×10^{-4} (-8×10^{-5}, 7×10^{-4}) p=0.12	0.80	8×10^{-4} (3×10^{-4}, 1×10^{-3}) $p=2\times10^{-3}$
Cholesterol esters (mmol/l)	2×10^{-4} (-4×10^{-5}, 4×10^{-4}) p=0.10	1×10^{-4} (-7×10^{-5}, 4×10^{-4}) p=0.19	0.72	4×10^{-4} (2×10^{-4}, 7×10^{-4}) $p=2\times10^{-3}$
Free cholesterol (mmol/l)	2×10^{-4} (-1×10^{-5}, 4×10^{-4}) p=0.07	2×10^{-4} (-1×10^{-5}, 4×10^{-4}) p=0.07	0.90	3×10^{-4} (1×10^{-4}, 6×10^{-4}) $p=3\times10^{-3}$
Triglycerides (mmol/l)	1×10^{-3} (5×10^{-5}, 2×10^{-3}) p=0.04	1×10^{-3} (9×10^{-5}, 2×10^{-3}) p=0.03	0.96	2×10^{-3} (3×10^{-4}, 3×10^{-3}) p=0.01
Large VLDL				
Particle concentration (mol/l)	1×10^{-10} (1×10^{-11}, 2×10^{-10}) p=0.03	1×10^{-10} (2×10^{-11}, 2×10^{-10}) p=0.02	0.95	1×10^{-10} (3×10^{-11}, 3×10^{-10}) p=0.02
Total lipids (mmol/l)	0.01 (8×10^{-4}, 0.01) p=0.03	0.01 (8×10^{-4}, 0.01) p=0.03	0.94	0.01 (2×10^{-3}, 0.01) p=0.01
Phospholipids (mmol/l)	1×10^{-3} (2×10^{-4}, 2×10^{-3}) p=0.02	1×10^{-3} (2×10^{-4}, 2×10^{-3}) p=0.02	0.91	1×10^{-3} (3×10^{-4}, 3×10^{-3}) p=0.02
Total cholesterol (mmol/l)	2×10^{-3} (1×10^{-4}, 3×10^{-3}) p=0.04	1×10^{-3} (-1×10^{-4}, 3×10^{-3}) p=0.07	0.74	2×10^{-3} (7×10^{-4}, 4×10^{-3}) p=0.01
Cholesterol esters (mmol/l)	8×10^{-4} (4×10^{-5}, 2×10^{-3}) p=0.04	6×10^{-4} (-2×10^{-4}, 1×10^{-3}) p=0.15	0.57	1×10^{-3} (4×10^{-4}, 2×10^{-3}) $p=3\times10^{-3}$
Free cholesterol (mmol/l)	8×10^{-4} (5×10^{-5}, 2×10^{-3}) p=0.04	8×10^{-4} (6×10^{-5}, 1×10^{-3}) p=0.03	0.94	1×10^{-3} (2×10^{-4}, 2×10^{-3}) p=0.01
Triglycerides (mmol/l)	4×10^{-3} (5×10^{-4}, 0.01) p=0.03	4×10^{-3} (7×10^{-4}, 0.01) p=0.02	0.97	4×10^{-3} (6×10^{-4}, 0.01) p=0.02
Medium VLDL				
Particle concentration (mol/l)	3×10^{-10} (6×10^{-11}, 6×10^{-10}) p=0.01	3×10^{-10} (7×10^{-11}, 6×10^{-10}) p=0.01	0.96	3×10^{-10} (5×10^{-11}, 6×10^{-10}) p=0.02
Total lipids (mmol/l)	0.01 (2×10^{-3}, 0.02) p=0.01	0.01 (2×10^{-3}, 0.02) p=0.01	0.94	0.01 (2×10^{-3}, 0.02) p=0.02
Phospholipids (mmol/l)	2×10^{-3} (4×10^{-4}, 4×10^{-3}) p=0.01	2×10^{-3} (4×10^{-4}, 4×10^{-3}) p=0.01	0.92	2×10^{-3} (3×10^{-4}, 4×10^{-3}) p=0.02
Total cholesterol (mmol/l)	3×10^{-3} (5×10^{-4}, 0.01) p=0.02	2×10^{-3} (-1×10^{-4}, 5×10^{-3}) p=0.06	0.64	3×10^{-3} (7×10^{-4}, 0.01) p=0.01
Cholesterol esters (mmol/l)	2×10^{-3} (3×10^{-4}, 3×10^{-3}) p=0.02	10×10^{-4} (-4×10^{-4}, 2×10^{-3}) p=0.15	0.43	2×10^{-3} (5×10^{-4}, 4×10^{-3}) p=0.01
Free cholesterol (mmol/l)	1×10^{-3} (2×10^{-4}, 2×10^{-3}) p=0.02	1×10^{-3} (2×10^{-4}, 2×10^{-3}) p=0.02	0.95	1×10^{-3} (1×10^{-4}, 3×10^{-3}) p=0.03
Triglycerides (mmol/l)	0.01 (1×10^{-3}, 0.01) p=0.02	0.01 (2×10^{-3}, 0.01) p=0.01	0.90	0.01 (5×10^{-4}, 0.01) p=0.03
Small VLDL				
Particle concentration (mol/l)	4×10^{-10} (3×10^{-11}, 7×10^{-10}) p=0.03	4×10^{-10} (6×10^{-11}, 7×10^{-10}) p=0.02	0.90	4×10^{-10} (4×10^{-11}, 8×10^{-10}) p=0.03
Total lipids (mmol/l)	0.01 (4×10^{-4}, 0.01) p=0.04	0.01 (1×10^{-3}, 0.01) p=0.02	0.89	0.01 (7×10^{-4}, 0.02) p=0.03
Phospholipids (mmol/l)	1×10^{-3} (-1×10^{-4}, 3×10^{-3}) p=0.07	1×10^{-3} (1×10^{-4}, 3×10^{-3}) p=0.03	0.80	2×10^{-3} (5×10^{-5}, 3×10^{-3}) p=0.04
Total cholesterol (mmol/l)	2×10^{-3} (-4×10^{-4}, 5×10^{-3}) p=0.09	2×10^{-3} (-6×10^{-5}, 5×10^{-3}) p=0.06	0.90	3×10^{-3} (3×10^{-4}, 0.01) p=0.03
Cholesterol esters (mmol/l)	1×10^{-3} (-3×10^{-4}, 3×10^{-3}) p=0.10	2×10^{-3} (-2×10^{-4}, 3×10^{-3}) p=0.08	0.97	2×10^{-3} (3×10^{-4}, 4×10^{-3}) p=0.02
Free cholesterol (mmol/l)	7×10^{-4} (-2×10^{-4}, 2×10^{-3}) p=0.10	9×10^{-4} (3×10^{-5}, 2×10^{-3}) p=0.04	0.78	1×10^{-3} (-9×10^{-5}, 2×10^{-3}) p=0.07
Triglycerides (mmol/l)	3×10^{-3} (5×10^{-4}, 0.01) p=0.02	3×10^{-3} (7×10^{-4}, 0.01) p=0.01	0.95	3×10^{-3} (-3×10^{-5}, 0.01) p=0.05
Very Small VLDL				
Particle concentration (mol/l)	3×10^{-10} (-6×10^{-11}, 6×10^{-10}) p=0.11	2×10^{-10} (-1×10^{-10}, 5×10^{-10}) p=0.18	0.82	4×10^{-10} (-2×10^{-11}, 8×10^{-10}) p=0.06
Total lipids (mmol/l)	3×10^{-3} (-1×10^{-3}, 0.01) p=0.14	3×10^{-3} (-1×10^{-3}, 0.01) p=0.20	0.86	0.01 (-1×10^{-4}, 0.01) p=0.06
Phospholipids (mmol/l)	9.7×10^{-4} (-3×10^{-4}, 2×10^{-3}) p=0.15	6×10^{-4} (-6×10^{-4}, 2×10^{-3}) p=0.33	0.71	1×10^{-3} (-4×10^{-4}, 3×10^{-3}) p=0.14
Total cholesterol (mmol/l)	1×10^{-3} (-1×10^{-3}, 4×10^{-3}) p=0.27	1×10^{-3} (-9.8×10^{-4}, 4×10^{-3}) p=0.26	0.99	3×10^{-3} (4×10^{-4}, 0.01) p=0.02
Cholesterol esters (mmol/l)	1×10^{-3} (-7×10^{-4}, 3×10^{-3}) p=0.24	9×10^{-4} (-8×10^{-4}, 3×10^{-3}) p=0.30	0.88	2×10^{-3} (5×10^{-4}, 4×10^{-3}) p=0.01
Free cholesterol (mmol/l)	3×10^{-4} (-4×10^{-4}, 10×10^{-4}) p=0.42	4×10^{-4} (-3×10^{-4}, 1×10^{-3}) p=0.22	0.76	8×10^{-4} (-2×10^{-4}, 2×10^{-3}) p=0.11
Triglycerides (mmol/l)	9×10^{-4} (-2×10^{-5}, 2×10^{-3}) p=0.06	7×10^{-4} (-2×10^{-4}, 2×10^{-3}) p=0.13	0.80	9×10^{-4} (-5×10^{-4}, 2×10^{-3}) p=0.22
IDL				
Particle concentration (mol/l)	5×10^{-10} (-5×10^{-10}, 1×10^{-9}) p=0.34	2×10^{-10} (-7×10^{-10}, 1×10^{-9}) p=0.61	0.72	8×10^{-10} (-3×10^{-10}, 2×10^{-9}) p=0.17
Total lipids (mmol/l)	4×10^{-3} (-0.01, 0.01) p=0.36	2×10^{-3} (-0.01, 0.01) p=0.63	0.74	0.01 (-4×10^{-3}, 0.02) p=0.17
Phospholipids (mmol/l)	1×10^{-3} (-1×10^{-3}, 4×10^{-3}) p=0.37	5×10^{-4} (-2×10^{-3}, 3×10^{-3}) p=0.66	0.72	2×10^{-3} (-1×10^{-3}, 5×10^{-3}) p=0.25
Total cholesterol (mmol/l)	3×10^{-3} (-3×10^{-3}, 0.01) p=0.35	2×10^{-3} (-4×10^{-3}, 0.01) p=0.54	0.79	0.01 (-2×10^{-3}, 0.01) p=0.14

S6 Table. NFBC66 and NFBC86: offspring lipoproteins, lipids and metabolites absolute concentration differences per 1-SD higher parental BMI.

	NFBC86			NFBC66
Metabolites	mother	father	P_{boot}	mother
Cholesterol esters (mmol/l)	$3\times10^{-3}\ (-2\times10^{-3}, 0.01)\ p=0.28$	$2\times10^{-3}\ (-3\times10^{-3}, 0.01)\ p=0.44$	0.79	$5\times10^{-3}\ (-8\times10^{-4}, 0.01)\ p=0.10$
Free cholesterol (mmol/l)	$5\times10^{-4}\ (-1\times10^{-3}, 2\times10^{-3})\ p=0.61$	$2\times10^{-4}\ (-2\times10^{-3}, 2\times10^{-3})\ p=0.86$	0.80	$1\times10^{-3}\ (-1\times10^{-3}, 4\times10^{-3})\ p=0.31$
Triglycerides (mmol/l)	$5\times10^{-4}\ (-4\times10^{-4}, 1\times10^{-3})\ p=0.29$	$8\times10^{-5}\ (-9\times10^{-4}, 1\times10^{-3})\ p=0.88$	0.52	$7\times10^{-4}\ (-9\times10^{-4}, 2\times10^{-3})\ p=0.39$
Large LDL				
Particle concentration (mol/l)	$7\times10^{-10}\ (-9\times10^{-10}, 2\times10^{-9})\ p=0.39$	$6\times10^{-10}\ (-9.7\times10^{-10}, 2\times10^{-9})\ p=0.46$	0.93	$1\times10^{-9}\ (-7\times10^{-10}, 3\times10^{-9})\ p=0.19$
Total lipids (mmol/l)	$5\times10^{-3}\ (-0.01, 0.02)\ p=0.40$	$4\times10^{-3}\ (-0.01, 0.02)\ p=0.46$	0.93	$0.01\ (-5\times10^{-3}, 0.02)\ p=0.20$
Phospholipids (mmol/l)	$1\times10^{-3}\ (-1\times10^{-3}, 4\times10^{-3})\ p=0.34$	$1\times10^{-3}\ (-1\times10^{-3}, 3\times10^{-3})\ p=0.40$	0.92	$2\times10^{-3}\ (-1\times10^{-3}, 5\times10^{-3})\ p=0.23$
Total cholesterol (mmol/l)	$4\times10^{-3}\ (-5\times10^{-3}, 0.01)\ p=0.40$	$3\times10^{-3}\ (-5\times10^{-3}, 0.01)\ p=0.43$	0.97	$0.01\ (-3\times10^{-3}, 0.02)\ p=0.19$
Cholesterol esters (mmol/l)	$3\times10^{-3}\ (-3\times10^{-3}, 0.01)\ p=0.35$	$3\times10^{-3}\ (-3\times10^{-3}, 0.01)\ p=0.36$	0.98	$0.01\ (-2\times10^{-3}, 0.01)\ p=0.15$
Free cholesterol (mmol/l)	$6\times10^{-4}\ (-1\times10^{-3}, 3\times10^{-3})\ p=0.60$	$4\times10^{-4}\ (-2\times10^{-3}, 2\times10^{-3})\ p=0.68$	0.93	$1\times10^{-3}\ (-1\times10^{-3}, 4\times10^{-3})\ p=0.33$
Triglycerides (mmol/l)	$1\times10^{-4}\ (-8\times10^{-4}, 1\times10^{-3})\ p=0.78$	$-1\times10^{-4}\ (-1\times10^{-3}, 8\times10^{-4})\ p=0.80$	0.69	$6\times10^{-4}\ (-9\times10^{-4}, 2\times10^{-3})\ p=0.43$
Medium LDL				
Particle concentration (mol/l)	$6\times10^{-10}\ (-8\times10^{-10}, 2\times10^{-9})\ p=0.40$	$6\times10^{-10}\ (-8\times10^{-10}, 2\times10^{-9})\ p=0.40$	0.99	$1\times10^{-9}\ (-6\times10^{-10}, 3\times10^{-9})\ p=0.19$
Total lipids (mmol/l)	$3\times10^{-3}\ (-4\times10^{-3}, 0.01)\ p=0.40$	$3\times10^{-3}\ (-4\times10^{-3}, 0.01)\ p=0.39$	0.98	$0.01\ (-3\times10^{-3}, 0.01)\ p=0.19$
Phospholipids (mmol/l)	$9\times10^{-4}\ (-5\times10^{-4}, 2\times10^{-3})\ p=0.21$	$7\times10^{-4}\ (-7\times10^{-4}, 2\times10^{-3})\ p=0.34$	0.83	$1\times10^{-3}\ (-6\times10^{-4}, 3\times10^{-3})\ p=0.19$
Total cholesterol (mmol/l)	$2\times10^{-3}\ (-3\times10^{-3}, 0.01)\ p=0.44$	$2\times10^{-3}\ (-3\times10^{-3}, 0.01)\ p=0.37$	0.92	$4\times10^{-3}\ (-2\times10^{-3}, 0.01)\ p=0.19$
Cholesterol esters (mmol/l)	$2\times10^{-3}\ (-2\times10^{-3}, 0.01)\ p=0.43$	$2\times10^{-3}\ (-2\times10^{-3}, 0.01)\ p=0.35$	0.90	$3\times10^{-3}\ (-2\times10^{-3}, 0.01)\ p=0.18$
Free cholesterol (mmol/l)	$3\times10^{-4}\ (-7\times10^{-4}, 1\times10^{-3})\ p=0.51$	$4\times10^{-4}\ (-7\times10^{-4}, 1\times10^{-3})\ p=0.48$	0.97	$7\times10^{-4}\ (-5\times10^{-4}, 2\times10^{-3})\ p=0.27$
Triglycerides (mmol/l)	$-3\times10^{-5}\ (-5\times10^{-4}, 5\times10^{-4})\ p=0.90$	$-9.9\times10^{-5}\ (-6\times10^{-4}, 4\times10^{-4})\ p=0.70$	0.84	$3\times10^{-4}\ (-5\times10^{-4}, 1\times10^{-3})\ p=0.48$
Small LDL				
Particle concentration (mol/l)	$4\times10^{-10}\ (-1\times10^{-9}, 2\times10^{-9})\ p=0.58$	$7\times10^{-10}\ (-7\times10^{-10}, 2\times10^{-9})\ p=0.33$	0.76	$1\times10^{-9}\ (-6\times10^{-10}, 3\times10^{-9})\ p=0.19$
Total lipids (mmol/l)	$1\times10^{-3}\ (-3\times10^{-3}, 0.01)\ p=0.61$	$2\times10^{-3}\ (-2\times10^{-3}, 0.01)\ p=0.33$	0.74	$3\times10^{-3}\ (-2\times10^{-3}, 0.01)\ p=0.20$
Phospholipids (mmol/l)	$3\times10^{-4}\ (-7\times10^{-4}, 1\times10^{-3})\ p=0.54$	$6\times10^{-4}\ (-4\times10^{-4}, 2\times10^{-3})\ p=0.23$	0.69	$7\times10^{-4}\ (-3\times10^{-4}, 2\times10^{-3})\ p=0.19$
Total cholesterol (mmol/l)	$7\times10^{-4}\ (-2\times10^{-3}, 4\times10^{-3})\ p=0.64$	$1\times10^{-3}\ (-2\times10^{-3}, 5\times10^{-3})\ p=0.36$	0.75	$2\times10^{-3}\ (-1\times10^{-3}, 0.01)\ p=0.23$
Cholesterol esters (mmol/l)	$7\times10^{-4}\ (-2\times10^{-3}, 3\times10^{-3})\ p=0.58$	$1\times10^{-3}\ (-1\times10^{-3}, 4\times10^{-3})\ p=0.36$	0.80	$2\times10^{-3}\ (-1\times10^{-3}, 5\times10^{-3})\ p=0.24$
Free cholesterol (mmol/l)	$4\times10^{-5}\ (-6\times10^{-4}, 7\times10^{-4})\ p=0.91$	$3\times10^{-4}\ (-3\times10^{-4}, 9\times10^{-4})\ p=0.38$	0.60	$5\times10^{-4}\ (-3\times10^{-4}, 1\times10^{-3})\ p=0.21$
Triglycerides (mmol/l)	$5\times10^{-5}\ (-2\times10^{-4}, 4\times10^{-4})\ p=0.72$	$7\times10^{-5}\ (-2\times10^{-4}, 4\times10^{-4})\ p=0.68$	0.96	$3\times10^{-4}\ (-2\times10^{-4}, 8\times10^{-4})\ p=0.23$
Very large HDL				
Particle concentration (mol/l)	$-1\times10^{-8}\ (-2\times10^{-8}, -5\times10^{-9})\ p=5\times10^{-4}$	$-1\times10^{-8}\ (-2\times10^{-8}, -4\times10^{-9})\ p=3\times10^{-3}$	0.71	$-6\times10^{-9}\ (-1\times10^{-8}, 5\times10^{-10})\ p=0.07$
Total lipids (mmol/l)	$-0.01\ (-0.02, -0.01)\ p=6\times10^{-4}$	$-0.01\ (-0.02, -4\times10^{-3})\ p=4\times10^{-3}$	0.70	$-0.01\ (-0.01, 6\times10^{-4})\ p=0.07$
Phospholipids (mmol/l)	$-0.01\ (-0.01, -3\times10^{-3})\ p=6\times10^{-4}$	$-0.01\ (-0.01, -3\times10^{-3})\ p=9.8\times10^{-4}$	0.96	$-5\times10^{-3}\ (-0.01, -1\times10^{-3})\ p=0.01$
Total cholesterol (mmol/l)	$-0.01\ (-0.01, -3\times10^{-3})\ p=1\times10^{-3}$	$-4\times10^{-3}\ (-0.01, -8\times10^{-4})\ p=0.02$	0.49	$-2\times10^{-3}\ (-5\times10^{-3}, 2\times10^{-3})\ p=0.35$
Cholesterol esters (mmol/l)	$-5\times10^{-3}\ (-0.01, -2\times10^{-3})\ p=1\times10^{-3}$	$-3\times10^{-3}\ (-0.01, -2\times10^{-4})\ p=0.03$	0.42	$-8\times10^{-4}\ (-3\times10^{-3}, 2\times10^{-3})\ p=0.53$
Free cholesterol (mmol/l)	$-2\times10^{-3}\ (-3\times10^{-3}, -7\times10^{-4})\ p=9\times10^{-4}$	$-1\times10^{-3}\ (-2\times10^{-3}, -5\times10^{-4})\ p=3\times10^{-3}$	0.76	$-8\times10^{-4}\ (-2\times10^{-3}, 2\times10^{-4})\ p=0.11$
Triglycerides (mmol/l)	$-2\times10^{-4}\ (-4\times10^{-4}, 9\times10^{-5})\ p=0.23$	$-1\times10^{-4}\ (-4\times10^{-4}, 9.9\times10^{-5})\ p=0.25$	0.94	$4\times10^{-5}\ (-3\times10^{-4}, 4\times10^{-4})\ p=0.83$
Large HDL				
Particle concentration (mol/l)	$-3\times10^{-8}\ (-5\times10^{-8}, -1\times10^{-8})\ p=1\times10^{-3}$	$-2\times10^{-8}\ (-4\times10^{-8}, -8\times10^{-9})\ p=4\times10^{-3}$	0.72	$-3\times10^{-8}\ (-5\times10^{-8}, -9.6\times10^{-9})\ p=3\times10^{-3}$
Total lipids (mmol/l)	$-0.02\ (-0.03, -0.01)\ p=1\times10^{-3}$	$-0.02\ (-0.03, -0.01)\ p=3\times10^{-3}$	0.72	$-0.02\ (-0.03, -0.01)\ p=3\times10^{-3}$
Phospholipids (mmol/l)	$-0.01\ (-0.01, -3\times10^{-3})\ p=2\times10^{-3}$	$-0.01\ (-0.01, -1\times10^{-3})\ p=0.01$	0.59	$-0.01\ (-0.01, -2\times10^{-3})\ p=4\times10^{-3}$
Total cholesterol (mmol/l)	$-0.01\ (-0.02, -4\times10^{-3})\ p=6\times10^{-4}$	$-0.01\ (-0.01, -4\times10^{-3})\ p=1\times10^{-3}$	0.80	$-0.01\ (-0.02, -4\times10^{-3})\ p=2\times10^{-3}$
Cholesterol esters (mmol/l)	$-0.01\ (-0.01, -3\times10^{-3})\ p=7\times10^{-4}$	$-0.01\ (-0.01, -3\times10^{-3})\ p=1\times10^{-3}$	0.80	$-0.01\ (-0.01, -3\times10^{-3})\ p=2\times10^{-3}$
Free cholesterol (mmol/l)	$-3\times10^{-3}\ (-4\times10^{-3}, -1\times10^{-3})\ p=3\times10^{-4}$	$-2\times10^{-3}\ (-4\times10^{-3}, -9.7\times10^{-4})\ p=7\times10^{-4}$	0.81	$-2\times10^{-3}\ (-4\times10^{-3}, -8\times10^{-4})\ p=2\times10^{-3}$
Triglycerides (mmol/l)	$-4\times10^{-4}\ (-9.6\times10^{-4}, 2\times10^{-4})\ p=0.22$	$-5\times10^{-4}\ (-1\times10^{-3}, 5\times10^{-5})\ p=0.07$	0.74	$-6\times10^{-4}\ (-1\times10^{-3}, 2\times10^{-4})\ p=0.16$
Medium HDL				
Particle concentration (mol/l)	$-2\times10^{-9}\ (-2\times10^{-8}, 2\times10^{-8})\ p=0.86$	$5\times10^{-9}\ (-1\times10^{-8}, 2\times10^{-8})\ p=0.52$	0.54	$-1\times10^{-8}\ (-3\times10^{-8}, 6\times10^{-9})\ p=0.16$
Total lipids (mmol/l)	$-9\times10^{-4}\ (-0.01, 0.01)\ p=0.80$	$2\times10^{-3}\ (-5\times10^{-3}, 0.01)\ p=0.54$	0.53	$-0.01\ (-0.01, 2\times10^{-3})\ p=0.15$
Phospholipids (mmol/l)	$-7\times10^{-4}\ (-4\times10^{-3}, 2\times10^{-3})\ p=0.67$	$8\times10^{-4}\ (-2\times10^{-3}, 4\times10^{-3})\ p=0.57$	0.46	$-3\times10^{-3}\ (-0.01, 9\times10^{-4})\ p=0.15$
Total cholesterol (mmol/l)	$-8\times10^{-4}\ (-5\times10^{-3}, 3\times10^{-3})\ p=0.70$	$1\times10^{-3}\ (-3\times10^{-3}, 5\times10^{-3})\ p=0.59$	0.50	$-4\times10^{-3}\ (-0.01, 7\times10^{-4})\ p=0.10$
Cholesterol esters (mmol/l)	$-5\times10^{-4}\ (-4\times10^{-3}, 3\times10^{-3})\ p=0.76$	$9\times10^{-4}\ (-2\times10^{-3}, 4\times10^{-3})\ p=0.56$	0.52	$-3\times10^{-3}\ (-0.01, 5\times10^{-4})\ p=0.09$
Free cholesterol (mmol/l)	$-3\times10^{-4}\ (-1\times10^{-3}, 6\times10^{-4})\ p=0.50$	$2\times10^{-4}\ (-6\times10^{-4}, 9.5\times10^{-4})\ p=0.71$	0.44	$-8\times10^{-4}\ (-2\times10^{-3}, 2\times10^{-4})\ p=0.13$
Triglycerides (mmol/l)	$6\times10^{-4}\ (5\times10^{-5}, 1\times10^{-3})\ p=0.03$	$3\times10^{-4}\ (-2\times10^{-4}, 8\times10^{-4})\ p=0.25$	0.42	$4\times10^{-4}\ (-2\times10^{-4}, 1\times10^{-3})\ p=0.14$
Small HDL				
Particle concentration (mol/l)	$1\times10^{-8}\ (-1\times10^{-8}, 4\times10^{-8})\ p=0.37$	$3\times10^{-8}\ (6\times10^{-9}, 5\times10^{-8})\ p=0.02$	0.26	$-2\times10^{-9}\ (-3\times10^{-8}, 2\times10^{-8})\ p=0.87$
Total lipids (mmol/l)	$2\times10^{-3}\ (-3\times10^{-3}, 0.01)\ p=0.42$	$0.01\ (1\times10^{-3}, 0.01)\ p=0.02$	0.25	$-8\times10^{-4}\ (-0.01, 0.01)\ p=0.79$

S6 Table. NFBC66 and NFBC86: offspring lipoproteins, lipids and metabolites absolute concentration differences per 1-SD higher parental BMI.

	**NFBC86**			**NFBC66**
Metabolites	mother	father	P_{boot}	mother
Phospholipids (mmol/l)	9×10^{-4} (-2×10^{-3}, 4×10^{-3}) p=0.56	3×10^{-3} (4×10^{-4}, 0.01) p=0.02	0.22	-6×10^{-4} (-4×10^{-3}, 3×10^{-3}) p=0.71
Total cholesterol (mmol/l)	9×10^{-4} (-2×10^{-3}, 4×10^{-3}) p=0.52	3×10^{-3} (-8×10^{-5}, 0.01) p=0.06	0.35	-8×10^{-4} (-4×10^{-3}, 2×10^{-3}) p=0.63
Cholesterol esters (mmol/l)	1×10^{-3} (-1×10^{-3}, 3×10^{-3}) p=0.41	2×10^{-3} (-2×10^{-4}, 5×10^{-3}) p=0.07	0.46	-4×10^{-4} (-3×10^{-3}, 2×10^{-3}) p=0.73
Free cholesterol (mmol/l)	-8×10^{-5} (-7×10^{-4}, 6×10^{-4}) p=0.81	5×10^{-4} (-9×10^{-5}, 1×10^{-3}) p=0.10	0.16	-3×10^{-4} (-1×10^{-3}, 4×10^{-4}) p=0.40
Triglycerides (mmol/l)	4×10^{-4} (-1×10^{-5}, 9×10^{-4}) p=0.06	4×10^{-4} (-5×10^{-5}, 9×10^{-4}) p=0.08	0.91	6×10^{-4} (-3×10^{-5}, 1×10^{-3}) p=0.06
**Lipoprotein particle size**				
VLDL particle size (nm)	0.04 (-0.01, 0.08) p=0.10	0.05 (4×10^{-3}, 0.09) p=0.03	0.72	0.04 (5×10^{-3}, 0.07) p=0.03
LDL particle size (nm)	6×10^{-4} (-0.01, 0.01) p=0.85	-0.01 (-0.01, 5×10^{-4}) p=0.07	0.16	-3×10^{-5} (-4×10^{-3}, 4×10^{-3}) p=0.99
HDL particle size (nm)	-0.01 (-0.02, -0.01) p=3×10^{-4}	-0.02 (-0.03, -0.01) p=4×10^{-5}	0.70	-0.01 (-0.02, -4×10^{-3}) p=2×10^{-3}
**Cholesterol**				
Total cholesterol (mmol/l)	6×10^{-4} (-0.03, 0.03) p=0.97	0.01 (-0.02, 0.04) p=0.68	0.80	0.02 (-0.02, 0.05) p=0.43
VLDL cholesterol (mmol/l)	0.01 (7×10^{-5}, 0.02) p=0.05	0.01 (-3×10^{-4}, 0.02) p=0.06	0.86	0.01 (4×10^{-3}, 0.02) p=4×10^{-3}
Remnant cholesterol (mmol/l)	0.01 (-2×10^{-3}, 0.03) p=0.10	0.01 (-4×10^{-3}, 0.02) p=0.16	0.82	0.02 (3×10^{-3}, 0.03) p=0.02
LDL cholesterol (mmol/l)	0.01 (-0.01, 0.02) p=0.50	0.01 (-0.01, 0.02) p=0.40	0.89	0.01 (-0.01, 0.03) p=0.20
HDL cholesterol (mmol/l)	-0.02 (-0.03, -0.01) p=3×10^{-3}	-0.01 (-0.02, 3×10^{-4}) p=0.06	0.42	-0.02 (-0.03, -4×10^{-3}) p=0.01
HDL_2 cholesterol (mmol/l)	-0.02 (-0.03, -0.01) p=3×10^{-3}	-0.01 (-0.02, -6×10^{-4}) p=0.04	0.48	-0.02 (-0.03, -4×10^{-3}) p=0.01
HDL_3 cholesterol (mmol/l)	-1×10^{-3} (-3×10^{-3}, -7×10^{-5}) p=0.04	-4×10^{-5} (-1×10^{-3}, 1×10^{-3}) p=0.95	0.17	-4×10^{-4} (-2×10^{-3}, 1×10^{-3}) p=0.67
Esterified cholesterol (mmol/l)	2×10^{-4} (-0.02, 0.02) p=0.99	0.01 (-0.02, 0.03) p=0.53	0.66	0.01 (-0.01, 0.04) p=0.32
Free cholesterol (mmol/l)	5×10^{-4} (-0.01, 0.01) p=0.91	-7×10^{-4} (-0.01, 0.01) p=0.88	0.84	5×10^{-3} (-0.01, 0.02) p=0.40
**Glycerides and phospholipids**				
Triglycerides (mmol/l)	0.02 (2×10^{-3}, 0.03) p=0.03	0.02 (1×10^{-3}, 0.03) p=0.03	0.93	0.02 (2×10^{-4}, 0.04) p=0.05
VLDL triglycerides (mmol/l)	0.02 (2×10^{-3}, 0.03) p=0.02	0.02 (4×10^{-3}, 0.03) p=0.01	0.95	0.02 (2×10^{-3}, 0.03) p=0.03
LDL triglycerides (mmol/l)	1×10^{-4} (-2×10^{-3}, 2×10^{-3}) p=0.89	-2×10^{-4} (-2×10^{-3}, 2×10^{-3}) p=0.83	0.79	1×10^{-3} (-2×10^{-3}, 4×10^{-3}) p=0.41
HDL triglycerides (mmol/l)	6×10^{-4} (-8×10^{-4}, 2×10^{-3}) p=0.41	-3×10^{-5} (-1×10^{-3}, 1×10^{-3}) p=0.96	0.51	7×10^{-4} (-1×10^{-3}, 3×10^{-3}) p=0.51
Phosphoglycerides (mmol/l)	-4×10^{-3} (-0.02, 0.01) p=0.53	2×10^{-3} (-0.01, 0.01) p=0.77	0.51	3×10^{-3} (-0.01, 0.02) p=0.74
Phosphatidylcholine + other cholines (mmol/l)	-5×10^{-3} (-0.02, 0.01) p=0.41	8×10^{-4} (-0.01, 0.01) p=0.89	0.49	1×10^{-3} (-0.01, 0.02) p=0.88
Cholines (mmol/l)	-5×10^{-3} (-0.02, 0.01) p=0.48	1×10^{-3} (-0.01, 0.01) p=0.87	0.53	2×10^{-3} (-0.01, 0.02) p=0.81
**Apolipoproteins**				
Apolipoprotein A-I (g/l)	-0.01 (-0.01, 3×10^{-4}) p=0.06	-3×10^{-3} (-0.01, 4×10^{-3}) p=0.41	0.44	-0.01 (-0.02, 1×10^{-3}) p=0.08
Apolipoprotein B (g/l)	0.01 (-8×10^{-4}, 0.01) p=0.09	5×10^{-3} (-1×10^{-3}, 0.01) p=0.12	0.88	0.01 (5×10^{-4}, 0.02) p=0.04
**Fatty acids**				
Total fatty acids (mmol/l)	0.03 (-0.05, 0.10) p=0.48	0.04 (-0.03, 0.11) p=0.27	0.78	0.06 (-0.03, 0.15) p=0.21
Degree of unsaturation	-4×10^{-4} (-2×10^{-3}, 1×10^{-3}) p=0.64	8×10^{-4} (-9×10^{-4}, 3×10^{-3}) p=0.34	0.28	-3×10^{-4} (-2×10^{-3}, 1×10^{-3}) p=0.75
Docosahexaenoic acid (mmol/l)	2×10^{-4} (-1×10^{-3}, 2×10^{-3}) p=0.82	2×10^{-3} (-2×10^{-5}, 3×10^{-3}) p=0.05	0.21	1×10^{-3} (-8×10^{-4}, 3×10^{-3}) p=0.24
Linoleic acid (mmol/l)	-0.01 (-0.02, 0.01) p=0.45	0.01 (-0.01, 0.02) p=0.47	0.28	-1×10^{-3} (-0.02, 0.02) p=0.92
n-3 fatty acids (mmol/l)	9×10^{-4} (-3×10^{-3}, 0.01) p=0.67	5×10^{-3} (5×10^{-4}, 0.01) p=0.03	0.20	3×10^{-3} (-2×10^{-3}, 0.01) p=0.25
n-6 fatty acids (mmol/l)	-0.01 (-0.03, 0.02) p=0.62	0.01 (-0.01, 0.03) p=0.43	0.35	4×10^{-3} (-0.02, 0.03) p=0.75
PUFA (mmol/l)	-5×10^{-3} (-0.03, 0.02) p=0.72	0.01 (-0.01, 0.04) p=0.29	0.30	0.01 (-0.02, 0.04) p=0.64
MUFA (mmol/l)	0.02 (-0.01, 0.04) p=0.13	0.02 (-0.01, 0.04) p=0.15	0.96	0.02 (-0.01, 0.06) p=0.12
Saturated fatty acids (mmol/l)	0.01 (-0.02, 0.04) p=0.42	0.01 (-0.02, 0.04) p=0.54	0.88	0.03 (-0.01, 0.06) p=0.15
**Fatty acids ratios**				
Docosahexaenoic acid (%)	-9×10^{-4} (-0.01, 0.01) p=0.87	0.01 (-2×10^{-3}, 0.02) p=0.10	0.17	4×10^{-3} (-0.01, 0.01) p=0.48
Linoleic acid (%)	-0.11 (-0.19, -0.04) p=4×10^{-3}	-0.02 (-0.10, 0.05) p=0.54	0.11	-0.12 (-0.20, -0.04) p=5×10^{-3}
n-3 fatty acids (%)	-3×10^{-3} (-0.03, 0.02) p=0.83	0.03 (-1×10^{-3}, 0.06) p=0.06	0.09	3×10^{-3} (-0.02, 0.03) p=0.84
n-6 fatty acids (%)	-0.12 (-0.20, -0.03) p=0.01	-0.03 (-0.11, 0.06) p=0.52	0.13	-0.11 (-0.19, -0.02) p=0.01
PUFA (%)	-0.12 (-0.21, -0.03) p=0.01	3×10^{-5} (-0.09, 0.09) p=1.00	0.07	-0.10 (-0.20, -0.01) p=0.03
MUFA (%)	0.11 (0.02, 0.20) p=0.02	0.07 (-0.02, 0.16) p=0.14	0.55	0.07 (-0.01, 0.15) p=0.07
Saturated fatty acids (%)	0.01 (-0.07, 0.08) p=0.83	-0.07 (-0.14, 2×10^{-3}) p=0.06	0.13	0.03 (-0.02, 0.08) p=0.27
**Glycolysis related metabolites**				
Glucose (mmol/l)	-3×10^{-3} (-0.02, 0.02) p=0.78	0.01 (-0.01, 0.03) p=0.40	0.36	-0.01 (-0.03, 0.02) p=0.61
Lactate (mmol/l)	2×10^{-3} (-0.01, 0.01) p=0.71	-3×10^{-3} (-0.02, 0.01) p=0.67	0.56	2×10^{-3} (-0.01, 0.02) p=0.72
Pyruvate (mmol/l)	-1×10^{-4} (-9×10^{-4}, 6×10^{-4}) p=0.76	-3×10^{-4} (-1×10^{-3}, 5×10^{-4}) p=0.46	0.76	3×10^{-4} (-5×10^{-4}, 1×10^{-3}) p=0.45
Citrate (mmol/l)	-9×10^{-5} (-9×10^{-4}, 7×10^{-4}) p=0.82	-1×10^{-3} (-2×10^{-3}, -2×10^{-4}) p=0.01	0.07	-7×10^{-4} (-1×10^{-3}, -2×10^{-4}) p=0.01
**Amino acids**				

S6 Table. NFBC66 and NFBC86: offspring lipoproteins, lipids and metabolites absolute concentration differences per 1-SD higher parental BMI.

	NFBC86			NFBC66
Metabolites	mother	father	P_{boot}	mother
Alanine (mmol/l)	-6×10^{-4} (-3×10^{-3}, 2×10^{-3}) p=0.60	-4×10^{-4} (-3×10^{-3}, 2×10^{-3}) p=0.72	0.90	1×10^{-3} (-9×10^{-4}, 4×10^{-3}) p=0.21
Glutamine (mmol/l)	-6×10^{-4} (-3×10^{-3}, 2×10^{-3}) p=0.63	-2×10^{-3} (-4×10^{-3}, 6×10^{-4}) p=0.14	0.46	-3×10^{-3} (-0.01, 2×10^{-4}) p=0.07
Histidine (mmol/l)	5×10^{-5} (-3×10^{-4}, 4×10^{-4}) p=0.77	2×10^{-4} (-2×10^{-4}, 6×10^{-4}) p=0.29	0.58	-3×10^{-5} (-4×10^{-4}, 4×10^{-4}) p=0.90
Branched-chain amino acids				
Isoleucine (mmol/l)	5×10^{-4} (-4×10^{-6}, 9×10^{-4}) p=0.05	8×10^{-4} (4×10^{-4}, 1×10^{-3}) p=3×10^{-4}	0.26	7×10^{-4} (2×10^{-4}, 1×10^{-3}) p=2×10^{-3}
Leucine (mmol/l)	5×10^{-4} (-3×10^{-5}, 1×10^{-3}) p=0.06	1×10^{-3} (6×10^{-4}, 2×10^{-3}) p=7×10^{-5}	0.13	1×10^{-3} (5×10^{-4}, 2×10^{-3}) p=4×10^{-4}
Valine (mmol/l)	8×10^{-4} (-5×10^{-4}, 2×10^{-3}) p=0.23	2×10^{-3} (9.7×10^{-4}, 4×10^{-3}) p=7×10^{-4}	0.11	2×10^{-3} (5×10^{-4}, 3×10^{-3}) p=0.01
Aromatic amino acids				
Phenylalanine (mmol/l)	7×10^{-4} (3×10^{-4}, 1×10^{-3}) p=6×10^{-4}	5×10^{-4} (9×10^{-5}, 9×10^{-4}) p=0.01	0.49	1×10^{-3} (7×10^{-4}, 2×10^{-3}) p=4×10^{-7}
Tyrosine (mmol/l)	1×10^{-4} (-3×10^{-4}, 6×10^{-4}) p=0.54	4×10^{-4} (-1×10^{-5}, 9×10^{-4}) p=0.06	0.33	5×10^{-4} (1×10^{-4}, 9×10^{-4}) p=0.01
**Ketone bodies**				
Acetate (mmol/l)	9.6×10^{-5} (-3×10^{-4}, 5×10^{-4}) p=0.60	-4×10^{-5} (-4×10^{-4}, 3×10^{-4}) p=0.84	0.56	-4×10^{-4} (-1×10^{-3}, 3×10^{-4}) p=0.29
Acetoacetate (mmol/l)	2×10^{-3} (-1×10^{-4}, 5×10^{-3}) p=0.06	6×10^{-4} (-1×10^{-3}, 2×10^{-3}) p=0.46	0.19	-2×10^{-3} (-3×10^{-3}, -5×10^{-4}) p=0.01
Beta-hydroxybutyrate (mmol/l)	2×10^{-3} (-2×10^{-3}, 0.01) p=0.35	1×10^{-3} (-3×10^{-3}, 0.01) p=0.55	0.77	-3×10^{-3} (-0.01, -6×10^{-5}) p=0.05
**Fluid balance**				
Creatinine (mmol/l)	2×10^{-4} (-2×10^{-4}, 5×10^{-4}) p=0.34	4×10^{-4} (4×10^{-5}, 7×10^{-4}) p=0.03	0.36	6×10^{-5} (-3×10^{-4}, 5×10^{-4}) p=0.75
Albumin (signal area)	2×10^{-4} (-2×10^{-4}, 7×10^{-4}) p=0.34	3×10^{-5} (-4×10^{-4}, 5×10^{-4}) p=0.88	0.53	-3×10^{-4} (-8×10^{-4}, 2×10^{-4}) p=0.31
**Inflammation**				
Glycoprotein acetyls (mmol/l)	0.01 (4×10^{-3}, 0.02) p=3×10^{-3}	0.01 (3×10^{-3}, 0.02) p=0.01	0.70	0.01 (4×10^{-3}, 0.02) p=4×10^{-3}

Associations were adjusted for parental age, smoking, education, head of household social class, maternal parity, offspring's age at blood collection and sex.
S6 Table correspond to the SD-scaled metabolite concentration shown in S3 Fig.
