# Supplementary material for: Association of pre-pregnancy body mass index with offspring metabolic profile: Analyses of 3 European prospective birth cohorts
Source: PLoS Med. 2017 Aug 22;14(8):e1002376. doi: 10.1371/journal.pmed.1002376 (PMC5568725; doi:10.1371/journal.pmed.1002376)
Supplement: S1 Text — (PDF) [file pmed.1002376.s014.pdf]

S1 Text. *Supplemental methods.*

Study populations

Avon Longitudinal Study of Parents and Children (ALSPAC)

The ALSPAC is a prospective pregnancy/birth cohort that was established to understand how genetic and environmental characteristics influence health and development in parents and children [1, 2]. During 1990-92, recruitment sought to enrol pregnant women resident in Avon, South West of England, with an expected date of delivery between 1st April 1991 and 31st December 1992. Of the 14,541 pregnancies originally enrolled there were 14,062 live births of whom 13,988 were still alive after 12 months [2]. These women and their offspring have been followed over more than two decades, have completed up to 50 questionnaires, had repeat detailed clinical assessments and have had data abstracted from their medical and educational records [1, 2]. Parental pre-pregnancy weight, height, education, occupation and smoking behaviour, and maternal parity were obtained during pregnancy via questionnaires. Information on partners has been collected in one of two ways: either by responses from the mother about their partner's or by responses of the partners themselves when mothers have passed questionnaires on to them [1]. Their weight and height was reported by the mother's during early pregnancy. Offspring sex was obtained from obstetric records and parental and offspring ages were calculated from their dates of birth and dates of questionnaires or clinic assessments. Parental occupation was classified into social class groups from I (managerial) to IV (unskilled manual workers). Highest educational qualification for both parents was collapsed into one of five categories from none/Certificate of Secondary Education (CSE; national school exams at age 16) to university degree.

The study website contains details of all the data that are available through a fully searchable data dictionary (http://www.bristol.ac.uk/alspac/researchers/access). Ethical approval for the study was obtained from the ALSPAC Ethics and Law Committee and the Local Research Ethics Committees (full details at http://www.bristol.ac.uk/alspac/researchers/data-access/ethics/lrec-approvals/#d.en.164120). Parents provided written informed consent regarding their own participation. The main caregiver initially provided consent for child participation and from the age 16 years the offspring themselves have provided informed written consent.

NMR-based metabolomics was measured in 2013 from stored EDTA plasma samples (-80 °C) [2] for 4139 adolescents collected at the 2006-2007 and 2008-2009 follow-up clinics. At both assessments, adolescents attended after an overnight fast or minimum 6 hours fast. After excluding 1 analytical outlier, 2401-2440 mother-father-offspring trios were available for analysis (see S1 Fig).

Northern Finland Birth Cohort 1966 and 1986 (NFBC66 and NFBC86)

The Northern Finland Birth Cohort studies (http://www.oulu.fi/nfbc) are two longitudinal birth cohorts established to study factors affecting preterm birth and consequent morbidity in the two northernmost provinces of Finland, Oulu and Lapland. The NFBC66 includes 12,058 live births (12,231 children) covering 96% of all eligible births in this region during January-December 1966. Two decades later, a second cohort of 9,432 births (9,479 children) was obtained (NFBC86) which covered 99% of all the deliveries taking place in the target regions during July 1985-June 1986. In both cohorts, mothers and children have been followed-up since mothers enrolled at their first antenatal clinic visit (10-16th week). For NFBC86, the 16-year follow-up data collection (2001-2002) included clinical examination and serum collection for 6621 adolescents (71% of the original cohort) [3, 4]. In NFBC86: parental height, weight, occupation, smoking status, offspring sex and maternal parity were collected using questionnaires given to all mothers at their first antenatal clinic visit. Level of education was

obtained from questionnaires in 2001-02. Parental and offspring age was derived from their date of birth and date of assessments. Parental education was categorized into 8 categories from no occupational education to University degree, and occupation into 6 categories from entrepreneur to no-occupation. In NFBC66: maternal height, weight, occupation, smoking status, parity, child sex were reported by mothers at the first antenatal clinic visit (16th week of gestation), or in questionnaires administered between the 24th and 28th week of gestation. Offspring age at serum collection was derived from their date of birth and date of attendance at the 1997-1998 follow-up clinic. Maternal age in pregnancy was derived from year of birth and the date of pregnancy questionnaire completion. Education was categorized into 9 categories from none or circulating school to beyond matriculation exam and occupation into 5 categories ranging from I (highest social class) to V (no-occupation). Information on paternal BMI was not collected in NFBC66.

Informed written consent was obtained from all participants. The research protocols were approved by the Ethics Committee of Northern Ostrobotnia Hospital District, Finland.

NMR-based metabolomics was measured on 5500 adolescents, of which 95% were overnight fasting serum. In NFBC66, the 1997 follow-up also included clinical examinations and serum sampling for 6007 participants aged 31 years (52% of target sample) [5]. This subsample was representative of the original cohort. In total, 5714 participants had NMR-based metabolite profiles measured, of which 96% were performed on overnight fasting serum [6]. In both cohorts, serum samples were stored at -80 °C until thawing NMR profiling in 2012.

Variable harmonization across cohorts

For the one-stage individual participant analysis (IPD) meta-analysis, education and head of house hold social class occupation categories were harmonized (see S1 Table). The former was collapse into basic or none, secondary, higher and other, and the latter into I (highest social class) to IV (lowest social class).

Metabolic NMR profiling

Each lipoprotein measurement is characterized by three elements: size (e.g. extremely large, very large, large, medium, small, very small), density (e.g. very low density lipoprotein (VLDL), Intermediate density lipoprotein (IDL), low density lipoprotein (LDL), high density lipoprotein (HDL)) and property (e.g. particle concentration, total lipids, triglycerides, phospholipids, total cholesterol, cholesterol esters, free cholesterol). The 14 lipoprotein subclass sizes were defined as follows: VLDL is subdivided into six subclasses, the largest being extremely large VLDL with particle diameters from 75 nm upwards and a possible contribution of chylomicrons, and five other VLDL subclasses (average particle diameters of 64.0 nm, 53.6 nm, 44.5 nm, 36.8 nm, and 31.3 nm); IDL (28.6 nm), three LDL subclasses (25.5 nm, 23.0 nm, and 18.7 nm), and four HDL subclasses (14.3 nm, 12.1 nm, 10.9 nm, and 8.7 nm) [7]. The mean sizes for VLDL, LDL and HDL particles were calculated by weighting the corresponding subclass diameters with their particle concentrations.

The lipoproteins traits obtained are the concentration of the lipoprotein size-density-property combination in the total serum sample. For example, 0.5 mmol/l of very large VLDL cholesterol means 0.5 mmol of cholesterol embedded in very large VLDL particles per litre of serum. Remnant cholesterol was defined as VLDL-cholesterol + IDL-cholesterol, which is equivalent to total-cholesterol - HDL-cholesterol - LDL-cholesterol [7]. For fatty acids (FA), only the *cis* configuration was quantified since the *trans* fatty acids are below the platform's detection limit [7]. FA were modelled as individual (absolute) measures and also as ratios (expressed as a %) to total FAs.

Statistical analyses, multiple testing correction

Principal component analysis (PCA) was performed separately on each individual cohort. In each cohort, all offspring who had data on the metabolic traits were used and PCA was performed on the z-scored metabolic data. This method assumes that the independence of the principal components (PCs) is equivalent to the degree of freedom of the original metabolic dataset, and that retaining a number of PCs that is enough to explain at least 95% of the variance will only result in a small chance of a type 1 error. Since the number of samples available varies across cohorts, the number of PCs needed to explain 95% of the variation in the metabolic traits also varies. The PCA results are as follows: ALSPAC: 2440 observations, 17 PCs; NFBC66: 4874 observations, 14 PCs; NFBC86: 2937 observations; 15 PCs. The highest number (17 components) was observed in the ALSPAC cohort and it was used as a conservative estimate of the number of independent tests been performed. Therefore, the threshold of p-value < 0.05 becomes p-value < 0.003 (i.e. $\alpha/17$ where $\alpha=0.05$), when multiple testing is considered, for assessing associations with the 153 metabolic traits.

References

1. Fraser A, Macdonald-Wallis C, Tilling K, Boyd A, Golding J, Davey Smith G, et al. Cohort Profile: The Avon Longitudinal Study of Parents and Children: ALSPAC mothers cohort. Int J Epidemiol. 2013;42(1):97-110. doi: 10.1093/ije/dys066. PubMed PMID: WOS:000316699300011.
2. Boyd A, Golding J, Macleod J, Lawlor DA, Fraser A, Henderson J, et al. Cohort Profile: The 'Children of the 90s'-the index offspring of the Avon Longitudinal Study of Parents and Children. Int J Epidemiol. 2013;42(1):111-27. doi: 10.1093/ije/dys064. PubMed PMID: WOS:000316699300012.
3. Veltsista A, Laitinen J, Sovio U, Roma E, Jarvelin M-R, Bakoula C. Relationship between eating behavior, breakfast consumption, and obesity among Finnish and Greek adolescents. J Nutr Educ Behav. 2010;42(6):417-21. PubMed PMID: Medline:20729150.
4. Kantomaa MT, Stamatakis E, Kankaanpaa A, Kaakinen M, Rodriguez A, Taanila A, et al. Physical activity and obesity mediate the association between childhood motor function and adolescents' academic achievement. Proc Natl Acad Sci U S A. 2013;110(5):1917-22. doi: 10.1073/pnas.1214574110. PubMed PMID: 23277558; PubMed Central PMCID: PMCPMC3562837.
5. Sabatti C, Service SK, Hartikainen A-L, Pouta A, Ripatti S, Brodsky J, et al. Genome-wide association analysis of metabolic traits in a birth cohort from a founder population. Nature genetics. 2009;41(1):35-46. PubMed PMID: Medline:19060910.
6. Wurtz P, Makinen V-P, Soininen P, Kangas AJ, Tukiainen T, Kettunen J, et al. Metabolic signatures of insulin resistance in 7,098 young adults. Diabetes. 2012;61(6):1372-80. PubMed PMID: Medline:22511205.
7. Wurtz P, Kangas AJ, Soininen P, Lehtimaki T, Kahonen M, Viikari JS, et al. Lipoprotein subclass profiling reveals pleiotropy in the genetic variants of lipid risk factors for coronary heart disease: a note on Mendelian randomization studies. J Am Coll Cardiol. 2013;62(20):1906-8. doi: 10.1016/j.jacc.2013.07.085. PubMed PMID: 24055740.
