# Supplementary material for: Association of pre-pregnancy body mass index with offspring metabolic profile: Analyses of 3 European prospective birth cohorts
Source: PLoS Med. 2017 Aug 22;14(8):e1002376. doi: 10.1371/journal.pmed.1002376 (PMC5568725; doi:10.1371/journal.pmed.1002376)
Supplement: S2 Text — (PDF) [file pmed.1002376.s015.pdf]

S2 Text. *Statistical analysis plan.*

Statistical analysis

Body Mass Index (exposure) and covariates

- All statistical analyses to be conducted using BMI, parental age, maternal parity, and offspring's age at blood collection as continuous variables.
- Potential confounders to adjust models for: parental age, smoking, education, head of household social class, maternal parity, offspring's age at blood collection and sex.

Metabolite data (outcome)

- Exclude ratio metabolic traits with exception of fatty acid ratios.
- Define the list of outcomes (metabolic traits): check the overlap between the different cohorts (not all cohorts have the same metabolites measured).
- Quality control of metabolic traits data: plot concentration or % *versus* observation number (or box plots) and check for outliers. Do not remove any outliers in the first instance unless there is a clear indication that it is an actual artifact in the data (i.e. observations lying orders or magnitude away from the cloud of data). Perform sensitivity analysis for the other outliers.
- Establish a p-value threshold considering multiple testing and the correlation structure of the metabolic data: perform Principal Component Analysis (PCA) on *z*-scored metabolic trait data and extract the number of principal components (*A*) that explain at least 95% of the variance, the corrected p-value threshold is given by alpha/*A* where alpha=0.05.

Relationship of parental BMI and metabolic traits

- Analysis to be performed on trios of mother-father-offspring who have no missing data on at least one trio of exposures/outcome/covariables.
- Models will be computed for standardized (z-scored) and un-standardized metabolic traits.
- Conduct a **one-stage** and a **two-stage** individual participant data (IPD) meta-analysis.
- Two-stage IPD meta-analysis: perform all analyses individually on each cohort and for mothers and fathers separately.

- Standardize metabolic traits separately for each cohort and for mothers and fathers.
- Use linear regression with robust standard errors to estimate associations between **parental BMI** and offspring standardized (z-scored) and unstandardized metabolic traits. Adjust models for parental age, smoking, education, head of household social class, maternal parity, offspring's age at blood collection and sex.
- Combine the results of each linear regression in each cohort and parent, test for heterogeneity (I^2) and meta-analyze the results using random effects for mothers and fathers separately.

- **One-stage IPD meta-analysis**: Vertically concatenate individual cohort data after variable harmonization.
  - Standardize metabolic traits across cohorts. Standardize paternal BMI across cohorts, separately for mothers and fathers.
  - Compute adjusted models using linear regression with robust standard errors for mothers and fathers separately, include a dummy variable indicating cohort membership.
  - Use bootstrap (1000 replications) to compute the difference between the associations of mother and father's BMI with outcomes.

Data visualization and results presentation

- Produce a flow diagram of participant inclusion in the study and a descriptive table with information regarding each cohort.
- Produce forest plots of the results, from the association between parental BMI with **standardized** metabolic traits, for individual cohort analysis, **one** and **two-stage** IPD meta-analysis.
- Produce a scatter plot with regression line of the results of two-stage IPD *versus* one-stage IPD separately for mother and father, and one-stage IPD father *versus* mother.
- Produce tables with results, of the association between parental BMI with **unstandardized** metabolic traits, for individual cohort analysis, one and two-stage IPD meta-analysis.
