# Supplementary material for: Association of pre-pregnancy body mass index with offspring metabolic profile: Analyses of 3 European prospective birth cohorts
Source: PLoS Med. 2017 Aug 22;14(8):e1002376. doi: 10.1371/journal.pmed.1002376 (PMC5568725; doi:10.1371/journal.pmed.1002376)
Supplement: S3 Text — (PDF) [file pmed.1002376.s016.pdf]

STROBE Statement—Checklist of items that should be included in reports of ***cohort studies***

	Item No	Recommendation
**Title and abstract**	1	(*a*) Indicate the study's design with a commonly used term in the title or the abstract **Title**, "*Association of pre-pregnancy body mass index with offspring metabolic profile: three European prospective birth cohort analysis"*
		(*b*) Provide in the abstract an informative and balanced summary of what was done and what was found **Abstract, Methods and Findings**: *"We used one and two-stage individual participant data (IPD) meta-analysis, and a negative-control (paternal BMI) to examine the association between maternal pre-pregnancy BMI and offspring serum metabolome from three European birth cohorts (offspring age at metabolite assessment 16, 17 and 31 years). (...) Results from one-stage IPD meta-analysis (N=5327 to 5377 mother-father-offspring trios) showed that increasing maternal and paternal BMI was associated with an adverse cardio-metabolic profile in offspring. (...) Slightly stronger magnitudes of associations were present for maternal compared with paternal BMI across these associations, however there was no strong statistical evidence for heterogeneity between them (all bootstrap P > 0.003, equivalent to P > 0.05 after accounting for multiple testing). (...) Limitations of this study are that inferences cannot be drawn about the role of circulating maternal fetal fuels (i.e. glucose, lipids, fatty acids and amino acids) on later offspring metabolic profile. In addition, BMI may not reflect potential effects of maternal pregnancy fat distribution."*
**Introduction**		
Background/rationale	2	Explain the scientific background and rationale for the investigation being reported **Introduction, paragraph 1**: *"In Western populations, the proportion of women who start pregnancy overweight/obese (BMI ≥25Kg/m2) has increased over the last 20-30 years and is now estimated to be between 20-50% [1, 2]. The developmental origin of adult diseases hypothesis proposes that maternal greater adiposity in pregnancy can prime changes in fetal metabolism that result in a life-long risk of greater adiposity and metabolic dysregulation [3]. (...) This may perpetuate obesity and adverse cardio-metabolic outcomes across generations, as the daughters of overweight women would be predisposed to enter pregnancy overweight and with adverse metabolic profiles themselves. Concerns about this hypothesis are influencing antenatal care, (...). However, whether the associations of maternal adiposity and associated traits with offspring outcomes are causal is unknown, and if they are causal, then the mechanisms are unclear [4, 6, 7]."*
Objectives	3	State specific objectives, including any prespecified hypotheses **Introduction, paragraph 3**: *"The aim of this study was to examine associations between maternal pre-pregnancy BMI and multiple offspring serum lipids, lipoproteins and metabolites in adolescence and adulthood (i.e. when offspring are in their reproductive years), using paternal BMI as a negative control. Our hypotheses are that two key paths could explain the association of maternal early/pre-pregnancy BMI with offspring future metabolic traits: (i) intrauterine developmental overnutrition and (ii) confounding of this potential effect via shared familial genetics, socioeconomic, lifestyle and behavioral characteristics (Fig 1). (...)Thus, if maternal associations represent causal intrauterine effects, as opposed to being due to shared familial factors they should be stronger than paternal associations with the same outcomes [4]."*
**Methods**		
Study design	4	Present key elements of study design early in the paper **Methods, Study populations, paragraph 2:** *"The parent specific BMI associations with offspring metabolic profiles were examined in one British and two Finnish birth cohorts with nuclear magnetic resonance (NMR)-based serum metabolomics: Avon Longitudinal Study of Parents and Children [12, 13] (ALSPAC; offspring follow-up at 17 years), Northern Finland Birth Cohort 1966 and 1986 studies [14, 15] (NFBC66 and NFBC86; offspring follow-up at 31 and 16 years, respectively). Full details of the cohorts are provided in the Supporting Information S1 Text and S1 Fig."*

Setting	5	Describe the setting, locations, and relevant dates, including periods of recruitment, exposure, follow-up, and data collection.<br>**Methods, Study populations, paragraph 2: "***The parent specific BMI associations with offspring metabolic profiles were examined in one British and two Finnish birth cohorts (...)"*<br>**Supporting Information S1 Text:** *"The ALSPAC is a prospective pregnancy/birth cohort that was established to understand how genetic and environmental characteristics influence health and development in parents and children [1, 2]. During 1990-92, recruitment sought to enrol pregnant women resident in Avon, South West of England, with an expected date of delivery between 1st April 1991 and 31st December 1992. Of the 14,541 pregnancies originally enrolled there were 14,062 live births of whom 13,988 were still alive after 12 months [2]. These women and their offspring have been followed over more than two decades, have completed up to 50 questionnaires, had repeat detailed clinical assessments and have had data abstracted from their medical and educational records [1, 2]. Parental pre-pregnancy weight, height, education, occupation and smoking behaviour, and maternal parity were obtained during pregnancy via questionnaires. Information on partners has been collected (...)" and "The Northern Finland Birth Cohort studies (http://www.oulu.fi/nfbc/) are two longitudinal birth cohorts established to study factors affecting preterm birth and consequent morbidity in the two northernmost provinces of Finland, Oulu and Lapland. The NFBC66 includes 12,058 live births (12,231 children) covering 96% of all eligible births in this region during January-December 1966. Two decades later, a second cohort of 9,432 births (9,479 children) was obtained (NFBC86) which covered 99% of all the deliveries taking place in the target regions during July 1985-June 1986 (...)"*
Participants	6	(*a*) Give the eligibility criteria, and the sources and methods of selection of participants. Describe methods of follow-up<br>**Supporting Information S1 Text:** *"During 1990-92, recruitment sought to enrol pregnant women resident in Avon, South West of England, with an expected date of delivery between 1st April 1991 and 31st December 1992."* and *"The NFBC66 includes 12,058 live births (12,231 children) covering 96% of all eligible births in this region during January-December 1966. Two decades later, a second cohort of 9,432 births (9,479 children) was obtained (NFBC86) which covered 99% of all the deliveries taking place in the target regions during July 1985-June 1986. In both cohorts, mothers and children have been followed-up since mothers enrolled at their first antenatal clinic visit (10-16th week)."*
		(*b*) For matched studies, give matching criteria and number of exposed and unexposed<br>**Not applicable**
Variables	7	Clearly define all outcomes, exposures, predictors, potential confounders, and effect modifiers. Give diagnostic criteria, if applicable<br>**Methods, Parental exposures and covariables and Outcomes: metabolic profiling sections:**<br>**"Parental exposures and covariables**<br>*In ALSPAC: parental pre-pregnancy weight, height, education, occupation and smoking behaviour (...)"*<br>*"***Outcomes: metabolic profiling**<br>*A comprehensive profiling of offspring circulating lipids and metabolites was done by (...)"*<br>*"We performed two additional analyses (...) that aimed to explore whether any maternal (or paternal) pre-pregnancy BMI associations with offspring metabolites were mediated by the relationship of the parent's BMI with offspring BMI."*
Data sources/ measurement	8*	For each variable of interest, give sources of data and details of methods of assessment (measurement). Describe comparability of assessment methods if there is more than one group<br>**Methods, Parental exposures and covariables, paragraphs 1-3; Methods, Outcomes: metabolic profiling; and *Supporting Information S1 Text:***<br>*"In ALSPAC: parental pre-pregnancy weight, (...) were obtained during pregnancy via questionnaires. Offspring sex was obtained from obstetric records and parental and offspring (...)"*<br>*"In NFBC86: parental height, weight, occupation (...) were collected using questionnaires given to all mothers at their first antenatal clinic visit. (...)"*

		"In NFBC66: maternal height, weight, occupation, smoking status, parity, child sex were reported by mothers at the first antenatal clinic visit (...)"<br>*"**Outcomes: metabolic profiling***<br>*A comprehensive profiling of offspring circulating lipids and metabolites was done by a high-throughput NMR metabolomics platform (...) In ALSPAC, offspring metabolite data were assessed on fasting (minimum 6-hours) plasma at two ages (mean age 15.5 and 17.8) and we used data from either of these. (...) Participants of both NFBCs fasted overnight before serum collection on the morning of clinic attendance"*
Bias	9	Describe any efforts to address potential sources of bias<br>**Methods, Statistical analysis, paragraphs 2-6:** *"One-stage and two-stage IPD [16-18] meta-analyses were performed to assess the associations (...)"; "(...) using paternal BMI as a negative control (...)"; "Linear regression models were adjusted for (...)"; "Robust standard errors were estimated for all associations and probability values as some metabolite concentrations had skewed distributions."; "We conducted three sets of IPD meta-analysis that included maternal vs paternal comparisons: (...)"; "To establish a threshold that takes into account multiple testing and the correlation structure of the metabolite data (...)"; "In addition, the magnitudes of maternal and paternal associations were compared to each other using linear fit, and a random intercept and slope multilevel model.(...)"; "We performed two additional analyses (...) that aimed to explore whether any maternal (or paternal) pre-pregnancy BMI associations with offspring metabolites were mediated by the relationship of the parent's BMI with offspring BMI."; "The differences between maternal and paternal associations (in all three approaches) were calculated from the bootstrap replicate distribution (...)"*
Study size	10	Explain how the study size was arrived at<br>**Methods, Parental exposures and covariables, paragraph 4*:*** *"For our analyses, we used data from 5327 to 5377 mother-father-offspring trios from ALSPAC and NFBC86, and 4841 to 4874 mother-offspring pairs from NFBC66 who had data on parental BMI, offspring metabolite and covariables. (...)"*<br>**Supporting Information S1 Fig:** *shows the flowchart*
Quantitative variables	11	Explain how quantitative variables were handled in the analyses. If applicable, describe which groupings were chosen and why<br>**Methods, Parental exposures and covariables, paragraph 1-3, and *Supporting Information, S1-2 Table, and S1 Text:***<br>*"In ALSPAC: (...) Parental occupation was classified into social class groups from I (managerial) to IV (unskilled manual workers). Highest educational qualification for both parents was collapsed into one of five categories from none/Certificate of Secondary Education (CSE; national school exams at age 16) to university degree."; "In NFBC86: (...) Parental education was categorized into 8 categories from no occupational education to University degree, and occupation into 6 categories from entrepreneur to no-occupation."; "In NFBC66: (...) Education was categorized into 9 categories from none or circulating school (...)"; "In all cohorts, head of household social class was defined as the highest occupation of either parent.";*<br>***"Variable harmonization across cohorts***<br>*For the one-stage individual participant analysis (IPD) meta-analysis, education and head of house hold social class occupation categories were harmonized (...)"*<br><br>*"(...) random intercept and slope multilevel model. (...) We defined clusters according to metabolic trait-classes, which resulted in 27 classes with between 1 and 9 metabolic traits in each class (see S2 Table)."*
Statistical methods	12	(*a*) Describe all statistical methods, including those used to control for confounding<br>**Methods, Statistical analysis, paragraphs 2-6*:*** *"One-stage and two-stage IPD [16-18] meta-analyses were performed to assess the associations of maternal pre-pregnancy BMI with offspring metabolic profiles, using paternal BMI as a negative control."*<br>*"Linear regression models were adjusted for parental age, smoking, education, head of household social class, maternal parity, offspring age at blood collection and sex. Robust standard errors were estimated for all associations and probability values as some metabolite concentrations had skewed distributions. "We conducted three sets of IPD meta-analysis that included maternal vs paternal comparisons: (...)"; "To establish a threshold that takes into account multiple testing and the correlation*

		structure of the metabolite data (...)"; "We performed two additional analyses (...) that aimed to explore whether any maternal (or paternal) pre-pregnancy BMI associations with offspring metabolites were mediated by the relationship of the parent's BMI with offspring BMI.";<br>*"The differences between maternal and paternal associations (in all three approaches) were calculated from the bootstrap replicate distribution (...)"*
		(*b*) Describe any methods used to examine subgroups and interactions<br>**Not applicable**
		(*c*) Explain how missing data were addressed<br>**Methods, Parental exposures and covariables, paragraph 4*:*** *"For our analyses, we used data from 5327 to 5377 mother-father-offspring trios from ALSPAC and NFBC86, and 4841 to 4874 mother-offspring pairs from NFBC66 who had data on parental BMI, offspring metabolite and covariables. (...)"*
		(*d*) If applicable, explain how loss to follow-up was addressed<br>**Not applicable**
		(*e*) Describe any sensitivity analyses<br>**Methods, Statistical analysis, paragraphs 3 and 6*:*** *"We conducted three sets of IPD meta-analysis that included maternal vs paternal comparisons: (...)"; "We performed two additional analyses (...) that aimed to explore whether any maternal (or paternal) pre-pregnancy BMI associations with offspring metabolites were mediated by the relationship of the parent's BMI with offspring BMI.";*
**Results**		
Participants	13*	(a) Report numbers of individuals at each stage of study—eg numbers potentially eligible, examined for eligibility, confirmed eligible, included in the study, completing follow-up, and analysed<br>**Supporting Information S1 Fig**
		(b) Give reasons for non-participation at each stage<br>**Supporting Information S1 Fig**
		(c) Consider use of a flow diagram<br>**Supporting Information S1 Fig**
Descriptive data	14*	(a) Give characteristics of study participants (eg demographic, clinical, social) and information on exposures and potential confounders<br>**Results, paragraph 1:** *"Characteristics of the three study populations are shown in S1 Table and the flowchart in S1 Fig. The percentage of overweight/obese mothers was 20%, 15% and 23% in ALSPAC, NFBC86 and NFBC66, respectively and of fathers was 47% and 32% in ALSPAC and NFBC86, respectively. The highest percentage of overweight/obesity (...)"*<br>**Supporting Information S1 Table.** *Characteristics of the three study populations.*<br>**Supporting Information S1 Text:** *"The ALSPAC (...) During 1990-92, recruitment sought to enrol pregnant women resident in Avon, South West of England, with an expected date of delivery between 1st April 1991 and 31st December 1992. (...)" and "The Northern Finland Birth Cohort studies (...) in the two northernmost provinces of Finland, Oulu and Lapland."*
		(b) Indicate number of participants with missing data for each variable of interest<br>**Supporting Information S1 Fig**
		(c) Summarise follow-up time (eg, average and total amount)<br>**Discussed throughout the manuscript** as indicated by age of offspring (exposure is parental BMI at time of pregnancy)
Outcome data	15*	Report numbers of outcome events or summary measures over time<br>**Supporting information S1 Table, S1 Text:** *Outcomes are all continuously measured*<br>**Results, paragraph 2**: *"Fig 2-4 shows associations of parental BMI with the 153 offspring metabolic measures, each expressed as a difference in means in SD units for a 1-SD greater parental BMI, from a one-stage IPD meta-analysis restricted to the two cohorts with complete trio data (N= 5327 to 5377 trios)."*
Main results	16	(*a*) Give unadjusted estimates and, if applicable, confounder-adjusted estimates and

		their precision (eg, 95% confidence interval). Make clear which confounders were adjusted for and why they were included<br>**We present adjusted estimates.**<br>**All forest plots show 95% confidence interval of all point estimates; All tables with estimates provide 95% confidence interval and p-values.**
		(*b*) Report category boundaries when continuous variables were categorized<br>**Supporting Information S1 Table.** *Characteristics of the three study populations.*
		(*c*) If relevant, consider translating estimates of relative risk into absolute risk for a meaningful time period<br>**Results, paragraph 2**: *"Fig 2-4 shows associations of parental BMI with the 153 offspring metabolic measures, each expressed as a difference in means in SD units for a 1-SD greater parental BMI,(...). S3 Table shows the same associations expressed as magnitudes in absolute concentration units (e.g. mmol/l per 1-SD difference in parental BMI)."*
Other analyses	17	Report other analyses done—eg analyses of subgroups and interactions, and sensitivity analyses<br>**Results, paragraphs 8-10:** *"When the one-stage analyses were repeated to compare maternal BMI-offspring metabolite associations (...) in the two cohorts with paternal BMI (...) were essentially the same as those described above; (...) In the two-stage meta-analysis, the associations were highly consistent across the three cohorts (...) Cross-sectional associations of offspring BMI with their metabolite concentrations had very similar patterns (...) When adjusting parental BMI-offspring metabolite associations for offspring BMI, most attenuated markedly, such that many point estimates were in the opposite directions (...)"*
**Discussion**		
Key results	18	Summarise key results with reference to study objectives<br>**Discussion, paragraphs 1-2:** *"We report the first study that investigates the potential influence of maternal pre-pregnancy BMI on adult offspring serum metabolome, in order to determine whether intrauterine mechanisms, related to developmental overnutrition, result in metabolic disruption in adults when they are in their reproductive years. We found (...) rather than an intrauterine programming effect explain the associations of maternal pre-pregnancy BMI with offspring metabolites. (...) Lastly, we found that (...) which in turn results in metabolic disruption."*
Limitations	19	Discuss limitations of the study, taking into account sources of potential bias or imprecision. Discuss both direction and magnitude of any potential bias<br>**Discussion, paragraph 5:** *"Limitations of our study include the use of parental BMI as a measure of adiposity; (...) Our findings cannot be used to draw inferences about the role of maternal specific nutritional intakes (...) However, any impact of non-paternity would be likely to selectively reduce paternal BMI associations, since (...) This would thus tend to enhance maternal-paternal differences, whereas we see similar associations between parents. Despite adjusting for several potential confounders, residual confounding (...) Furthermore, our results may not necessarily generalise to other non-European populations."*
Interpretation	20	Give a cautious overall interpretation of results considering objectives, limitations, multiplicity of analyses, results from similar studies, and other relevant evidence<br>**Discussion, paragraphs 3-5:** *"Previous studies have compared associations of maternal and/or paternal BMI, measured pre/early-pregnancy, with offspring adiposity (...). The only previous study that we were able to identify that compared maternal BMI- to paternal BMI- associations with any outcomes similar. (...) Our study has several strengths. It has a large sample size, included replication testing across three different birth-cohorts (...). Limitations of our study include the use (...) In conclusion, the similarity of association (...) rather than intrauterine mechanisms. Further replication of these findings in other larger studies, including with measured fat distribution in mothers and fathers at the time of the mother's pregnancy would be valuable, though we are not aware of any studies with relevant data currently to be able to do that."*<br>*"*
Generalisability	21	Discuss the generalisability (external validity) of the study results<br>**Discussion, paragraph 5:** "*However, we cannot exclude a threshold effect (...) Furthermore, our results may not necessarily generalise to other non-European*

		populations. "<br>**Abstract, Methods and Findings:** *"Limitations of this study are that inferences cannot be drawn about the role of circulating maternal fetal fuels (i.e. glucose, lipids, fatty acids and amino acids) on later offspring metabolic profile. In addition, BMI may not reflect potential effects of maternal pregnancy fat distribution."*
**Other information**		
Funding	22	Give the source of funding and the role of the funders for the present study and, if applicable, for the original study on which the present article is based<br>The following statement was included in the online submission system:<br>*"This study was funded by the European Research Council under the European Union's Seventh Framework Programme (FP7/2007-2013)/ERC grant agreement (Grant number 669545; DevelopObese) and the US National Institute of Health (R01 DK10324). The UK Medical Research Council and the Wellcome Trust (Grant ref: 102215/2/13/2) and the University of Bristol provide core support for ALSPAC. DLSF, DAL, GDS and MA-K, work in a Unit that receives funds from the University of Bristol and the UK Medical Research Council (MC_UU_12013/1, MC_UU_12013/5) and DAL is a UK National Institute of Health Research Senior Investigator (NF-SI-0166-10196). MA-K was supported by the Sigrid Juselius Foundation and the Strategic Research Funding from the University of Oulu. DMW is funded by a European Union Horizon 2020 research and innovation program grant (agreement 634821). The views expressed in this article are those of the authors and not necessarily any funding body. "*

*Give information separately for exposed and unexposed groups.

Note: An Explanation and Elaboration article discusses each checklist item and gives methodological background and published examples of transparent reporting. The STROBE checklist is best used in conjunction with this article (freely available on the Web sites of PLoS Medicine at http://www.plosmedicine.org/, Annals of Internal Medicine at http://www.annals.org/, and Epidemiology at http://www.epidem.com/). Information on the STROBE Initiative is available at http://www.strobe-statement.org.
